# Comparative effectiveness of neutralising monoclonal antibodies in high risk COVID-19 patients: a Bayesian network meta-analysis

**DOI:** 10.1038/s41598-022-22431-6

**Published:** 2022-10-20

**Authors:** David McConnell, Marie Harte, Cathal Walsh, Desmond Murphy, Alistair Nichol, Michael Barry, Roisin Adams

**Affiliations:** 1 National Centre for Pharmacoeconomics, St James’s Hospital, Dublin, Ireland; 2 Department of Pharmacology and Therapeutics, Trinity College Dublin, Dublin, Ireland; 3 Health Research Institute and MACSI, University of Limerick, Limerick, Ireland; 4 Cork University Hospital, Cork, Ireland; 5 University College Cork, Cork, Ireland; 6 St Vincent’s University Hospital, Dublin, Ireland; 7 School of Medicine, University College Dublin, Dublin, Ireland

**Keywords:** Viral infection, Biological therapy, Outcomes research, Statistics

## Abstract

The purpose of this work was to review and synthesise the evidence on the comparative effectiveness of neutralising monoclonal antibody (nMAB) therapies in individuals exposed to or infected with SARS-CoV-2 and at high risk of developing severe COVID-19. Outcomes of interest were mortality, healthcare utilisation, and safety. A rapid systematic review was undertaken to identify and synthesise relevant RCT evidence using a Bayesian Network Meta-Analysis. Relative treatment effects for individual nMABs (compared with placebo and one another) were estimated. Pooled effects for the nMAB class compared with placebo were estimated. Relative effects were combined with baseline natural history models to predict the expected risk reductions per 1000 patients treated. Eight articles investigating four nMABs (bamlanivimab, bamlanivimab/etesevimab, casirivimab/imdevimab, sotrovimab) were identified. All four therapies were associated with a statistically significant reduction in hospitalisation (70–80% reduction in relative risk; absolute reduction of 35–40 hospitalisations per 1000 patients). For mortality, ICU admission, and invasive ventilation, the risk was lower for all nMABs compared with placebo with moderate to high uncertainty due to small event numbers. Rates of serious AEs and infusion reactions were comparable between nMABs and placebo. Pairwise comparisons between nMABs were typically uncertain, with broadly comparable efficacy. In conclusion, nMABs are effective at reducing hospitalisation among infected individuals at high-risk of severe COVID-19, and are likely to reduce mortality, ICU admission, and invasive ventilation rates; the effect on these latter outcomes is more uncertain. Widespread vaccination and the emergence of nMAB-resistant variants make the generalisability of these results to current patient populations difficult.

## Introduction

Several treatments and prophylactic agents directed at COVID-19 have been developed in the midst of the global COVID-19 pandemic. While vaccines remain the backbone of the public health response alongside non-pharmacological measures, there remains a clinical need for those who are at high risk of developing severe COVID-19 disease leading to hospitalisation.

Neutralising monoclonal antibodies (nMABs) are a novel class of antiviral intervention that can bind to and ‘neutralise’ the virus in infected patients. The primary target of these products is the S protein which facilitates target cell binding and fusion by binding to the angiotensin converting enzyme 2 (ACE2) receptor. By directing antibodies to the S protein these nMABs can neutralise the ability of SARS-CoV-2 to bind to the host cell. While nMABs can be derived from the B cells of convalescent patients (convalescent plasma therapy) or from humanized mice (recombinant), it is the recombinant nMABs that this review will focus on. Some products include a combination of two nMABs which may offer better protection than monotherapy as they can bind to two overlapping epitopes, thereby lessening the likelihood of loss of antiviral activity due to variants or escape mutants^1^. Trials for nMABs have included broad populations although some have focussed on exposed or infected cohorts at high risk of developing severe COVID-19. The focus of this review will be on high-risk patients (for which the nMABs currently licensed in the EU are currently indicated). Here we define high risk broadly as those with at least one risk factor such as age > 65 years, BMI > 30 kg/m^2^, or age > 55 years (with cardiovascular disease, hypertension, or chronic respiratory disease). As of end of March 2022 all but one of the nMABs are licensed in Europe. There are currently nMABs products from five different companies which target the surface spike glycoprotein that mediates viral entry into host cells. One product has been withdrawn (bamlanivimab/etesevimab) by the company from the regulatory process.

Given the similar mode of action of nMABs the relative effect of these products is of interest for healthcare decision makers. The application of clinical trial data to populations not studied as well as emerging variants present particular challenges to the decision. An additional obstacle at the time of decision making is the lack of access to robust and full clinical trial data. Decision makers are then faced with a choice, perhaps due to constrained supply and constrained resources, as to what treatments will provide the most benefit for the population. Synthesising evidence in a formal manner as we do in this article can be helpful in identifying the relative benefit of such therapies and where the uncertainties pertaining to their clinical utility lie.

This paper will summarise the evidence on four nMAB products and provide an estimate of comparative efficacy from a network meta-analysis.

## Methodology

A rapid systematic review methodology which is further described in the review protocol (included in Appendix 1) identified evidence from RCTs (or systematic literature review of RCTs) on bamlanivimab/etesevimab (alone or in combination), casirivimab/imdevimab (alone or in combination), regdanvimab, or sotrovimab. At the time of review, the clinical trial data for tixagevimab/cilgavimab were not published and the indication differs from the other nMABs and therefore are not included in our review. Treatments were compared to standard of care (SoC) as well as to each other. The population of interest is those that have been exposed to or have tested positive for COVID-19 who are at high risk of developing severe COVID-19 disease. High risk patients were defined as patients at risk of severe disease due to age, BMI of 30 kg/m^2^ or more, or comorbidities such as hypertension, respiratory disease, or cardiovascular disease. Outcomes of interest for the review included hospitalisation, duration of hospital stays, ICU rates, disease severity outcomes, as determined by the requirement for supplemental oxygen (any O2), mortality, and adverse events (AEs) including serious AEs and infusion-related AEs (where separately reported to all AEs). Searches of bibliographic databases and pre-print sites were undertaken between 27 and 30th of September 2021 and updated on the 14th of December 2021 (outlined in Appendix 2). Citation management, screening, and data extraction were undertaken in-line with the review protocol (Appendix 1). Where outcomes from the same studies and samples were reported in both pre-print and peer-reviewed articles, data from the peer reviewed articles only was utilised in the analysis, except in cases where the peer-reviewed version omitted relevant outcome data previously reported in the pre-print version. A risk of bias assessment was undertaken using the ROB2 tool from Cochrane^2^.

### Data analysis

The evidence for each treatment and outcome was synthesised formally using a Bayesian Network Meta-Analysis (NMA). Due to the sparse data, different doses of the same nMAB or nMAB combination were treated as a single intervention in the evidence network. The feasibility of each outcome-specific NMA was assessed by considering the availability of sufficient data as well as the plausibility of the underlying assumption of similarity (primarily assessed by considering the similarity of the included studies in terms of potential effect-modifiers)^3^. As all feasible networks involved dichotomous outcomes, treatment effects were calculated as relative risks and were synthesised using a generalised linear model (GLM) with a binomial likelihood and a log link function^4^. Random-effects models, fitted as a Bayesian hierarchical model, were used in the base case where possible in order to account for heterogeneity between studies, which includes heterogeneity between different doses of the same nMABs since these were treated as the same intervention. Due to the well-known challenges of carrying out meta-analysis of few studies with low event numbers, particularly in the presence of heterogeneity, weakly informative prior distributions were used (see Appendix 4 supplementary material) for the treatment effect and heterogeneity parameters^5,6^. Specifically, as recommended in Günhan et al.^5^, normal priors with mean 0 and standard deviation 2.82 were used for all relative treatment effects (on the log-relative risk scale): this distribution is symmetric about the null (i.e., a change in risk in either direction is equally likely a-priori) and assumes with 95% prior probability that the effect would not exceed a relative risk of 250 in either direction, which is so large as to be deemed extremely unlikely a priori. For the heterogeneity parameter a half-normal prior with standard deviation 0.5 was used; this gives a 95% prior probability that the effect observed in a random study will differ from the mean effect by factor of less than 3 on the relative risk scale^6^. These priors are weakly informative in the sense that they consider a broad range of treatment effects and heterogeneity to be approximately equally likely a priori, but regard extreme effects to be unlikely. Treatment effects were summarised using posterior medians and 95% credible intervals for the estimated relative risks.

Since the nMABs of interest in this review all share a common mechanism of action (i.e. binding to and neutralising the SARS-CoV-2 virus), we have also separately conducted a scenario analysis in which all nMAB therapies are regarded as a family of similar (but not equivalent) interventions, and estimated the average pooled treatment effect of nMAB therapies versus placebo. To this end, a separate pairwise meta-analysis of nMABs versus placebo was also carried out, again using a random-effects framework to account for the heterogeneity of effects across different therapies. This involved grouping all nMAB therapies as a single intervention and then estimating the pooled nMAB versus placebo effect using a Bayesian random-effects (pairwise) meta-analysis with the same GLM and priors as the base case NMA.

Absolute event risks and risk differences versus placebo/SoC for each treatment and each outcome were also estimated. This was done by pooling the event rates across the placebo/SoC arms of the included studies by fitting (random effects) baseline natural history models^7^, and then applying the relative treatment effects estimated from the NMA. These effects were calculated as expected differences in event numbers per 1000 patients treated (i.e., absolute risk reductions per 1000) for each nMAB compared with placebo/SoC.

Sensitivity analysis was carried out including the use of alternative prior distributions for treatment effect and heterogeneity parameters, fixed-effects models, and the inclusion of a previously excluded study (which enrolled a broader risk population).

Analysis was carried out in R (version 4.1.2)^8^ and JAGS (version 4.3.0)^9^ using the package BUGSnet^10^.

## Results

**Figure 1:**
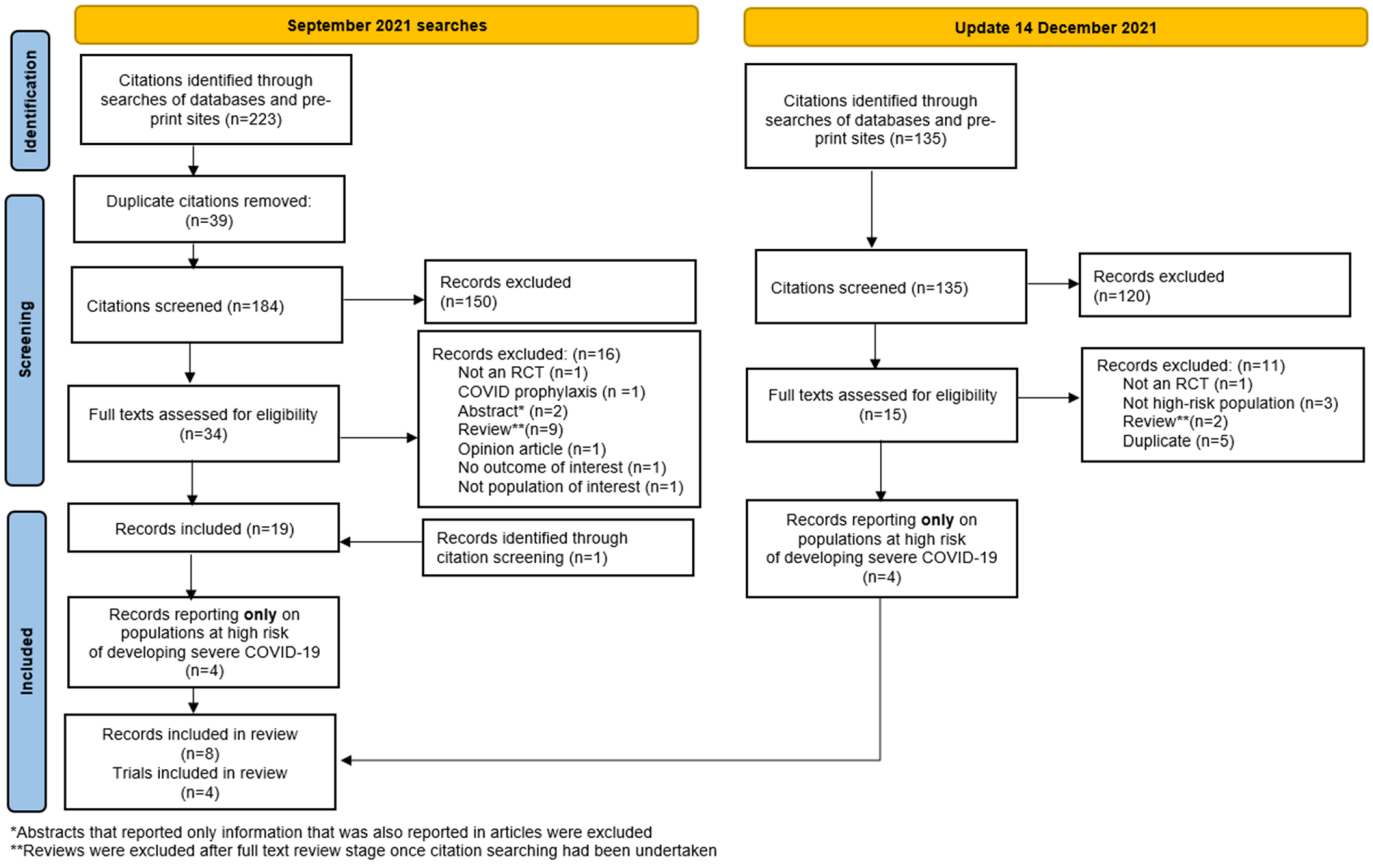
PRISMA flow diagram.

Eight journal articles or pre-prints which reported on RCTs of nMAB treatment in high-risk patients with COVID-19 were selected for inclusion in the analysis (Fig. 1). Three of the articles were peer-reviewed while five were pre-prints. The eight articles reported on four separate studies (outlined in Tables 1 and 2).

**Table 1:** Articles included in review.

Publication/Study	Publication type	Trial location	Sample size^µ^	Setting at baseline	Mean age	% Male	Vaccination status	Variants	Period of data collection	Treatments dose and duration
Weinreich et al. 2021 (c)^11^ Weinreich et al. 2021 (cii)^12^ NCT04425629	Pre-print, Peer reviewed article	United States, Chile, Mexico, Romania	4057	Community	NR	48.7	Patients who received a SARS-CoV-2 vaccine prior to randomization, or had planned use (≤ 90 days) were excluded	NR	24 Sept 2020–17 Jan 2021	Casirivimab/imdevimab 1200 mg, casirivimab/imdevimab 2400 mg, or placebo IV
Dougan et al. 2021^13^ NCT04427501; BLAZE-1	Peer reviewed article	United States	1035	Community	53.8 ± 16.8	48	Amendment k (20/01/2021) of protocol allowed vaccinated participants to be eligible for inclusion	No beta or gamma variants reported	4 Sept 2020–8 Dec 2020	Bamlanivimab 2800 mg and etesevimab 2800 mg, or placebo IV
Gupta et al. 2021^14^ Gupta et al. 2021 (c)^15^ NCT04545060; COMET-ICE	Pre-print, Peer reviewed article	United States, Canada, Brazil, Spain	583	Community	NR	46	Patients who received a SARS-CoV-2 vaccine prior to randomization (at any time point) were excluded	NR	27 Aug 2020–19 Jan 2021	Sotrovimab 500 mg or placebo IV
McCreary et al. 2021^16^ NCT04790786; OPTIMISE-C19	Pre-print	United states	2466	Community	56 ± 16	46	Fully vaccinated: 3.3% Partially vaccinated: 6.2% Not vaccinated: 2.9% Unknown: 88%	Majority Alpha, followed by Delta*	10 March 2021–25 June 2021	Bamlanivimab alone, bamlanivimab/etesevimab, or casirivimab/ imdevimab^**α**^
Dougan et al. 2021 (b)^17^ NCT04427501; BLAZE-1	Pre-print	United States	769	Community	NR	46.9	NR	NR	9 Dec 2020–7 Jan 2021	bamlanivimab 700 mg and etesevimab 1400 mg, or placebo IV
Gupta et al. 2021 (b)^18^ NCT04545060; COMET-ICE	Pre-print	United States, Canada, Brazil, Spain, Peru	1057	Community	NR	46	NR	NR	Aug 2020–March 2021	Sotrovimab 500 mg or placebo IV

NR, not reported; IV, intravenous.

µ, sample size of the efficacy populations reported. *, Variants of concern reported in Pennsylvania (the state where the study was located) during the study period (presented as a figure). ¥, peer-reviewed versions of previously included pre-prints—additional data has been extracted from the peer-reviewed versions. α, Dose provided as per FDA Emergency Use Authorisation guidance (dosing guidance changed over time).

**Table 2:** Outcome data extracted from articles.

Publication	Mortality	Hospitalisation (all cause)	Hospitalisation (COVID related)	Non-invasive ventilation	Invasive ventilation	ICU	Infusion related AEs	SAEs	Mean length of hospital stay (SD)
**Weinreich et al. 2021 (c) **^11^									
Cas/imd 1200 mg	1/736	7/736	6/736	–	1/736	3/736	2/827	9/827	7 (8.04)
Placebo	1/748	26/748	23/748	–	2/748	7/748	0/1843	74/1843	8.4 (6.74)
Cas/imd 2400 mg	1/1355	–	–	–	–	–	–	24/1849	–
Placebo	3/1341	–	–	–	–	–	0/1843	74/1843	–
**Weinreich et al. 2021 (cii) **^12^									
Cas/imd 2400 mg	–	20/1355	17/1355	–	1/1355	6/1355	1/1849	-	8.6 (7.07)
Placebo	–	66/1341	59/1341	–	6/1341	18/1341	–	-	10 (7.16)
**Dougan et al. 2021 **^13^									
Bam/ete 2800/2800 mg	0/518	–	11/518	–	–	–	–	7/518	7.3 (6.4)
Placebo	10/517	–	33/517	–	–	–	–	5/517	11.2 (10.1)
**Gupta et al. 2021 **^14^									
Sot 500 mg	0/291	3/291	–	0/291	0/291	0/291	–	7/430	–
Placebo	1/292	21/292	–	5/292	2/292	5/292	–	26/438	–
**Gupta et al. 2021 (c) **^15^									
Sot 500 mg	–	–	–	–	–	–	6/430	–	11(7)
Placebo	–	–	–	–	–	–	5/438	–	8.71 (7.13)
**McCreary et al. 2021 **^16^									
Bam*	1/128	16/128	–	–	–	–	0/128	0/128	–
Bam/ete*	7/885	130/885	–	–	–	–	12/885	1/885	–
Cas/imd*	6/922	132/922	–	–	–	–	9/922	4/922	–
**Dougan et al. 2021 (b) **^17^									
Bam/ete 700/1400 mg	0/511	4/511	–	–	–	–	–	6/511	–
Placebo	4/258	14/258	–	–	–	–	–	2/258	–
**Gupta et al. 2021 (b) **^18^									
Sot 500 mg	0/528	6/528	–	0/528	0/528	0/528	6/523	11/523	9.33 (5.16)
Placebo	2/529	29/529	–	10/529	4/529	10/529	6/526	32/526	11.36 (12.12)

Bam, bamlanivimab; Bam/ete, bamlanivimab/etesevimab; Sot, sotrovimab; Cas/imd, casirivimab/imdevimab; ICU, intensive care unit; AE, adverse event; SAE, serious adverse event; SD, standard deviation.

*FDA Emergency Use Authorisation dosing.

### Overview of the trials identified

Risk of bias assessment of the included randomised controlled trials is reported in Appendix 3. Among the trials one was low risk of bias, one was judged to be at “some concerns” for bias, and two were at high risk of bias.

One of the trials (COMET-ICE) investigated the efficacy of sotrovimab versus placebo^14,15,18^, one of the trials investigated casirivimab/imdevimab versus placebo^11^, another trial (BLAZE-1) investigated bamlanivimab/etesevimab versus placebo^13,17^ The final trial (OPTIMISE-C19) investigated bamlanivimab alone versus bamlanivimab/etesevimab or casirivimab/imdevimab^16^. In all studies, the proportion of female participants was higher than male. Mean age was reported for two of the studies (and was below 60 years in both)^13,16^. In general, the articles did not report the COVID-19 variants which participants were infected with. An article on one of the studies reported the vaccination status of participants^16^, two studies excluded vaccinated participants^11,12^, while the remaining study had a protocol amendment in January 2021 to allow inclusion of vaccinated participants (having initially excluded patients who had participated in a SARS-CoV-2 vaccine study)^13^.

### Meta-analysis

**Figure 2:**
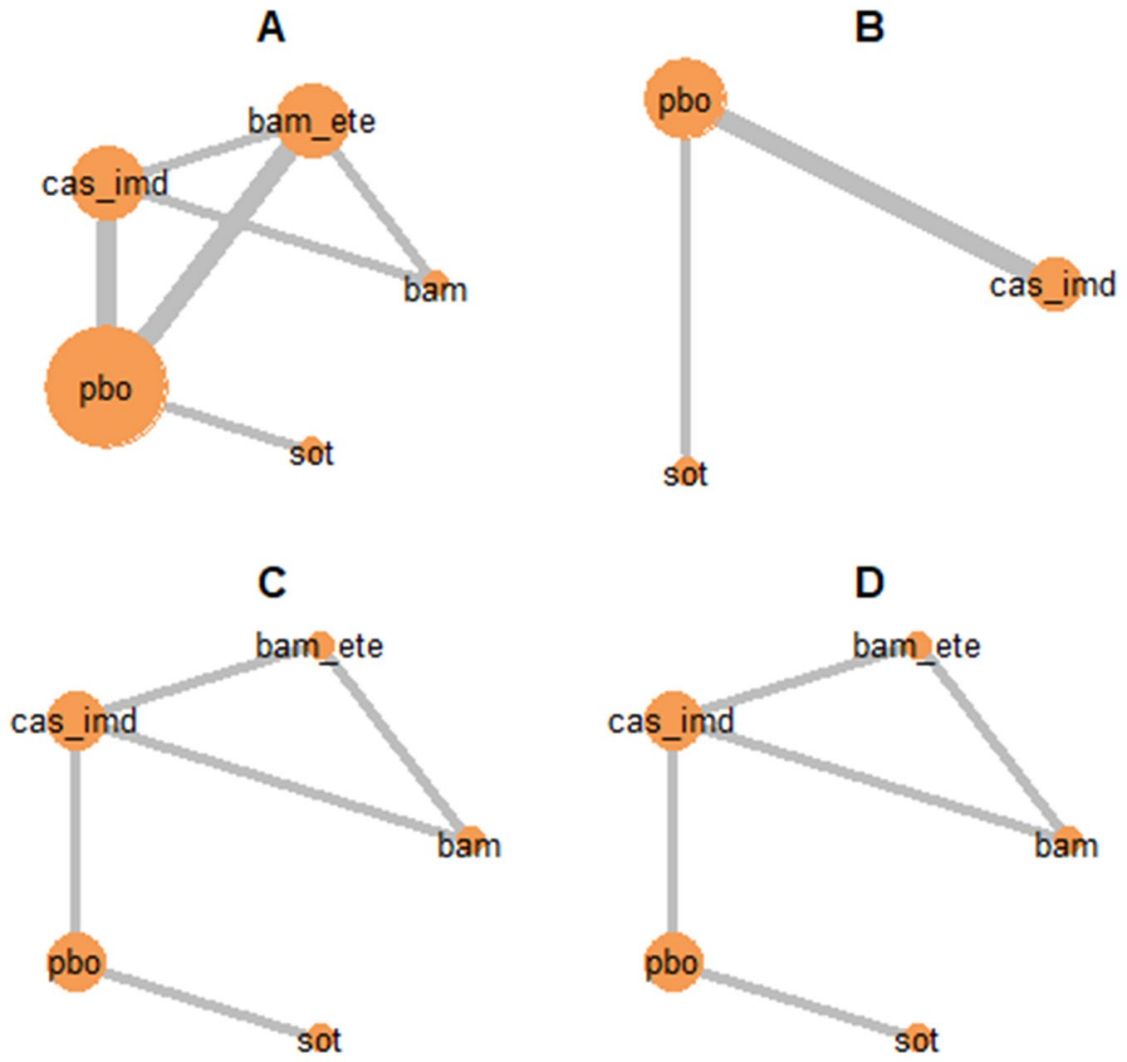
Network diagrams for the outcome-specific NMAs: mortality (**A**), hospitalisation (**A**), invasive ventilation (**B**), ICU admission (**B**), infusion-related AEs (**C**), and serious AEs (**D**). Notation: pbo, placebo; bam, bamlanivimab; bam_ete; bamlanivimab/etesevimab; cas_imd, casirivimab/imdevimab; sot, sotrovimab. A line joining two nodes indicates that there is RCT evidence comparing the corresponding treatments for the relevant outcome. The thickness of each line is proportional to the number of RCTs providing evidence on this comparison (in practice this is either one or two studies for each comparison).

**Figure 3:**
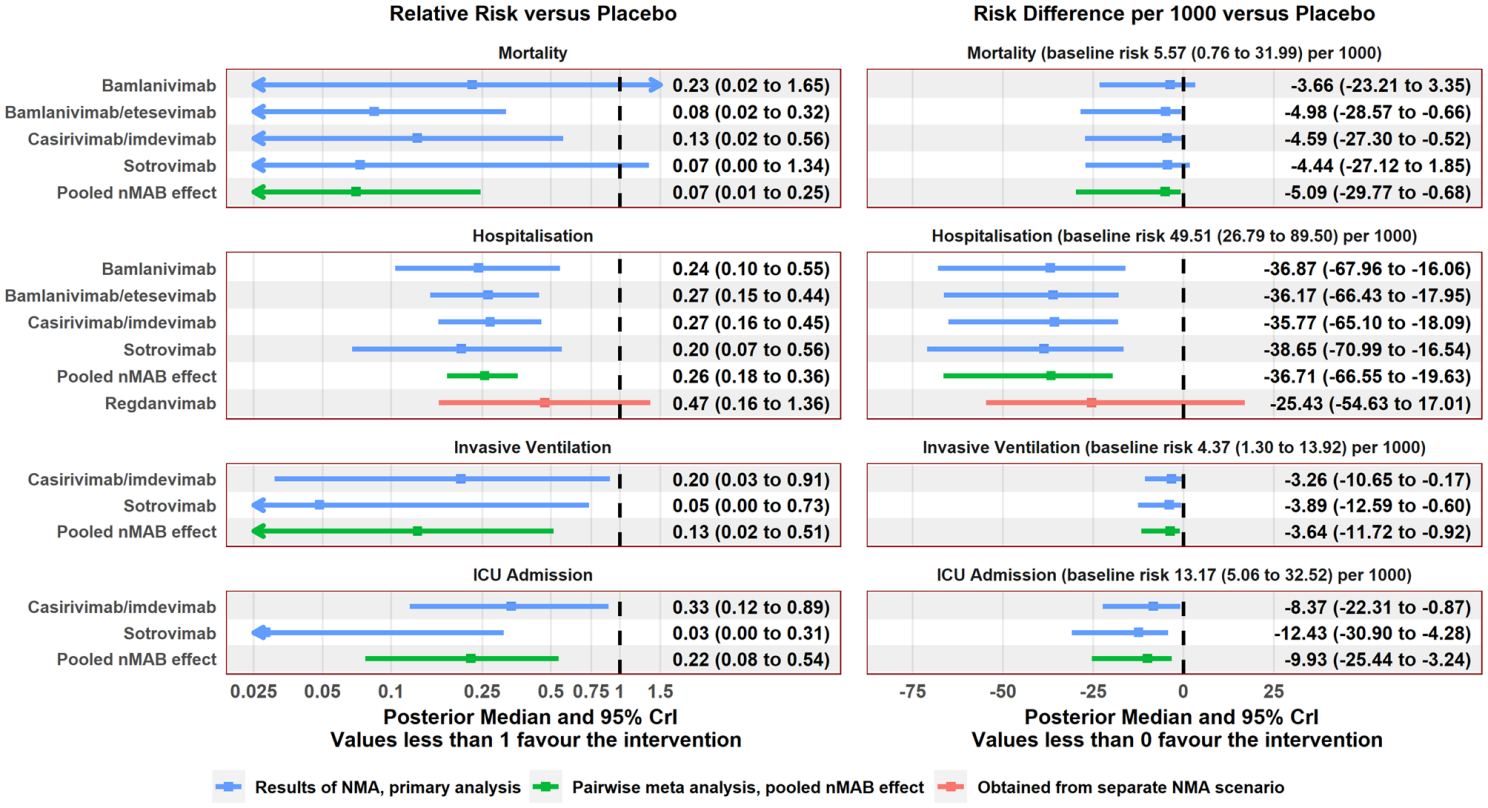
Summary of relative and absolute treatment effects for each treatment compared with SoC from the NMA, efficacy outcomes. Results obtained from Bayesian random-effects network meta-analysis model. Risk difference per 1000 patients represents the difference in number of events that would be expected to if 1000 patients with nMABs rather than SoC. Baseline risks for each event type were obtained by pooling event rates from the placebo arms across all included studies using a random-effects model. Treatment effects for regdanvimab were obtained from a separate scenario analysis. Regdanvimab is not included in the ‘pooled nMAB effect’ as no studies of regdanvimab meeting the inclusion criteria of the review were identified.

**Figure 4:**
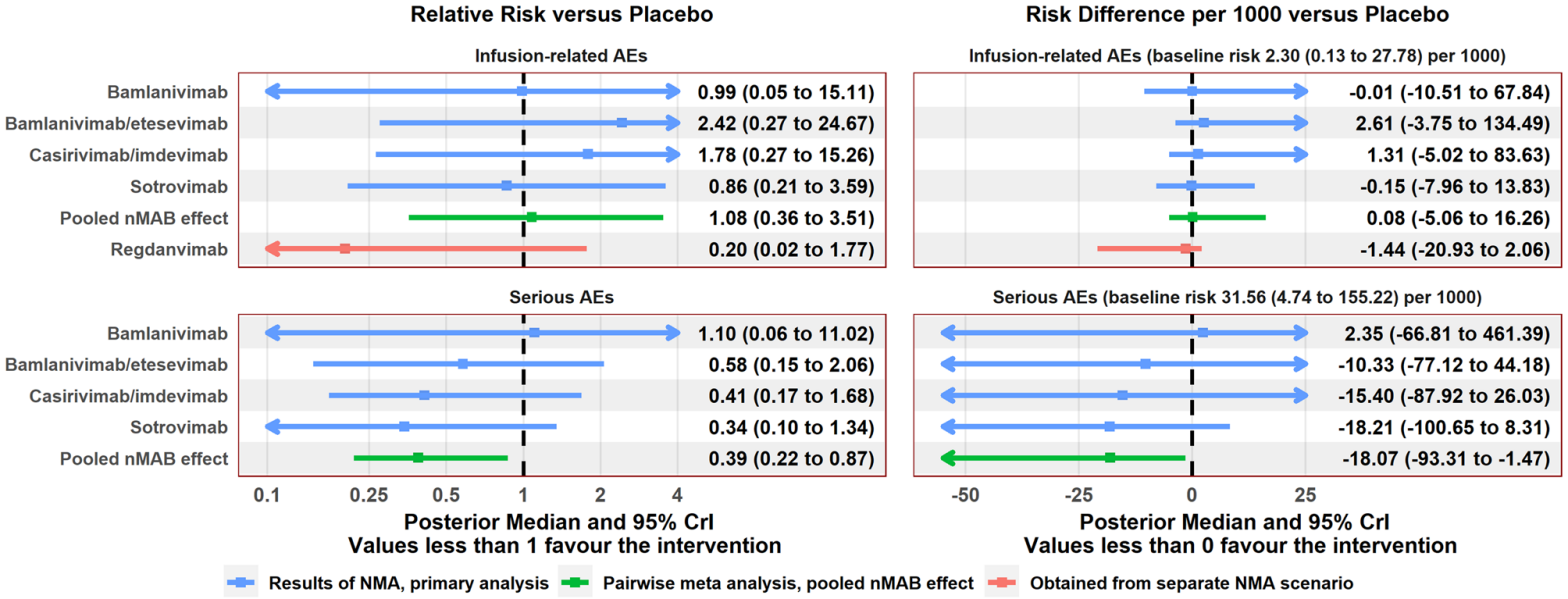
Summary of relative and absolute treatment effects for each treatment compared with SoC from the NMA, safety outcomes. Results obtained from Bayesian random-effects network meta-analysis model. Risk difference per 1000 patients represents the difference in number of events that would be expected to if 1000 patients with nMABs rather than SoC. Baseline risks for each event type were obtained by pooling event rates from the placebo arms across all included studies using a random-effects model. Treatment effects for regdanvimab were obtained from a separate scenario analysis. Regdanvimab is not included in the ‘pooled nMAB effect’ as no studies of regdanvimab meeting the inclusion criteria of the review were identified.

**Figure 5:**
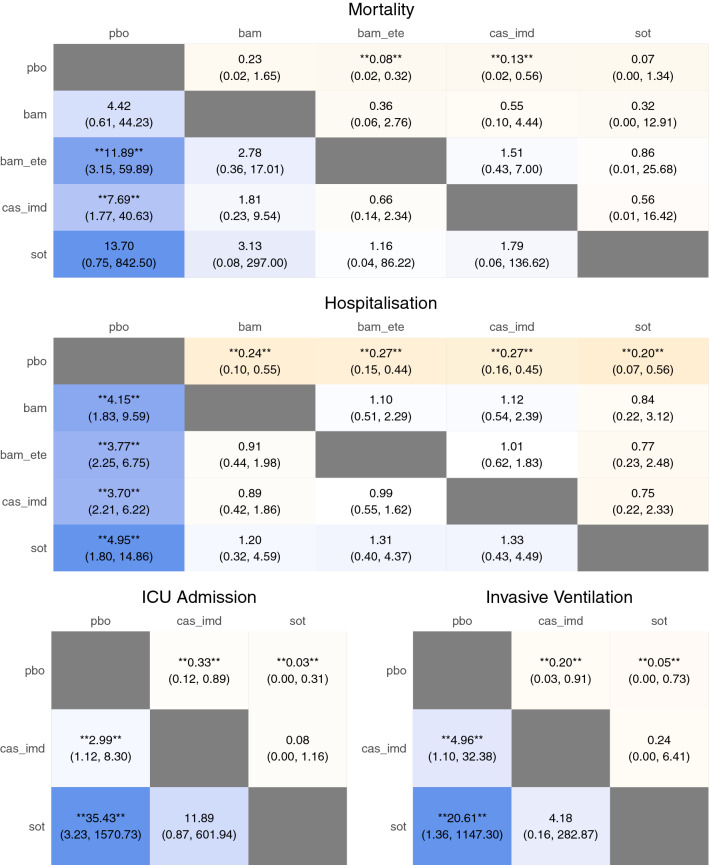
NMA results showing relative risks for all pairwise comparisons for efficacy outcomes (posterior median and 95% credible interval). Notation: pbo, placebo; bam, bamlanivimab; bam_ete; bamlanivimab/etesevimab; cas_imd, casirivimab/imdevimab; sot, sotrovimab. For each pairwise comparison, posterior median and 95% credible interval are shown for the estimated relative risk. Values of greater than one indicate that the given event is more frequent for the intervention (column name) than the comparator (row name). A double asterisk (**) in a given cell indicates that the corresponding comparison is statistically significant at the nominal 95% confidence level (i.e. without correction for multiple comparisons).

**Figure 6:**
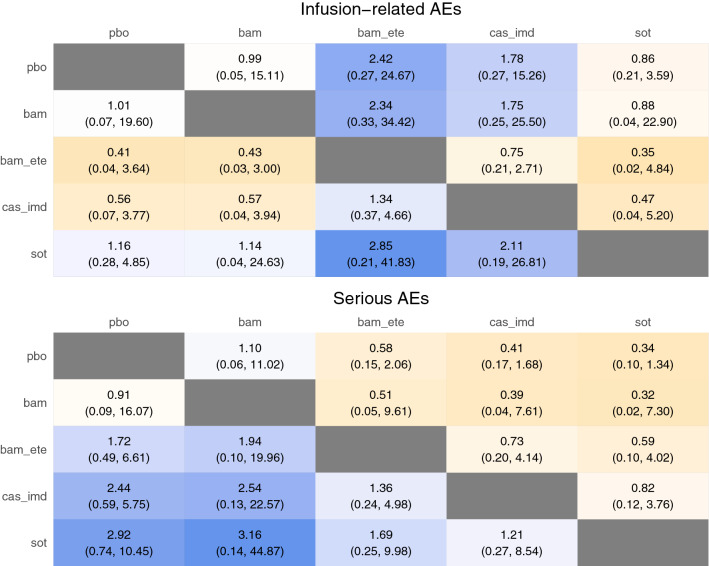
NMA results showing relative risks for all pairwise comparisons for safety outcomes (posterior median and 95% credible interval). Notation: pbo: placebo; bam, bamlanivimab; bam_ete; bamlanivimab/etesevimab; cas_imd, casirivimab/imdevimab; sot, sotrovimab. For each pairwise comparison, posterior median and 95% credible interval are shown for the estimated relative risk. Values of greater than one indicate that the given event is more frequent for the intervention (column name) than the comparator (row name). A double asterisk (**) in a given cell indicates that the corresponding comparison is statistically significant at the nominal 95% confidence level (i.e. without correction for multiple comparisons).

After assessment of the data extracted from the included papers, NMA was carried out for the (dichotomous) outcomes of mortality, hospitalisation, invasive ventilation, ICU admission, infusion-related AEs, and serious AEs. An NMA for the outcome of hospital length of stay was not carried out due to model complexity. Network diagrams are shown in Fig. 2. Estimated treatment effects for each nMAB versus placebo, as well as the pooled nMAB versus placebo effect, are shown in Fig. 3 (efficacy) and Fig. 4 (safety). Results of all pairwise comparisons between different nMABs are shown in Figs. 5 and 6; additional results including sensitivity analyses are presented in Appendix 4.

The results of the primary analysis (which excludes regdanvimab) show a statistically significant reduction in the risk of hospitalisation associated with all nMAB therapies versus placebo, with point estimates of this reduction in the range of approximately 70–80%. For other efficacy outcomes, confidence intervals for relative risks estimates were typically wide due to small event numbers. Reductions in the risks of ICU admission and progression to invasive ventilation for casirivimab/imdevimab and sotrovimab were significant but uncertain. Numerically lower mortality was observed for all nMAB therapies compared with placebo, and the estimated reductions were significant for bamlanivimab/etesevimab and casirivimab/imdevimab versus placebo, though again estimates were highly uncertain in all cases.

Considering the pairwise comparisons between different nMAB therapies, no relative risks were significantly different from 1 for any outcome. The pooled nMAB versus placebo effect was statistically significant for all efficacy outcomes.

For the safety outcomes, event numbers were low and there was no clear difference in event risks between any nMAB and placebo. The pooled nMAB effect indicated that overall, nMAB therapies may be associated with a lower incidence of serious AEs compared with placebo, which could potentially reflect a high proportion of serious AEs being COVID-related.

Baseline absolute event risks for SoC were estimated at approximately 5% for hospitalisation, 0.6% for mortality, 0.4% for invasive ventilation and 1.3% for ICU admission. It is estimated that treating 1,000 patients with nMABs would prevent approximately 37 hospitalisations, 10 ICU admissions including 4 invasive ventilation events, and 5 deaths, compared with placebo, though these estimates are subject to considerable uncertainty. For safety outcomes, the baseline risk of infusion related AEs was estimated at 0.2%, and for serious AEs 3.2%. Absolute risk differences per 1000 for safety outcomes were highly uncertain.

When regdanvimab was included as a comparator in a scenario analysis, it was associated with numerically fewer hospitalisations and infusion-related AEs compared with placebo, though the associated relative risks were not statistically significant in either case. The inclusion of regdanvimab had a negligible effect on other treatment effects in the NMAs. Other outcomes were not available for analysis in this scenario.

Sensitivity analysis showed that the NMA results were at most only moderately sensitive to the choice of prior distribution for the treatment effect parameters, with outcomes with fewer event numbers being more sensitive. Selecting fixed-effects models reduced the width of confidence intervals slightly but had no major impact on the results. The results were moderately sensitive to the choice of prior distribution for the heterogeneity parameter, as expected in light of the small number of included studies.

## Discussion

This paper outlines our attempts to synthesis the available evidence for nMABs in a formal Bayesian Network meta-analysis. The uncertainty associated with combining information from somewhat heterogeneous trials is accounted for by using the random-effects framework in the NMA. We address the challenges of sparse data, known to be particularly problematic in the presence of heterogeneity^5,6^, with the use of weakly informative priors. This allowed us to obtain more precise estimates of treatment effects than would otherwise be possible, subject to the mild assumption that extreme values of these effects and of between-study heterogeneity are unlikely. We also estimate an average ‘nMAB versus placebo’ effect by treating this class of therapies as similar (but not strictly equivalent) interventions. While there are uncertainties in assuming similarity of these therapies, particularly in light of known differences in their ability to neutralise viral variants, we still believe that this analysis provides useful information on the potential therapeutic value of this class of treatments and may be beneficial for decision makers. Finally, we estimated absolute risk differences per 1000 for each therapy by modelling the baseline risks pooled across studies and combining these with the NMA results.

Three of the four studies included in the analysis were determined to be either at high risk or “some concerns” for bias with only one of the four determined to be at low risk. Displaying a moderate or high risk of bias is the considerable limitation of the analysis, however it was decided to include all the available evidence given the emerging and rapidly evolving nature in the disease area. A key issue across the studies and outcomes which we have included in this review is the small number of events, which results in imprecise estimates of treatment effects (i.e. wide confidence intervals). In other words, there is a strong possibility that the true treatment effect differs considerably from the effect observed in the studies. A further consequence of this is that the absolute risk reductions are small and uncertain, particularly for the outcomes of mortality, invasive ventilation, and ICU admission, where estimates range between 3 and 12 events prevented per 1000 patients treated. Even when these reductions are statistically significant, their clinical relevance is unclear, particularly in light of the practical challenges of administering these therapies on a large scale. There were considerable differences between the study populations, settings, and characteristics, which means that any comparisons between the treatment effects observed in different trials must be treated with caution. In particular, there were differences in the distributions of COVID-19 risk factors across included studies, which may negatively impact upon the certainty of results. Moreover, the definitions of ‘high-risk’ patients differed between trials. For example, the minimum age at which patients were automatically deemed to be high risk and thus eligible for enrolment (in the absence of other comorbidities) was 50 in Weinreich et al.^12^, 55 in Gupta et al.^18^, and 65 in Dougan et al.^13,17^ and McCreary et al.^16^.

Two existing published reviews with a focus on COVID-19 neutralising antibodies were identified in the course of this review. A Cochrane review by Kreuzberger et al.^19^ was published in 2021. However, for that review no formal evidence synthesis (i.e. meta-analysis) was carried out as the review identified only one study per comparison (as a result of treating different doses as distinct interventions); network meta-analysis was not considered. While this decision is reasonable, we believe formally combining along with robust exploration of assumptions to be a potentially more useful approach for decision makers. We are not aware of other studies that have estimated an average ‘pooled nMAB versus placebo’ effect as we have done here. A living systematic review and meta-analysis of RCTs investigating antibody and cellular therapies for the treatment of COVID-19 was published by Siemieniuk et al.^20^ in 2021. While the inclusion criteria of the Siemieniuk et al. review were broader than those of the present review, the results are broadly similar. Siemieniuk et al.^20^ also carried out a Bayesian NMA, however relative effects were estimated as odds ratios (rather than relative risks) and non-informative prior distributions were used for treatment effects. While effect estimates for the outcome of hospitalisation are similar, those for all other outcomes are considerably less precise (i.e. have far wider confidence intervals) in the Siemieniuk et al.^20^ paper, compared with those presented here. This illustrates the potential advantage of using weakly-informative prior distributions for treatment effects, particularly for less common events. The estimation of absolute effects also differed between reviews: Siemieniuk et al.^20^ took the median event rate from the placebo arms of the included studies as the baseline event rate, while here we have modelled this rate by pooling these placebo arms using a random-effects model. As a result, estimated absolute risk differences for nMABs versus placebo differ between studies. We believe that our approach provides more reliable estimates of the absolute treatment effects (specifically, the ranges of plausible effects described by the credible intervals) as it captures the uncertainty in baseline event rates across studies.

The rapid emergence of variants of SARS-CoV-2 has posed a particular challenge for nMABs. As the clinical trials were undertaken at different times and in the presence of different circulating variants the extrapolation of data to populations affected by different variants is questionable. Since undertaking this review one nMAB has been withdrawn (bamlanivimab/etesevimab) by the manufacturer and others are suggested to have considerably less or no benefit against the omicron variant, now the predominant variant globally^21–28^. The further emergence of sub-variants of omicron has further called the benefit of these products into question^26^.

The impact of vaccination status is an important factor in determining the generalisability to the population to be treated. Many countries have significant vaccination coverage for SARs-CoV-2 and therefore the applicability of the benefit of a treatment in an unvaccinated population needs to be carefully considered. In general, the vaccination status of participants in the included trials was not reported, however, given the timeframes of the studies involved it is likely that most enrolled patients were unvaccinated. A particular consequence is that the absolute risk reductions estimated here may not be generalisable to a vaccinated population, due to the considerably lower baseline risks of adverse outcomes following vaccination. For example, if baseline hospitalisation risk were 10 events per 1000 vaccinated individuals, then the absolute risk reduction for any nMAB therapy could not exceed this value; in particular, the absolute reductions estimated in this review (ca. 35–40 events per 1000) could not possibly generalise to such a population. In general, when the baseline risk of harm is markedly lower the absolute benefit for a population will be considerably less. By contrast, there is no such restriction on the relative treatment effects, and it is primarily a clinical question whether or not these may generalise to a (largely) vaccinated population. Finally, we note that a key population of interest for nMAB treatment is those individuals who fail to mount a sufficient antibody response to vaccination due to immunocompromise. While these individuals may have a similar baseline risk to the high-risk unvaccinated cohorts included in these studies, it is an open question whether or not the treatment effects estimated here will generalise to these patients, since immunosuppressant medications or other underlying conditions could potentially also affect response to nMAB treatment.

## Conclusion

Neutralising MAB therapies are likely to result in a clinically meaningful reduction in hospitalisations among SARS-CoV-2-infected individuals at high risk of progression to severe COVID-19. It is likely that mortality, ICU admission and invasive ventilation rates are also reduced, though the magnitude of any such effect is unclear. Comparisons between different nMAB therapies indicated broadly similar efficacy overall. The generalisability of this evidence to clinical practice is limited by the emergence of antibody-resistant variants and mass vaccination, and none of the included studies examined effectiveness among vaccine ‘non-responders.’ Nonetheless, this work also provides insight into the potential effectiveness of future therapies in this class at preventing severe outcomes in high-risk patients, provided that these treatments do indeed neutralise the SARS-CoV-2 variants that are circulating within the population.

## Supplementary Information

Supplementary Information 1.

Supplementary Information 2.

## Data Availability

All data inputs used in this study have been obtained from the published literature and preprints, and are included in the article itself. Code for the NMA is available in Appendix 4, which can be used to fully reproduce the results presented here.
