# Supplementary material for: Comparative effectiveness of neutralising monoclonal antibodies in high risk COVID-19 patients: a Bayesian network meta-analysis
Source: Sci Rep. 2022 Oct 20;12:17561. doi: 10.1038/s41598-022-22431-6 (PMC9583057; doi:10.1038/s41598-022-22431-6)
Supplement: Supplementary file 1 — Supplementary Information 1. [file 41598_2022_22431_MOESM1_ESM.pdf]

Appendix 1: Protocol

Purpose

The purpose of this protocol is to outline the process that will be used to rapidly identify and synthesise evidence on the use neutralising monoclonal antibodies (nMABs) for the treatment of COVID-19.

Process

This review will utilise a rapid review methodology. The title and abstract screening, full text screening, and data extraction will be undertaken by a single member of the review team.

Searching

The following sources will be searched to identify evidence:

- Bibliographic databases – Embase and Pubmed
- EMA assessment reports for the nMABs currently under rolling review by CHMP (hand searched to identify relevant RCTs)
- COVID-19 treatment guidelines from the BMJ and WHO (hand searched to identify relevant RCTs)
- Bibliographies of included systematic literature reviews (hand searched to identify relevant RCTs)
- Medrxiv preprint server (preprints added on or after 1ST of April 2021 only)
- SSRN preprint server (preprints added on or after 1ST of April 2021 only)
- Research square (preprints added on or after 1ST of April 2021 only)

Screening

Citations identified through searching Embase and Pubmed will be imported into Endnote where duplicates will be removed. A purpose designed Excel worksheet will be used to screen citations. The titles and abstracts of all citations identified will be screened by a single reviewer using the below criteria.

Table 1 Inclusion criteria for the review

	Include
Population	Humans that have been exposed to/ or tested positive for COVID-19 who are at high risk (defined below) of developing severe COVID-19 disease.<br>High Risk: Patients at risk of severe disease due to age, BMI of 30kg/m^2 or more, or comorbidities such as hypertension, respiratory disease, or cardiovascular disease.<br>Populations of particular interest:<br>• Immunocompromised patients
Interventions	• Bamlanivimab / etesevimab (alone or in combination)<br>• Casirivimab / imdevimab (alone or in combination)<br>• Regdanvimab<br>• Sotrovimab
Comparators	No limits
Outcomes	Healthcare utilization outcomes - prevention of hospitalisation, duration of hospital stays, ICU rates<br>Disease severity outcomes – requirement for supplemental oxygen<br>Mortality<br>Adverse Events (AEs) (data on all AEs to be extracted and where where separately reported serious AEs and infusion-related AEs)

Study design	RCTs<br>SLRs of RCTs

All titles or abstracts which meet the population, intervention, and study type criteria will be selected for full text review (also undertaken by a single reviewer).

Data extraction

Data extraction will be undertaken by a single reviewer. A purpose designed data extraction form in Excel will be used.

The following data will be extracted:

- Study descriptors (trial names, authors, locations, sample size, setting, period when data was collected)
- Population – age, sex, comorbidities
- Epidemiological information - vaccination status, COVID variant
- Timing of treatment (relative to symptom onset or diagnosis)
- Disease severity
- Healthcare utilization outcomes - prevention of hospitalisation, duration of hospital stays, ICU rates
- Disease severity outcomes - requirement for supplemental oxygen
- Mortality
- Adverse Events (AEs)

Summarising findings

The review report will provide a descriptive overview of the identified papers. Depending on the evidence identified a synthesis of the evidence may be performed.

Appendix 2: Search strategies

Table 2 Pubmed search 27 September 2021

#	Query	Results
	Intervention	
1	(((Sotrovimab OR GSK-4182136 OR GSK4182136 OR VIR-7831 OR VIR7831 OR ‘VIR 7831’) OR (‘Regdanvimab’ OR ‘CT P59’ OR ‘CT-P59’ OR ‘CTP59’ OR TP59 OR ‘TP59’)) OR (casirivimab OR imdevimab OR 'casirivimab/imdevimab' OR 'casirivimab and imdevimab' OR 'casirivimab+imdevimab' OR 'regen cov' OR 'regn 10933' OR 'regn 10987' OR regn10933 OR regn10987 OR 'rg 6413' OR 'rg 6412' OR ronapreve)) OR (‘bamlanivimab and etesevimab’ OR bamlanivimab OR 'ly 3819253' OR 'ly-3819253' OR 'ly3819253' OR 'ly cov 555' OR 'ly-cov555' OR etesevimab OR 'ly cov016' OR lycov016 OR js016 OR 'js 016' OR 'ly3832479' OR 'ly 3832479' OR 'ly-3832479')	310
	Study type	
2	randomized controlled trial [pt]	545,845
3	controlled clinical trial [pt]	635,388
4	randomized [tiab]	581,801
5	placebo [tiab]	227,973
6	drug therapy [sh]	2,378,162
7	randomly [tiab]	367,309
8	trial [tiab]	671,457
9	groups [tiab]	2,281,764

10	#2 OR #3 OR #4 OR #5 OR #6 OR #7 OR #8 OR #9	5,200,772
11	animals [mh] NOT humans [mh]	4,890,469
12	#10 NOT #11	4,528,947
13	case report [tw]	347,535
14	Case Reports [pt]	2,212,763
15	Observational Study [pt]	109,328
16	letter [pt]	1,151,891
17	historical article [pt]	404,970
18	Editorial [pt]	580,910
19	Guideline [pt]	36,211
20	News [pt]	208,863
21	Practice Guideline [pt]	29,080
22	#13 OR #14 OR #15 OR #16 OR #17 OR #18 OR #19 OR #20 OR #21	4,507,906
23	#12 not #22	4,631,532
	Population	
24	"covid 19"[All Fields] OR "covid 19"[MeSH Terms] OR "covid 19 vaccines"[All Fields] OR "covid 19 vaccines"[MeSH Terms] OR "covid 19 serotherapy"[All Fields] OR "covid 19 serotherapy"[Supplementary Concept] OR "covid 19 nucleic acid testing"[All Fields] OR "covid 19 nucleic acid testing"[MeSH Terms] OR "covid 19 serological testing"[All Fields] OR "covid 19 serological testing"[MeSH Terms] OR "covid 19 testing"[All Fields] OR "covid 19 testing"[MeSH Terms] OR "sars cov 2"[All Fields] OR "sars cov 2"[MeSH Terms] OR "severe acute respiratory syndrome coronavirus 2"[All Fields] OR "ncov"[All Fields] OR "2019 ncov"[All Fields] OR (("coronavirus"[MeSH Terms] OR "coronavirus"[All Fields] OR "cov"[All Fields]) AND 2019/11/01:3000/12/31[Date - Publication])	181,025
25	#1 AND #23 AND #24	65

Table 3 Embase search 27 September 2021

#	Query	Results
	Intervention	
1	'casirivimab plus imdevimab'	57
2	'casirivimab/imdevimab'/exp OR 'casirivimab/imdevimab' OR 'casirivimab and imdevimab' OR 'casirivimab+imdevimab'/exp OR 'casirivimab+imdevimab' OR 'regen cov*' OR 'regn cov*' OR 'regn 10933'/exp OR 'regn 10933' OR ‘regn10987'/exp OR 'regn 10987' OR 'regn10933'/exp OR regn10933 OR regn10987'/exp OR regn10987 OR 'rg 6413' OR 'rg 6412' OR ronapreve	134
3	'regdanvimab'	13
4	'regdanvimab' OR 'ct p59' OR 'ct-p59' OR 'ctp59' OR 'in-006' OR 'in006' OR 'in 006' OR 'regkirona' OR 't p59' OR tp59 OR 'tp59'	30
5	'bamlanivimab’	172
6	'etesevimab'	71
7	'bamlanivimab and etesevimab' OR bamlanivimab OR 'ly 3819253' OR 'ly-3819253' OR 'ly3819253' OR 'ly cov 555' OR 'ly-cov555' OR 'cov 555' OR 'cov555' OR 'cov-555' OR etesevimab OR 'ly cov016' OR lycov016 OR	194

	'lycov016' OR 'js016' OR js016 OR 'js 016' OR 'ly3832479' OR ly3832479 OR 'ly 3832479'	
8	'sotrovimab'	18
9	sotrovimab OR xevudy OR 'gsk 4182136' OR gsk4182136 OR 'gsk4182136' OR vir7831 OR 'vir 7831'	19
10	#1 OR #2 OR #3 OR #4 OR #5 OR #6 OR #7 OR #8 OR #9	268
	Population	
11	'coronavirus disease 2019'	154,203
12	'covid 19':ab,ti OR coronavirus:ab,ti OR 'corona virus':ab,ti	173,937
13	'2019-ncov':ab,ti OR '2019 ncov':ab,ti	1,381
14	'severe acute respiratory syndrome coronavirus 2':ab,ti	15,673
15	'2019':ab,ti AND (new:ab,ti OR novel:ab,ti) AND coronavirus:ab,ti	11,623
16	'sars-cov-2' OR sarscov2 OR 'sars cov 2':ab,ti	63,645
17	#11 OR #12 OR #13 OR #14 OR #15 OR #16	199,062
18	#10 AND #17	237
	Study type	
19	'randomized controlled trial'/de	678,625
20	'controlled clinical study'/de	435,073
21	randomi*ed:ti,ab	989,455
22	placebo:ti,ab	330,261
23	'drug therapy'	5,035,689
24	randomly:ti,ab	486,298
25	trial:ti,ab	964,242
26	groups:ti,ab	3,165,554
27	#19 OR #20 OR #21 OR #22 OR #23 OR #24 OR #25 OR #26	8,652,604
28	'animal' NOT 'human'	4,725,753
29	#27 NOT #28	7,825,403
30	'case report':ti,ab	476,240
31	'conference review':it	13,168
32	'editorial':it	697,752
33	'letter':it	1,180,316
34	'note':it	858,219
35	#30 OR #31 OR #32 OR #33 OR #34	3,214,202
36	#29 NOT #35	7,158,370
37	#18 AND #36	139

Table 4 Searches of pre-print sites

Preprint site searched	Date searched	Terms used	Date limits and filters	Results
medRxiv.org	28/08/2021	Search of titles and abstracts: “Sotrovimab GSK-4182136 GSK4182136 VIR-7831 VIR7831" "Regdanvimab CT-P59 CTP59 TP59" "casirivimab imdevimab casirivimab/imdevimab regn10933 regn10987 ronapreve" "bamlanivimab etesevimab ly-3819253 ly3819253 ly-cov555 lycov016 js016 ly3832479 ly-3832479" (match any words)	filtered to returned papers published after 1/04/2021	16

		(match any words)		
SSRN	30/09/2021	Sotrovimab, Regdanvimab, casirivimab, imdevimab, bamlanivimab, and etesevimab	filtered to returned papers published after 1/04/2021	1
Research Square	30/09/2021	Sotrovimab OR Regdanvimab OR casirivimab OR imdevimab OR bamlanivimab OR etesevimab (search of both titles and abstracts)	filtered to returned papers published after 1/04/2021 also filtered to only return COVID-19 pre-prints	2

Table 5 Pubmed search 14 December 2021

#	Query	Results
	Intervention	
1	(((Sotrovimab OR GSK-4182136 OR GSK4182136 OR VIR-7831 OR VIR7831 OR ‘VIR 7831’) OR (‘Regdanvimab’ OR ‘CT P59’ OR ‘CT-P59’ OR ‘CTP59’ OR TP59 OR ‘TP59’)) OR (casirivimab OR imdevimab OR 'casirivimab/imdevimab' OR 'casirivimab and imdevimab' OR 'casirivimab+imdevimab' OR 'regen cov' OR 'regn 10933' OR 'regn 10987' OR regn10933 OR regn10987 OR 'rg 6413' OR 'rg 6412' OR ronapreve)) OR (‘bamlanivimab and etesevimab’ OR bamlanivimab OR 'ly 3819253' OR 'ly-3819253' OR 'ly3819253' OR 'ly cov 555' OR 'ly-cov555' OR etesevimab OR 'ly cov016' OR lycov016 OR js016 OR 'js 016' OR 'ly3832479' OR 'ly 3832479' OR 'ly-3832479')	353
	Study type	
2	randomized controlled trial [pt]	553,463
3	controlled clinical trial [pt]	643,146
4	randomized [tiab]	591,345
5	placebo [tiab]	230,241
6	drug therapy [sh]	2,409,945
7	randomly [tiab]	372,584
8	trial [tiab]	683,170
9	groups [tiab]	2,315,373
10	#2 OR #3 OR #4 OR #5 OR #6 OR #7 OR #8 OR #9	5,272,281
11	animals [mh] NOT humans [mh]	4,923,607
12	#10 NOT #11	4,592,704
13	case report [tw]	353,347
14	Case Reports [pt]	2,230,492
15	Observational Study [pt]	115,620
16	letter [pt]	1,161,097
17	historical article [pt]	406,329
18	Editorial [pt]	588,775

19	Guideline [pt]	36,460
20	News [pt]	210,310
21	Practice Guideline [pt]	29,312
22	#13 OR #14 OR #15 OR #16 OR #17 OR #18 OR #19 OR #20 OR #21	4,550,365
23	#12 not #22	4,024,418
	Population	
24	"covid 19"[All Fields] OR "covid 19"[MeSH Terms] OR "covid 19 vaccines"[All Fields] OR "covid 19 vaccines"[MeSH Terms] OR "covid 19 serotherapy"[All Fields] OR "covid 19 serotherapy"[Supplementary Concept] OR "covid 19 nucleic acid testing"[All Fields] OR "covid 19 nucleic acid testing"[MeSH Terms] OR "covid 19 serological testing"[All Fields] OR "covid 19 serological testing"[MeSH Terms] OR "covid 19 testing"[All Fields] OR "covid 19 testing"[MeSH Terms] OR "sars cov 2"[All Fields] OR "sars cov 2"[MeSH Terms] OR "severe acute respiratory syndrome coronavirus 2"[All Fields] OR "ncov"[All Fields] OR "2019 ncov"[All Fields] OR (("coronavirus"[MeSH Terms] OR "coronavirus"[All Fields] OR "cov"[All Fields]) AND 2019/11/01:3000/12/31[Date - Publication])	207,458
25	#1 AND #23 AND #24	81
26	#1 AND #23 AND #24 from 2021/9/27 - 2021/12/14	28

Table 6 Embase search 14 December 2021

#	Query	Results
	Intervention	
1	'casirivimab plus imdevimab'	57
2	'casirivimab/imdevimab'/exp OR 'casirivimab/imdevimab' OR 'casirivimab and imdevimab' OR 'casirivimab+imdevimab'/exp OR 'casirivimab+imdevimab' OR 'regen cov*' OR 'regn cov*' OR 'regn 10933'/exp OR 'regn 10933' OR ‘regn10987'/exp OR 'regn 10987' OR 'regn10933'/exp OR regn10933 OR regn10987'/exp OR regn10987 OR 'rg 6413' OR 'rg 6412' OR ronapreve	134
3	'regdanvimab'	13
4	'regdanvimab' OR 'ct p59' OR 'ct-p59' OR 'ctp59' OR 'in-006' OR 'in006' OR 'in 006' OR 'regkirona' OR 't p59' OR tp59 OR 'tp59'	30
5	'bamlanivimab’	172
6	'etesevimab'	71
7	'bamlanivimab and etesevimab' OR bamlanivimab OR 'ly 3819253' OR 'ly-3819253' OR 'ly3819253' OR 'ly cov 555' OR 'ly-cov555' OR 'cov 555' OR 'cov555' OR 'cov-555' OR etesevimab OR 'ly cov016' OR lycov016 OR 'lycov016' OR 'js016' OR js016 OR 'js 016' OR 'ly3832479' OR ly3832479 OR 'ly 3832479'	194
8	'sotrovimab'	18
9	sotrovimab OR xevudy OR 'gsk 4182136' OR gsk4182136 OR 'gsk4182136' OR vir7831 OR 'vir 7831'	19
10	#1 OR #2 OR #3 OR #4 OR #5 OR #6 OR #7 OR #8 OR #9	268
	Population	
11	'coronavirus disease 2019'	154,203
12	'covid 19':ab,ti OR coronavirus:ab,ti OR 'corona virus':ab,ti	173,937

13	'2019-ncov':ab,ti OR '2019 ncov':ab,ti	1,381
14	'severe acute respiratory syndrome coronavirus 2':ab,ti	15,673
15	'2019':ab,ti AND (new:ab,ti OR novel:ab,ti) AND coronavirus:ab,ti	11,623
16	'sars-cov-2' OR sarscov2 OR 'sars cov 2':ab,ti	63,645
17	#11 OR #12 OR #13 OR #14 OR #15 OR #16	199,062
18	#10 AND #17	237
Study type		
19	'randomized controlled trial'/de	678,625
20	'controlled clinical study'/de	435,073
21	randomi*ed:ti,ab	989,455
22	placebo:ti,ab	330,261
23	'drug therapy'	5,035,689
24	randomly:ti,ab	486,298
25	trial:ti,ab	964,242
26	groups:ti,ab	3,165,554
27	#19 OR #20 OR #21 OR #22 OR #23 OR #24 OR #25 OR #26	8,652,604
28	'animal' NOT 'human'	4,725,753
29	#27 NOT #28	7,825,403
30	'case report':ti,ab	476,240
31	'conference review':it	13,168
32	'editorial':it	697,752
33	'letter':it	1,180,316
34	'note':it	858,219
35	#30 OR #31 OR #32 OR #33 OR #34	3,214,202
36	#29 NOT #35	7,158,370
37	#18 AND #36	213
38	#18 AND #36 AND [27-9-2021]/sd NOT [15-12-2021]/sd	90

Table 7 Searches of pre-print sites

Preprint site searched	Date searched	Terms used	Date limits and filters	Results
medRxiv.org	14/12/2021	Search of titles and abstracts: “Sotrovimab GSK-4182136 GSK4182136 VIR-7831 VIR7831" "Regdanvimab CT-P59 CTP59 TP59" "casirivimab imdevimab casirivimab/imdevimab regn10933 regn10987 ronapreve" "bamlanivimab etesevimab ly-3819253 ly3819253 ly-cov555 lycov016 js016 ly3832479 ly-3832479" (match any words) (match any words)	filtered to returned papers published after 27 September 2021	11
SSRN	14/12/2021	Sotrovimab, Regdanvimab, casirivimab, imdevimab, bamlanivimab, and etesevimab	filtered to returned papers published after 27 September 2021	0
Research Square	14/12/2021	Sotrovimab OR Regdanvimab OR casirivimab OR imdevimab OR	filtered to returned	6

		bamlanivimab OR etesevimab (search of both titles and abstracts)	papers published after 27 September 2021 also filtered to only return COVID-19 pre-prints	

Appendix 3: Risk of bias assessment

Intention-to-treat	Unique ID	Study ID	Experimental	Comparator	Outcome	Weight	D1	D2	D3	D4	D5	Overall
	mABReviewMo	COMET-ICE	Sotrovimab	Placebo	All outcomes	1	+	+	+	+	+	+
	mABReviewMo	NCT04425629	casirivimab/imdevimab	placebo	All outcomes	1	+	-	-	+	+	-
	mABReviewMo	BLAZE-1	bamlanivimab/etesevima	placebo	All outcomes	1	+	!	+	+	+	!
	mABReviewMo	OPTIMISE-C19	bamlanivimab/etesevima	Casirivimab/imdevimab	All outcomes	1	!	-	-	+	+	-

+	Low risk
!	Some concerns
-	High risk

D1	Randomisation process
D2	Deviations from the intended interventions
D3	Missing outcome data
D4	Measurement of the outcome
D5	Selection of the reported result

Figure 1 Summary of Risk of Bias assessment
