# Supplementary material for: Comparative effectiveness of neutralising monoclonal antibodies in high risk COVID-19 patients: a Bayesian network meta-analysis
Source: Sci Rep. 2022 Oct 20;12:17561. doi: 10.1038/s41598-022-22431-6 (PMC9583057; doi:10.1038/s41598-022-22431-6)
Supplement: Supplementary file 2 — Supplementary Information 2. [file 41598_2022_22431_MOESM2_ESM.pdf]

Comparative effectiveness of neutralizing monoclonal antibodies in high risk COVID-19 patients: a Bayesian network-meta-analyses.

Supplementary Material: full details of NMA methodology, code, extracted data, results, and sensitivity analysis.

David McConnell[1,2], Marie Harte[1,2], Cathal Walsh[1,3], Desmond Murphy[4,5], Alistair Nichol[6,7], Michael Barry[1,2], Roisin Adams[1,2]

1 National Centre for Pharmacoeconomics, St James's Hospital, Dublin, Ireland
2 Department of Pharmacology and Therapeutics, Trinity College Dublin, Dublin, Ireland
3 Health Research Institute and MACSI, University of Limerick, Limerick, Ireland
4 Cork University Hospital, Cork, Ireland
5 University College Cork, Cork, Ireland
6 St Vincent's University Hospital, Dublin, Ireland
7 School of Medicine, University College Dublin, Dublin, Ireland

Correspondence: David McConnell, dmcconnell@stjames.ie

Table of Contents

1 Methods 4
1.1 Overview 4
1.2 Identification of Evidence Networks 4
1.3 Extraction of outcome data 5
1.4 Statistical analysis 5
1.5 Calculation of absolute effects 6
1.6 Sensitivity analysis 6

2 NMA code 7
2.1 Required packages 7
2.2 Data preparation 7
2.3 Base Case NMA 9
2.4 Pooled mAB pairwise meta-analysis 12
2.5 Estimation of absolute treatment effects 13
2.6 Sensitivity Analysis 16
2.6.1 Prior distributions for relative treatment effects. 16
2.6.2 Comparison of fixed and random effects. 18
2.6.3 Heterogeneity parameter prior sensitivity analysis 19
2.6.4 Investigation of consistency 21
3 Complete tabulation of study-level data extracted 23
3.1 Notes on the extracted data 23
3.2 Extracted data for NMA 24
4 Results 28
4.1 Mortality 28
4.2 Hospitalisation 31
4.3 Invasive Ventilation 33
4.4 ICU Admission 35
4.5 Infusion-related AEs 37
4.6 Serious AEs 39
4.7 Pooled mAB effects 41
4.8 Inclusion of regdanvimab as intervention 41
4.8.1 Hospitalisation 42
4.8.2 Infusion-related AEs 43
4.9 Absolute Risk Reductions 44
4.9.1 Mortality 44

4.9.2 Hospitalisation ... 45
4.9.3 Invasive Ventilation ... 46
4.9.4 ICU Admission ... 47
4.9.5 Infusion-related AEs ... 48
4.9.6 Serious AEs ... 49
5 Sensitivity Analysis ... 50
5.1 Treatment effects prior ... 50
5.1.1 Mortality ... 50
5.1.2 Hospitalisation ... 52
5.1.3 Invasive Ventilation ... 54
5.1.4 ICU Admission ... 56
5.1.5 Infusion-related AEs ... 58
5.1.6 Serious AEs ... 60
5.2 Fixed versus random effects ... 63
5.2.1 Mortality ... 63
5.2.2 Hospitalisation ... 64
5.2.3 Invasive Ventilation ... 65
5.2.4 ICU Admission ... 66
5.2.5 Infusion-related AEs ... 67
5.2.6 Serious AEs ... 68
5.3 Heterogeneity Prior ... 69
5.3.1 Mortality ... 69
5.3.2 Hospitalisation ... 71
5.3.3 Invasive Ventilation ... 74
5.3.4 ICU Admission ... 76
5.3.5 Infusion-related AEs ... 78
5.3.6 Serious AEs ... 81

5.4 Appendix: Investigation of consistency 84

5.5 Appendix: Model diagnostics 85

References 95

1 Methods

1.1 Overview

Where feasible, formal evidence synthesis in the form of network meta-analysis (NMA) will be carried out. All outcomes extracted as part of the rapid review will be synthesised where possible.

1.2 Identification of Evidence Networks

Since the included studies are expected to report different outcomes, separate outcome-specific evidence networks will be constructed. Studies enrolling outpatients infected with or exposed to Covid-19 will be considered, while studies enrolling patients already hospitalised as a result of Covid-19 will not be included for the following reasons:

- Patient characteristics are likely to differ considerably, thus there is a high risk of effect modification if trials involving hospitalised patients are synthesised with those enrolling only outpatients
- The timing of the interventions relative to symptom onset is likely to differ considerably (hospitalised patients will receive treatment much later on average)

Different doses of the same mAB therapy will be treated as a single intervention in the base case. This decision was made in advance due to the expectation that the number of included studies would be small, and that certain event types (e.d. deaths, ICU admissions) were expected to be rare. This approach increases statistical power at the potential cost of additional heterogeneity (the latter will be accounted for via the use of random-effects models where possible).

Subgroup analysis may be carried out for the following groups if data is available:

- Immunocompromised patients
- Seronegative patients

The feasibility of including each study in the relevant evidence network will be then be assessed according to the following criteria:

- Comparability of baseline patient characteristics and inclusion criteria across trials in the network
- Consistency in the definitions and reporting of outcomes
- For dichotomous outcomes: at least one event reported across treatment arms

1.3 Extraction of outcome data

All available outcome data for studies meeting the PICO criteria will be extracted (including subgroup data). Separate outcome data for each treatment arm will be extracted where available, which is expected to consist of:

- Dichotomous outcomes: number of events and sample size by treatment arm
- Continuous outcomes: mean and standard error by treatment arm. Where standard error is not reported, this will be estimated from published confidence intervals or similar.

It is not anticipated that any other types of data (e.g. time to event, count data) will arise. For publications reporting only summary measures of treatment effect, these will also be extracted, together with the associated measures of uncertainty (e.g. standard errors and/or confidence intervals) for comparison purposes.

1.4 Statistical analysis

The following treatment effects will be synthesised:

- Relative risks for dichotomous outcomes
- Mean differences and/or mean ratios for continuous outcomes (the preferred measure of treatment effect will be context-specific)

Bayesian network meta-analysis will be used to synthesise treatment effects (Dias et al., 2013a). For dichotomous outcomes, a GLM with a binomial likelihood and a log link function will be used (Warn et al., 2002), while for continuous outcomes a normal likelihood and an identity link will be used. It anticipated that for the base case, random-effects meta-analysis will be used, except where there is only a single trial contributing to each comparison and no closed loops.

It is anticipated that the NMAs will include a small number of studies and that event counts will be low within each study. Therefore, for dichotomous outcomes, a normal distribution with mean 0 and standard deviation 2.82 will be used as a prior distribution for the treatment effect parameters. This is the weakly informative prior distribution recommended in (Günhan et al., 2020) for meta analysis of few studies of rare events, which is symmetric about the null (i.e. neither benefit nor harm is regarded as more likely a priori) and has 95% of the density between 1/250 and 250 (i.e. a 95% prior belief that no relative risk exceeds 250).

Due to the small number of studies in the network, weakly informative priors will be used for the heterogeneity parameters (HN(0.5) for relative risks) as discussed in (Röver et al., 2021) and elsewhere. Sensitivity analysis will be carried out on prior distributions.

Analysis will be carried out in R, primarily using the package BUGSnet (Béliveau et al., 2019) (which implements MCMC sampling in JAGS). MCMC sampling wil be conducted using 100,000 iterations of which 50,000 are burn-in iterations, and 5,000 adaptation iterations, and 3 chains.

Convergence and model plausibility will be assessed by checking:

- Trace plots
- Density plots of the posterior distributions for treatment effect and heterogeneity parameters
- Convergence diagnostics such as Gelman-Rubin diagnostics, Geweke'statistic etc. output by the BUGSnet package

If the anticipated base case model exhibits poor convergence or implausible posterior distributions, MCMC parameters may be adjusted to improve convergence.

Heterogeneity will primarily be assessed qualitatively via comparisons of the characteristics of the included studies (patients, outcomes etc.). Where differences in patient characteristics exist across studies, the potential for effect-modification will be assessed by examining subgroup analysis if included in the identified studies.

Consistency will be assessed by comparing direct and indirect estimates of treatment effects.

Estimated treatment effects will also be compared with estimates of the between-trial standard deviation parameter tau (where random-effects models have been used). As the evidence networks are not anticipated to contain a large number of comparisons, formal statistical methods to assess inconsistency and heterogeneity will not be carried out.

1.5 Calculation of absolute effects

In the case of dichotomous outcomes, baseline natural history models will also be fitted to the event rates in the SoC arms of the included studies. Again a GLM with a binomial likelihood and log link function will be used, with a preference for random-effects models where possible. These will be used to estimate the absolute event rates and risk reductions versus SoC for each treatment and each outcome.

1.6 Sensitivity analysis

The following sensitivity analysis will be carried out:

1. Calculation of a 'pooled mAB versus SoC effect' for each outcome
2. Inclusion of data from (Eom et al., 2021) (regdanvimab versus placebo) in the relevant evidence networks, allowing consideration of regdanvimab as a comparator

3. For the relative-effects parameter, the following alternatives will be explored:

   – More informative prior N(0, [log(10)/1.96]^2)

   – Less informative prior N(0, [log(10000)/1.96]^2)

4. Where random effects models have been used in the base case, the following alternative priors will be used for the heterogeneity parameter:

   – informative priors from (Turner et al., 2012) [log-normal(-4.06,1.45^2) for mortality and log-normal(-3.02, 1.85^2) for other outcomes]

   – Non-informative prior U(0,5)

   – Alternative weakly informative prior Half-Cauchy(0.5) (Röver et al., 2021)

Fixed-effects models will also be fitted.

2 NMA code

2.1 Required packages

```
# Packages for data processing
library(readxl)
library(dplyr)
library(tidyr)
library(stringr)

# Package to run NMAs
library(BUGSnet)

# Packages to produce plots
library(ggplot2)
library(grid)
library(gridExtra)
```

2.2 Data preparation

Note: data can be found in the attached spreadsheet and is also presented here under 'Compete Tabulation of Study-level Inputs.'

```
## Create a data frame of outcomes for looping over
# This is used repeatedly for running the same analysis for each outcome

outcomes_full<-data.frame("Outcome"=c("Mortality",
                                 "Hospitalisation",
                                 "Invasive Ventilation",
                                 "ICU Admission",
                                 "Infusion-related AEs",
```

```
                                 "Serious AEs"),
                     # This is basically the 'short name' for each outcome
                     "model"=c("mort",
                               "hosp",
                               "inv",
                               "icu",
                               "iae",
                               "sae"
                               ),
                     # Name of the R object used to store the processed NMA i
nput data for each outcome
                     "data"=c("mort_data",
                            "hosp_data",
                            "inv_data",
                            "icu_data",
                            "iae_data",
                            "sae_data"),
                     # Specify fixed or random for base case
                     "type"=c("re",
                              "re",
                              "re",
                              "re",
                              "re",
                              "re")

            )

# Load data from spreadsheets

# Vector of sheet names to extract from excel

outcomes<-outcomes_full$model

## Loop over sheets (outcomes), shorten treatment names for convenient plotti
ng, and assign a data frame to each

for (i in outcomes)
{

  # Load the excel sheet

  tmp_df<-read_excel("data_short.xlsx",
                     sheet=i)


  # Replace treatment names with short names
  tmp_df$tx<-str_replace_all(tmp_df$treatment,
                                    c("Bamlanivimab_etesevimab"="bam_ete",
                                      "Bamlanivimab"="bam",
```

```
                                             "Casirivimab_imdevimab"="cas_imd",
                                             "Sotrovimab"="sot",
                                             "Regdanvimab"="reg",
                                             "Placebo"="pbo"))


  # Save a copy of the full data frame for later use (in scenarios etc)
  assign(paste0(i,"_df"),tmp_df)

  #Now remove the excluded studies and process the data for the base case NMA
s

  tmp_df<-filter(tmp_df,
                 Exclude != "Y" | is.na(Exclude)
                 )

  # Process the data for use in BUGSnet

  nma_data<-data.prep(tmp_df,
                      varname.s = "study",
                      varname.t="tx")

  assign(paste0(i,"_data"),
         nma_data)

  rm(tmp_df,nma_data)

}
```

2.3 Base Case NMA

```
## This code is simply a wrapper for the BUGSnet functions nma.model and nma.
run functions in the case of binary outcomes
## The main purpose is to fix the model type (GLM with binomial liklihood and
log link) and the default priors for treatment effects and heterogeneity
## Note that 'data' must prepped by data.prep BUGSnet function (which is also
returned by the nma_summary function above)

nma_binary<-function(data,
                     type="re",# 'fe' or 're'
                     eff_prior="dnorm(0,0.1260097)", # Note this is sigma = l
og(250)/1.96 as suggested by Guenhan et al 2018
                     het_prior="dnorm(0,4)T(0,)" # Half-normal(0.5) prior for
heterogeneity
                     )
{
  invisible(
  model<-nma.model(data=data,
```

```
                      outcome="events",
                      N="sampleSize",
                      reference="pbo",
                      family="binomial",
                      link="log",
                      effects=ifelse(type=="fe",
                               "fixed",
                               "random"
                               ),
                      prior.d=eff_prior,
                      prior.sigma = ifelse(type=="re",
                                           het_prior,
                                           "DEFAULT")
  )
  )
  invisible(
results<-nma.run(model,n.adapt = 5000, n.iter = 100000)
  )
return(results)

}

# This code loops over all outcomes and runs the base case NMAs
# Output (i.e. including simulations) are saved in .RDS files
# Note that even if the cache is invalidated, the NMAs will only be re-run if
the .RDS files are deleted
# If changing any base case options you need to delete these files!

for(i in seq_along(outcomes))
  {

  nma_file<-paste0("./output_data/",outcomes[i],"_NMA.RDS")

  if(!(file.exists(nma_file)))
    {
  saveRDS(nma_binary(get(outcomes_full$data[i]),
                  type=outcomes_full$type[i]),
          file=nma_file
          )

  }
}

## This is a wrapper for a couple of BUGSnet functions to extract NMA results
## It takes the ouput of nma.run as input and returns a list consisting of:
# $sucraplot, $heatplot, $forestplot

get_nma_results<-function(results,
                          Title="" # Outcome name, if needed
                          )
```

```
{
nma_results<-list()

sucra.out <- nma.rank(results, largerbetter=FALSE, sucra.palette= "Set1")

sucraplot<-sucra.out$sucraplot+labs(title=paste0("Ranking: ",Title))

nma_results$sucraplot<-sucraplot

# Heatplot with treatments ordered by estimated superiority
league.out <- nma.league(results,
                         central.tdcy="median",
                         #order = sucra.out$order,
                         digits=2)


heatplot<-league.out$heatplot#+labs(title=paste0("Pairwise comparisons (Relat
ive Risks)"))

nma_results$heatplot<-heatplot

# Extract the tables so that we can save these if needed
nma_results$table<-league.out$table
nma_results$longtable<-league.out$longtable

# Forest plot relative to placebo
forestplot<-nma.forest(results,
           central.tdcy="median",
           comparator = "pbo",
           x.trans = "log")#+labs(title=paste0("Relative Risks versus Placebo
"))

nma_results$forestplot<-forestplot
return(nma_results)
}

# Loops over the outcomes, load the NMA simulations/output, extract the resul
ts and save to an appropriate file

for(i in seq_along(outcomes))
{
  nma_file<-paste0("./output_data/",outcomes[i],"_NMA.RDS")
  nma_results_file<-paste0("./output_data/",outcomes[i],"_NMA_results.RDS")

  if(!file.exists(nma_results_file))
    {
    # Load NMA sims
    nma_outputs<-readRDS(nma_file)
```

```
    # Generate NMA results
    nma_results<-get_nma_results(nma_outputs,Title=outcomes_full$Outcome[i])
    # Save NMA results as file
    saveRDS(nma_results,nma_results_file)
    # Save tables for article plots
    saveRDS(list("table"=nma_results$table,
                 "longtable"=nma_results$longtable),
            file=paste0("./plotting_data/",outcomes[i],"_NMA_tables.RDS"))
    rm(nma_outputs,nma_results)
    gc()
  }
}
```

2.4 Pooled mAB pairwise meta-analysis

We estimated the pooled mAB versus placebo effect by reclassifying all mAB therapies as a single intervention and carrying out the corresponding pairwise meta-analysis as before.

```
# This loop creates the data frame for the meta-analysis of pooled mABs versu
s placebo
# Load data from spreadsheets
# Exclude Eom21 (does not meet criteria) and McCreary 2021 (as it only contri
butes to mAB-mAB comparisons)
# Replace each intervention name with 'mAB'


for (i in outcomes)
{
  tmp_df<-get(paste0(i,"_df")) %>%
    # Exclude study of regdanvimab, and McCreary 2021 which had no placebo ar
m
    filter(Exclude != "Y" | is.na (Exclude),
           study != "Mc Creary 2021")

  # Replace all mAB short names with 'mAB'

  tmp_df$tx<-str_replace_all(tmp_df$tx,
                                  c("bam_ete"="mAB",
                                    "bam"="mAB",
                                    "cas_imd"="mAB",
                                    "sot"="mAB"
                                    ))

  nma_data<-data.prep(tmp_df,
                      varname.s = "study",
                      varname.t="tx")

  assign(paste0(i,"_pooled_data"),
         nma_data)
```

```
  rm(tmp_df,nma_data)

}

# This code runs the pooled mABs meta-analysis for each outcome and saves the
results
# All model inputs are unchanged from the base case

for(i in seq_along(outcomes))
{
  nma_pooled_file<-paste0("./output_data/",outcomes[i],"_pooled_MA.RDS")

  if (!(file.exists(nma_pooled_file)))
    {
    saveRDS(nma_binary(get(paste0(outcomes[i],"_pooled_data")),
                       type=outcomes_full$type[i]),
            file=nma_pooled_file
    )

}
}
```

2.5 Estimation of absolute treatment effects

```
# This function generates an estimate of the baseline event risk pooled acros
s placebo arms of the included studies
# It takes as input the event numbers and sample sizes from the trials, using
the data that has been processed by BUGSnet for the main NMAs.
# These baseline risks are then pooled using a random effects logistic regres
sion model to estimate the posterior predictive distribution for baseline ris
ks
# The function returns a data frame of samples from this distribution

calculate_risk<-function(base_list)
{
  base_df<-base_list$arm.data %>%
  filter(tx=="pbo")

  # Create data frame for use in JAGS
base_model_df<-list(
  "ns"=nrow(base_df),
  "r"=base_df$events,
  "n"=base_df$sampleSize
  )
# JAGS code for baseline mode (adapted from NICE TSD5)
sink("model.txt")
cat("
# JAGS code below
```

```
# Binomial likelihood, logit link
# Baseline random effects model
model{ # *** PROGRAM STARTS
  for (i in 1:ns){ # LOOP THROUGH STUDIES
    r[i] ~ dbin(p[i],n[i]) # Likelihood
    logit(p[i]) <- mu[i] # Log-odds of response
    mu[i] ~ dnorm(m,tau.m) # Random effects model
  }
  mu.new ~ dnorm(m,tau.m) # predictive dist. (log-odds)
  m ~ dnorm(0,.0001) # vague prior for mean
  var.m <- 1/tau.m # between-trial variance
  tau.m <- pow(sd.m,-2) # between-trial precision = (1/between-trial variance
)
  sd.m ~ dnorm(0,4)T(0,) # weakly-informative prior for between-trial SD
  logit(R) <- m # posterior probability of response
  logit(R.new) <- mu.new # predictive probability of response
  logR.new<-log(R.new)
}
")
sink()

out <- jags.model(file="model.txt",
                  data=base_model_df,
                  n.adapt=5000,
                  n.chains = 3
            )

jagssamples <- coda.samples(out,
                            variable.names=c("R","R.new","logR.new","mu.new")
,
                            n.iter=100000,
                            thin=1)

jagssamples_df<-do.call(rbind.data.frame,jagssamples)
names(jagssamples_df)<-c("R","R.new","logR.new","logitR.new")

return(jagssamples_df)

}

# This function takes the output of a BUGSnet model run and extracts the post
erior simulations of the treatment effects

extract_effects<-function(nma_out)
{
  out_df<-do.call(rbind.data.frame,nma_out$samples) %>%
    select(1:length(nma_out$trt.key))
  names(out_df)<-nma_out$trt.key
  return(out_df)
```

```
}

# This functions takes as input the posterior simulations of the baseline ris
ks and treatment effects and generates estimated absolute risks, and risk dif
ferences versus placebo, for each treatment in the network

create_rd_samples<-function(n_iter=10000,baseline_risks,trt_effects)
{
  base_samples<-sample(baseline_risks$logR.new,n_iter)
  trt_samples<-sample_n(trt_effects,n_iter)
  abs_risks<-exp(base_samples+trt_samples)
  risk_diff<-exp(base_samples+trt_samples)-exp(base_samples)
  names(risk_diff)<-paste0(names(abs_risks),"_vs_pbo")
  return(list("abs"=abs_risks,
              "rd"=risk_diff))
}

for (i in outcomes)
{

  nma_sims<-readRDS(paste0("./output_data/",i,"_NMA.RDS"))

  pooled_ma_sims<-readRDS(paste0("./output_data/",i,"_pooled_MA.RDS"))



  effects<-cbind(extract_effects(nma_sims),
                 extract_effects(pooled_ma_sims)[-1]
                 )



 # Include regdanvimab effects if these have been calculated
  reg_sims_file<-paste0("./output_data/",i,"_NMA_reg.RDS")
  if(file.exists(reg_sims_file))
    {
    reg_sims<-readRDS(reg_sims_file)
    effects<-cbind(effects,
                   extract_effects(reg_sims)["reg"])
    }

  names(effects)<-str_replace_all(names(effects),c("mAB"="pooled_mABs","reg"=
"*reg"))


  invisible(
  samples<-create_rd_samples(n_iter=10000,
    calculate_risk(get(paste0(i,"_data"))),
```

```
    effects
  )
  )

  saveRDS(samples,
          file=paste0("./output_data/",i,"_ARR.RDS")
          )
  rm(samples,nma_sims)
  gc()

}
```

2.6 Sensitivity Analysis

2.6.1 Prior distributions for relative treatment effects.

For all outcomes we investigate the following alternative priors for the treatment effect parameters:

1. More informative prior: N(0,1.175^2). This distribution gives a 95% prior probability that no relative risk between any pair of treatments in the network exceeds 10 (note that 0.175=log(10)/1.96).

2. Less informative prior: N(0,4.700^2). This distribution gives a 95% prior probability that no relative risk between any pair of treatments in the network exceeds 10,000 (note that 4.700=log(10000)/1.96).

```
# This code runs sensitivity analysis on the prior distributions for the rela
tive treatment effects and saves the results.


eff_SA_file<-"./output_data/eff_sa.RDS"

# Scenarios: upper limits of 95% prior HDIs on the RR scale (base case=250)
eff_bounds<-c(10,250,10000)

# Calculate prior distribution parameters for passing to JAGS
  precisions<-(log(eff_bounds)/1.96)^{-2}
  eff_priors<-paste0("dnorm(0,",precisions,")")

# Prior distributions formatted for table captions
  printdists<-paste0("Efficacy prior  N(0,",format(precisions^{-1/2},digits=2
),"^2)",
                     c(" (more informative)",
                       " (base case)",
                       " (less informative)"
                       ))
```

```
  # Short versions for plot captions

  plotdists<- paste0("N(0,",format(precisions^{-1/2},digits=2),"^2)")

  # Vector of figure captions (note that we use grid.arrange so there is only
a single figure for each outcome)
  eff_SA_captions<-paste0("Sensitivity analysis using alternative prior distr
ibutions for the treatment effect parameter (log relative risk), for the outc
ome of ",
                          outcomes_full$Outcome,
                          ". Top: ",
                          printdists[1],
                          ", middle: ",
                          printdists[2],
                          ", bottom: ",
                          printdists[3])

if (!(file.exists(eff_SA_file)))
{
  effsaresults<-list()



# Loop over outcomes and run SA

  for (i in seq_along(outcomes))
  {
    plots=list()
    tables=list()
    effsaresults[[i]]<-list()
  for (j in seq_along(eff_priors))
    {

    ## Run NMA scenario: outcome i, effecicay prior j
    invisible(
      modelrun<-nma_binary(get(outcomes_full$data[i]),
                          type=outcomes_full$type[i],
                          eff_prior = eff_priors[j]
      ))

    # Save results as a list (nested within a list)

    effsaresults[[i]][[j]]<-list()
    effsaresults[[i]][[j]][["league"]]<-nma.league(modelrun,
                          central.tdcy="median",
                          digits=2)

    # Store the name of the distribution here
```

```
    effsaresults[[i]][[j]][["tablecaption"]]<-paste0(outcomes_full$Outcome[i]
,
                                                     ": ",
                                                     printdists[j])

    fplot<-nma.forest(modelrun,
           central.tdcy="median",
           comparator = "pbo")+
         labs(title=plotdists[j])

    effsaresults[[i]][[j]][["plot"]]<-fplot

  }
rm(modelrun)
gc()

}

  saveRDS(effsaresults,eff_SA_file)
  rm(effsaresults)
}
```

2.6.2 Comparison of fixed and random effects.

```
# Sensitivity analysis to compare fixed versus random effects.
# Run alternative NMA model (typically fixed effects, where base case is rand
om effects) and save NMA output
# Note that model is only run if output file does not exist already (delete t
o re-run)

for (i in seq_along(outcomes))
{
# Character string for alternative model type
  alt_type<-ifelse(outcomes_full$type[i]=="re","fe","re")

# File path to save NMA output
  alt_file<-paste0("./output_data/",outcomes[i],"_NMA_",alt_type,".RDS")

# Run only if the output file does not exist
  if (!(file.exists(alt_file)))
  {
    invisible(
    saveRDS(nma_binary(
        get(outcomes_full$data[i]),
        type=alt_type
      ),
      file=alt_file)
    )
```

```
  }

}

# Vector of  captions for the output figures

captions_fere<-paste0(c("Comparison of the fit of fixed and random effects mo
dels for the outcome of ",
                      "Comparison of estimated treatment effects (relative ri
sks) versus placebo for the outcome of "),
                      rep(outcomes_full$Outcome,times=1,each=2))
```

2.6.3 Heterogeneity parameter prior sensitivity analysis

For all outcomes we examined the sensitivity of the results to the choice of prior distribution for the heterogeneity parameter (between trial standard deviation). The following distributions were examined:

1. Base case: half-normal(0.5)
2. Uniform(0,5)
3. Informative priors from Turner et al. (2012) for the between trial *variance*: log-normal(-4.06,1.45^2) for mortality and log-normal(-3.02, 1.85^2) for other outcomes
4. Alternative weakly-informative prior: Half-Cauchy(0.5) Röver et al. (2021)

Turner et al. (2012) gives prior for sigma^2, therefore it is necessary to edit the JAGS code produced by BUGSnet to implement these in a scenario analysis. This is done in the following code chunk.

```
 hty_priors<-c("dnorm(0,4)T(0,)",
              "dunif(0,5)",
              "dlnorm(-3.02, 0.2921841)",
              "dt(0, pow(0.5,-2), 1)T(0,)"
              )
prior_names<-c("HN(0.5) (base case)",
               "Unif(0,5)",
               "from Turner et al.",
               "Half-Cauchy(0.5)"
               )

# Captions for the figures (note one plot per outcome again)
hty_prior_captions<-paste0("Estimated treatment effects for each mAB versus p
lacebo obtained from varying the prior distribution on the heterogeneity para
meter sigma, for the outcome of ",
                           outcomes_full$Outcome)

#het_sa_file<-"./output_data/het_prior_sa.RDS"
```

```
for (i in seq_along(outcomes))
{
  het_sa_file<-paste0("./output_data/het_prior_sa_",outcomes[i],".RDS")
  if (!file.exists(het_sa_file))
  {
  hetsaresults<-list()

  hetsaresults[[i]]<-list()

  # Temporarily change Turner et al prior if outcome is mortality

  hty_priors[3]<- ifelse(outcomes[i]=="mort",
                         "dlnorm(-4.06,0.4756243)",
                         "dlnorm(-3.02, 0.2921841)"
                         )

  for (j in seq_along(hty_priors))
  {
    # If we are not using Turner et al priors, can proceed as normal specifyi
ng priors for the standard deviation
    if(j!=3){
    invisible(
    modelrun<-nma_binary(get(outcomes_full$data[i]),
                         type="re",
                         het_prior=hty_priors[j]
    ))
    }

    # Hack to specify prior between-trial variance rather than standard devia
tion in BUGSnet when Turner et al is used
    if (j==3)
    {
      sca_model<-nma.model(data=get(outcomes_full$data[i]),
                      outcome="events",
                      N="sampleSize",
                      reference="pbo",
                      family="binomial",
                      link="log",
                      effects="random",
                      prior.d="dnorm(0,0.1260097)",
                      prior.sigma = hty_priors[j])

      # Edit the JAGS code created by nma.model
      sca_model$bugs<-str_replace(sca_model$bugs,
                                  "sigma ~ dlnorm",
                                  "sigma2 ~ dlnorm"
                                  )
```

```
      sca_model$bugs<-str_replace(sca_model$bugs,
                                  "sigma2 <- sigma[ ^]2",
                                  "sigma <- pow(sigma2,0.5)")
       invisible(
         modelrun<-nma.run(sca_model,n.adapt = 5000, n.iter = 100000)
         )
       rm(sca_model)


    }

   hetsaresults[[i]][[j]]<-list()


   leagueplot<-nma.league(modelrun,
                          central.tdcy="median",
                          digits=2)


   # Delete the plot environment to save memory
   leagueplot$plot_env<-rlang::new_environment()

   hetsaresults[[i]][[j]][["league"]]<-leagueplot

   hetsaresults[[i]][[j]][["tablecaption"]]<-prior_names[j]

   fplot<-nma.forest(modelrun,
           central.tdcy="median",
           comparator = "pbo")+
     labs(title=prior_names[j])

   fplot$plot_env<-rlang::new_environment()

   hetsaresults[[i]][[j]][["plot"]]<-fplot

  }
  saveRDS(hetsaresults,file=het_sa_file)
  rm(modelrun,hetsaresults)
  gc()
}


}
```

2.6.4 Investigation of consistency

The only closed loops in the evidence networks arise from the direct comparison between bamlanivimab/etesevimab and casirivimab/imdevimab carried out in (McCreary et al., 2021).

The outcomes affected are mortality, hospitalisation, infusion-related AEs and serious AEs. Consistency was assessed by comparing the direct estimate of relative effects of ballanivimab/etesevimab versus casirivimab/imdevimab from (McCreary et al., 2021), and the indirect estimate of the same effect obtained re-running an NMA of all studies evaluating bamlanivimab/etesevimab and casirivimab/imdevimab with (McCreary et al., 2021) excluded (note that the latter does not contribute to the indirect estimate as there is no placebo arm).

```
# This code carries out the investigation of consistency for the relevant out
comes
outcomes_c<-c("mort","hosp","sae")

filename_consistency<-"./output_data/check_consistency.RDS"


if(!(file.exists(filename_consistency)))
{
  consistency<-list()
  for (i in outcomes_c)
  {

   # Get only those studies producing an indirect estimate of the effect of b
amlanivimab/etesevimab versus casirivimab/imdevimab

   tmp_df<-get(paste0(i,"_df"))

   tmp_df_indirect<-filter(tmp_df,
                  Exclude != "Y" | is.na(Exclude),
                  study != "McCreary 2021" &
                  study != "Gupta 2021" &
                  tx %in% c("bam_ete","cas_imd","pbo"))


   nma_data_indirect<-data.prep(tmp_df_indirect,
                       varname.s = "study",
                       varname.t="tx")

  invisible(
    modelrun_indirect<-nma_binary(nma_data_indirect,type="fe")
    )

   consistency[[paste0(i,"_indirect")]]<-nma.league(modelrun_indirect,
                        central.tdcy="median",
                        digits=2)$longtable

  rm(tmp_df_indirect,nma_data_indirect,modelrun_indirect)

    tmp_df_direct<-filter(tmp_df,
                        study=="McCreary 2021",
```

```
                        tx!="bam")

    nma_data_direct<-data.prep(tmp_df_direct,
                        varname.s = "study",
                        varname.t="tx")

    invisible(
    model_direct<-nma.model(data=nma_data_direct,
                        outcome="events",
                        N="sampleSize",
                        reference="bam_ete",
                        family="binomial",
                        link="log",
                        effects="fixed"
                        )
    )
    invisible(modelrun_direct<-nma.run(model_direct,n.adapt = 5000, n.iter =
100000)
    )

    consistency[[paste0(i,"_direct")]]<-nma.league(modelrun_direct,
                            central.tdcy="median",
                            digits=2)$longtable

  rm(tmp_df,nma_data_direct,modelrun_direct)
}
  saveRDS(consistency,filename_consistency)
  }
```

3 Complete tabulation of study-level data extracted.

3.1 Notes on the extracted data

Treatments:

- **bam** bamlanivimab
- **bam_ete** bamlanivimab/etesevimab
- **cas_imd** casirivimab/imdevimab
- **pbo** placebo
- **reg** regdanvimab (note: included in scenario analyses only)
- **sot** sotrovimab

Studies:

- Dougan 2021: (Dougan et al., 2021b)
- Dougan 2021b: (Dougan et al., 2021a)
- Eom 2021: (Eom et al., 2021)
- Gupta 2021: (Gupta et al., 2021)
- McCreary 2021: (McCreary et al., 2021)
- Weinreich 2021, Weinreich 2021a, Weinreich 2021b: all refer to (Weinreich et al., 2021), see below

1. (Weinreich et al., 2021) consisted of two stages, investigating casirivimab 1200mg /imdevimab 1200mg vs placebo, and casirivimab 2800mg/imdevimab 2800mg versus placebo. Each stage has a separate concurrent placebo arm, and results were reported for each stage. It is therefore treated as two separate studies in the NMA for efficacy outcomes. However, for safety outcomes, only pooled placebo event numbers are reported - for this reason, pooled casirivimab/imdevimab event numbers are also used for these outcomes, treating Weinreich 2021 as a single study.
2. (Scenario analysis only) (Eom et al., 2021) contained three parallel arms, regdanvimab 40mk/kg, regdanvimab 80mg/kg, and placebo. Event numbers in the regdanvimab arms have been pooled in the NMA scenario where this study is included.
3. For the 'Hospitalisation' outcome, all studies reported all-cause hospitalisation except (Dougan et al., 2021b), which only included COVID-related hospitalisation.
4. Hospital Length-of-Stay was reported in three publications. Due to time-constraints and model complexity (i.e. the need to account for zero-inflated data) an NMA for this outcome has not been carried out.
5. For the supplemental oxygen outcome, only one study (Gupta et al., 2021) reported results in the base case ((Eom et al., 2021) also reported results but this study did not meet the inclusion criteria of the Review). This outcome has not been included in the analysis.

3.2 Extracted data for NMA

Table 2.1: Mortality, extracted data

study	treatment	dose	events	sampleSize	Notes	Outcome
McCreary 2021	Bamlanivimab	NR	1	128	NA	Mortality
McCreary 2021	Bamlanivimab_etesevimab	NR	7	885	NA	Mortality
McCreary 2021	Casirivimab_imdevimab	NR	6	922	NA	Mortality

study	treatment	dose	events	sampleSize	Notes	Outcome
Dougan 2021	Bamlanivimab_etesevimab	2800mg_2800mg	0	518	NA	Mortality
Dougan 2021	Placebo	PBO	10	517	NA	Mortality
Weinreich 2021a	Casirivimab_imdevimab	1200mg	1	736	NA	Mortality
Weinreich 2021a	Placebo	PBO	1	748	NA	Mortality
Weinreich 2021b	Casirivimab_imdevimab	2400mg	1	1355	NA	Mortality
Weinreich 2021b	Placebo	PBO	3	1341	NA	Mortality
Gupta 2021	Sotrovimab	500mg	0	528	NA	Mortality
Gupta 2021	Placebo	PBO	2	529	NA	Mortality
Dougan 2021b	Bamlanivimab_etesevimab	700mg_1400mg	0	511	NA	Mortality
Dougan 2021b	Placebo	PBO	4	258	NA	Mortality
Eom 2021	Regdanvimab	all	0	103	Excluded from analysis as no events observed	Mortality
Eom 2021	Placebo	PBO	0	103	Excluded from analysis as no events observed	Mortality

Table 2.2: Hospitalisation, extracted data

study	treatment	dose	events	sampleSize	Notes	Outcome
McCreary 2021	Bamlanivimab	NR	16	128	NA	All cause hospitalisation
McCreary 2021	Bamlanivimab_etesevimab	NR	130	885	NA	All cause hospitalisation
McCreary 2021	Casirivimab_imdevimab	NR	132	922	NA	All cause hospitalisation
Dougan 2021	Bamlanivimab_etesevimab	2800mg_2800mg	11	518	NA	Covid-related hospitalisation only
Dougan 2021	Placebo	PBO	33	517	NA	Covid-related hospitalisation only

study	treatment	dose	events	sampleSize	Notes	Outcome
Weinreich 2021a	Casirivimab_imdevimab	1200mg	7	736	NA	All-cause hospitalisation
Weinreich 2021a	Placebo	PBO	26	748	NA	All-cause hospitalisation
Weinreich 2021b	Casirivimab_imdevimab	2400mg	20	1355	NA	All-cause hospitalisation
Weinreich 2021b	Placebo	PBO	66	1341	NA	All-cause hospitalisation
Gupta 2021	Sotrovimab	500mg	6	528	NA	All-cause hospitalisation
Gupta 2021	Placebo	PBO	29	529	NA	All-cause hospitalisation
Eom 2021	Placebo	PBO	9	103	NA	All cause hospitalisation
Eom 2021	Regdanvimab	all	9	204	NA	All cause hospitalisation
Dougan 2021b	Bamlanivimab_etesevimab	700mg_1400mg	4	511	Event numbers reported in Kaplan Meier plot only	Covid-related hospitalisation only
Dougan 2021b	Placebo	PBO	14	258	Event numbers reported in Kaplan Meier plot only	Covid-related hospitalisation only

Table 2.3: Invasive Ventilation, extracted data

study	treatment	dose	events	sampleSize	Notes	Outcome
Weinreich 2021a	Casirivimab_imdevimab	1200mg	1	736	NA	Invasive ventilation
Weinreich 2021a	Placebo	PBO	2	748	NA	Invasive ventilation
Weinreich 2021b	Casirivimab_imdevimab	2400mg	1	1355	NA	Invasive ventilation
Weinreich 2021b	Placebo	PBO	6	1341	NA	Invasive ventilation
Gupta 2021	Sotrovimab	500mg	0	528	NA	Mechanical ventilation or ECMO

study	treatment	dose	events	sampleSize	Notes	Outcome
Gupta 2021	Placebo	PBO	4	529	NA	Mechanical ventilation or ECMO
Eom 2021	Placebo	PBO	0	103	NA	Invasive ventilation
Eom 2021	Regdanvimab	all	1	204	NA	Invasive ventilation

Table 2.4: ICU Admission, extracted data

study	treatment	dose	events	sampleSize	Notes	Outcome
Weinreich 2021a	Casirivimab_imdevimab	1200mg	3	736	NA	ICU Admission
Weinreich 2021a	Placebo	PBO	7	748	NA	ICU Admission
Weinreich 2021b	Casirivimab_imdevimab	2400mg	6	1355	NA	ICU Admission
Weinreich 2021b	Placebo	PBO	18	1341	NA	ICU Admission
Gupta 2021	Sotrovimab	500mg	0	528	NA	ICU Admission
Gupta 2021	Placebo	PBO	10	529	NA	ICU Admission
Eom 2021	Regdanvimab	40mg_kg	0	101	NA	ICU Admission
Eom 2021	Regdanvimab	80mg_kg	0	103	NA	ICU Admission
Eom 2021	Placebo	PBO	0	103	NA	ICU Admission

Table 2.5: Infusion-related AEs, extracted data

study	treatment	dose	events	sampleSize	Notes	Outcome
McCreary 2021	Bamlanivimab	NR	0	128	NA	NA
McCreary 2021	Bamlanivimab_etesevimab	NR	12	885	NA	NA
McCreary 2021	Casirivimab_imdevimab	NR	9	922	NA	NA
Weinreich 2021	Placebo	PBO	0	1843	NA	Grade 2+ within 4 days. Concurrent placebo not reported.
Weinreich 2021	Casirivimab_imdevimab	all	3	2676	NA	Grade 2+ within 4 days. Concurrent placebo not reported.
Gupta 2021b	Sotrovimab	500mg	6	523	NA	Infusion reactions
Gupta 2021b	Placebo	PBO	6	526	NA	Infusion reactions
Eom 2021	Placebo	PBO	2	110	NA	NA
Eom 2021	Regdanvimab	all	1	215	NA	NA

Table 2.6: Serious AEs, extracted data

study	treatment	dose	events	sampleSize	Notes	Outcome
McCreary 2021	Bamlanivimab	NR	0	128	NA	NA
McCreary 2021	Bamlanivimab_etesevimab	NR	1	885	NA	NA
McCreary 2021	Casirivimab_imdevimab	NR	4	922	NA	NA
Dougan 2021	Bamlanivimab_etesevimab	2800mg_2800mg	7	518	NA	NA
Dougan 2021	Placebo	PBO	5	517	NA	NA
Weinreich 2021	Placebo	PBO	74	1843	Concurrent placebo arm results not reported.	NA
Weinreich 2021	Casirivimab_imdevimab	all	33	2676	Concurrent placebo arm results not reported.	NA
Gupta 2021	Sotrovimab	500mg	11	523	NA	NA
Gupta 2021	Placebo	PBO	32	526	NA	NA
Dougan 2021b	Bamlanivimab_etesevimab	700mg_1400mg	6	511	NA	NA
Dougan 2021b	Bamlanivimab_etesevimab	PBO	2	258	NA	NA
Eom 2021	Placebo	PBO	0	110	Excluded as no events	NA
Eom 2021	Regdanvimab	all	0	215	Excluded as no events	NA

4 Results

4.1 Mortality

Summary of Evidence Network

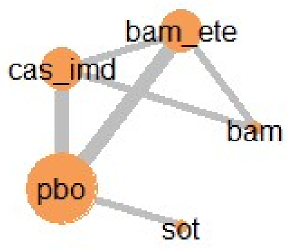

Figure 4.1: Network diagram, Mortality

Table 4.1: Summary of evidence network, Mortality

Characteristic	Value
Number of Interventions	5
Number of Studies	6
Total Number of Patients in Network	8976
Total Possible Pairwise Comparisons	10
Total Number of Pairwise Comparisons With Direct Data	6
Is the network connected?	TRUE
Number of Two-arm Studies	5
Number of Multi-Arms Studies	1
Total Number of Events in Network	36
Number of Studies With No Zero Events	3
Number of Studies With At Least One Zero Event	3
Number of Studies with All Zero Events	0

Table 4.1: Summary of comparison data, Mortality

comparison	n.studies	n.patients	n.outcomes	proportion
bam vs. bam_ete	1	1013	8	0.0078973
bam vs. cas_imd	1	1050	7	0.0066667
bam_ete vs. cas_imd	1	1807	13	0.0071942
bam_ete vs. pbo	2	1804	14	0.0077605

comparison	n.studies	n.patients	n.outcomes	proportion
cas_imd vs. pbo	2	4180	6	0.0014354
pbo vs. sot	1	1057	2	0.0018921

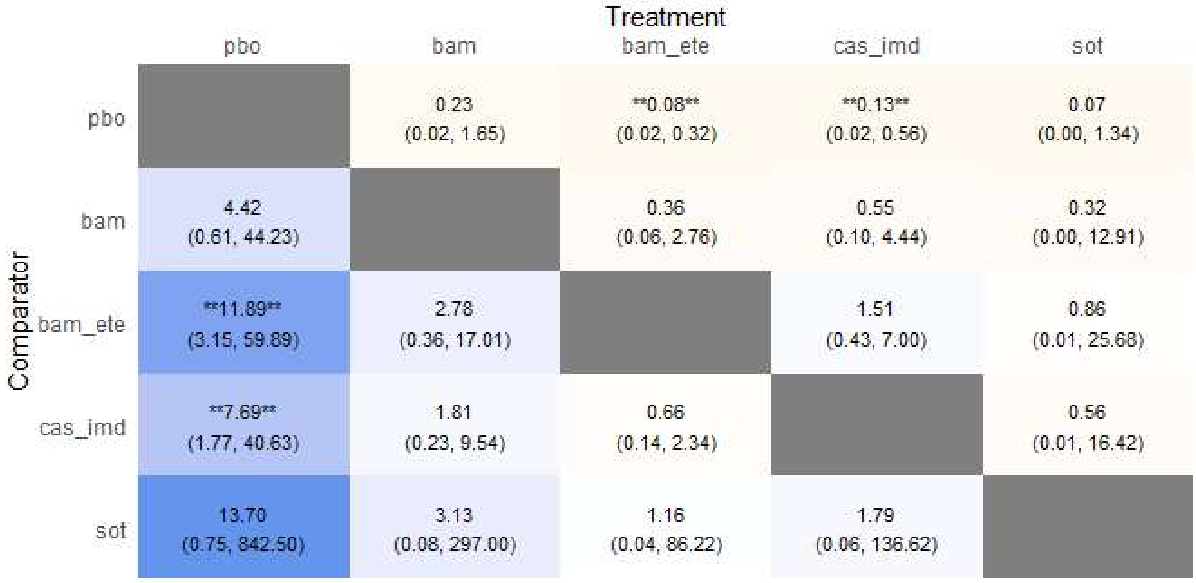

Figure 4.2: Pairwise comparisons: estimates of relative risk (posterior median and 95% CrI) for the outcome of Mortality

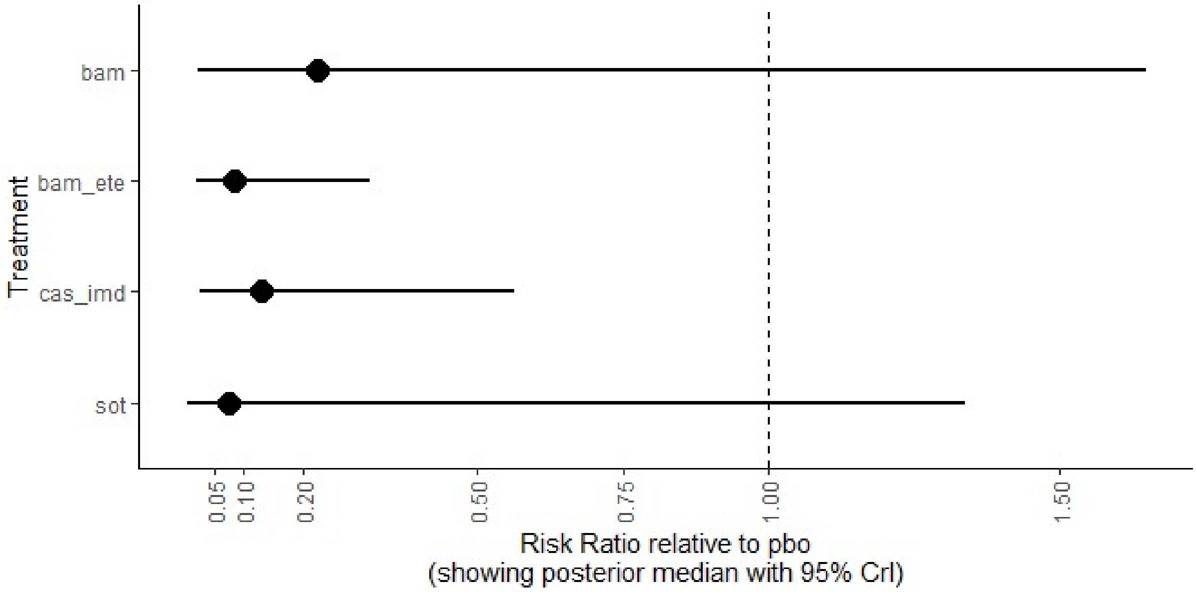

Figure 4.3: Estimes of relative risk for each mAB versus placebo (posterior median and 95% CrI) for the outcome of Mortality

4.2 Hospitalisation

Summary of Evidence Network

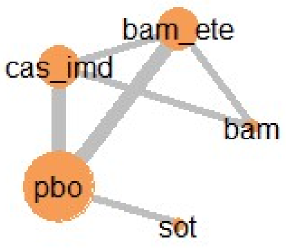

Figure 4.4: Network diagram, Hospitalisation

Table 4.2: Summary of evidence network, Hospitalisation

Characteristic	Value
Number of Interventions	5
Number of Studies	6
Total Number of Patients in Network	8976
Total Possible Pairwise Comparisons	10
Total Number of Pairwise Comparisons With Direct Data	6
Is the network connected?	TRUE
Number of Two-arm Studies	5
Number of Multi-Arms Studies	1
Total Number of Events in Network	494
Number of Studies With No Zero Events	6
Number of Studies With At Least One Zero Event	0
Number of Studies with All Zero Events	0

Table 4.2: Summary of comparison data, Hospitalisation

comparison	n.studies	n.patients	n.outcomes	proportion
bam vs. bam_ete	1	1013	146	0.1441264

comparison	n.studies	n.patients	n.outcomes	proportion
bam vs. cas_imd	1	1050	148	0.1409524
bam_ete vs. cas_imd	1	1807	262	0.1449917
bam_ete vs. pbo	2	1804	62	0.0343681
cas_imd vs. pbo	2	4180	119	0.0284689
pbo vs. sot	1	1057	35	0.0331126

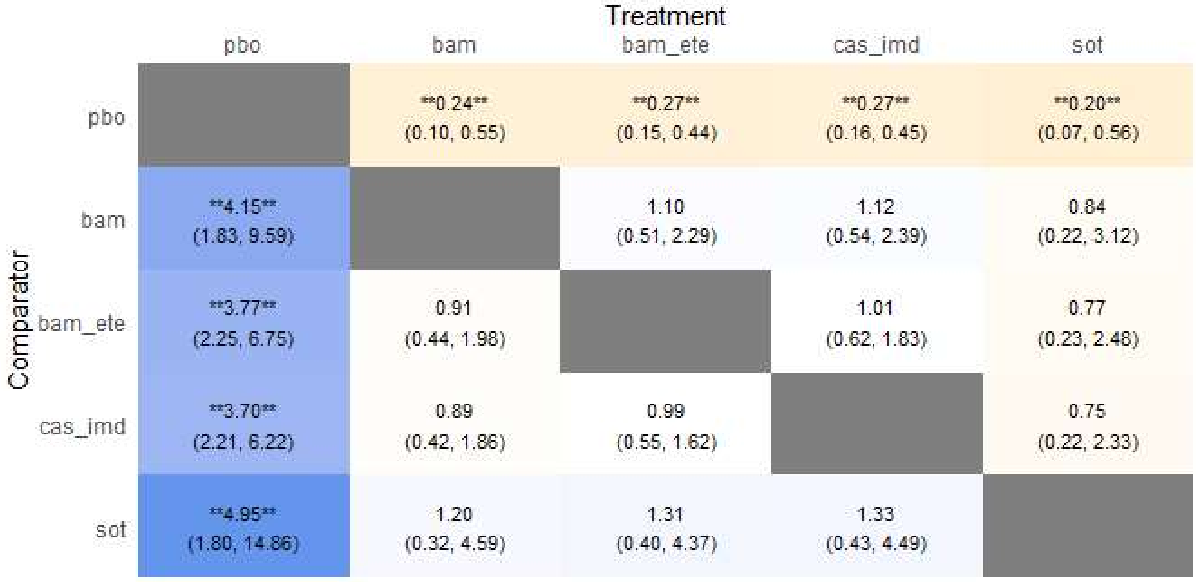

Figure 4.5: Pairwise comparisons: estimates of relative risk (posterior median and 95% CrI) for the outcome of Hospitalisation

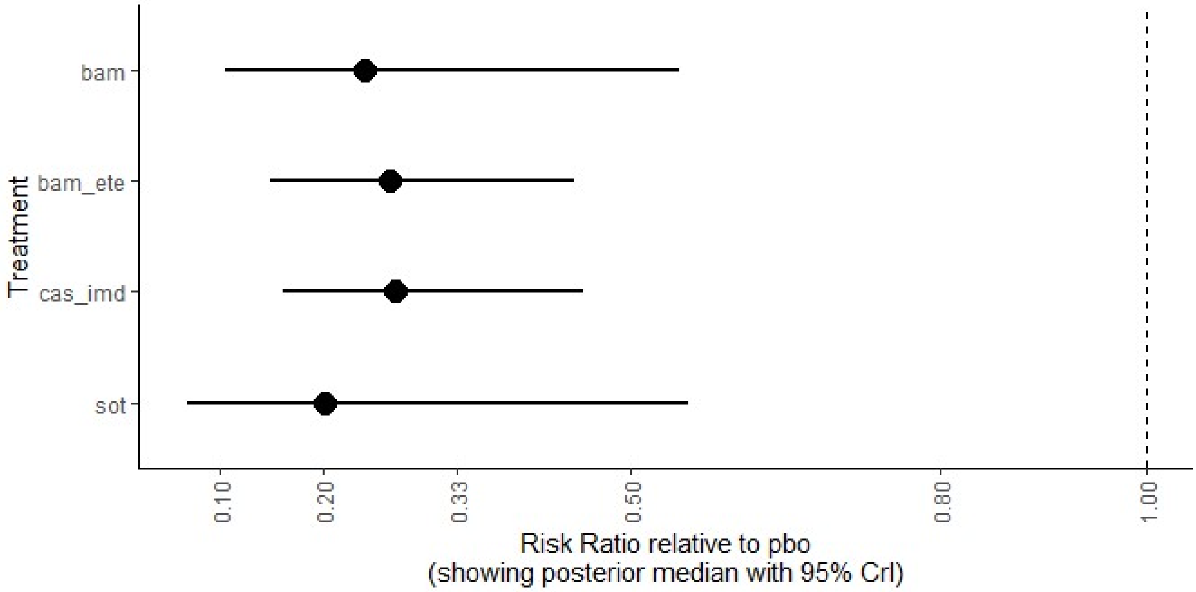

Figure 4.6: Estimes of relative risk for each mAB versus placebo (posterior median and 95% CrI) for the outcome of Hospitalisation

4.3 Invasive Ventilation

Summary of Evidence Network

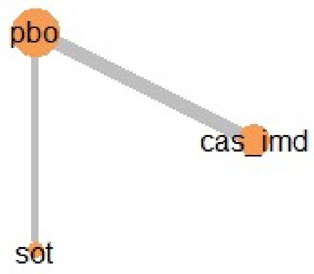

Figure 4.7: Network diagram, Invasive Ventilation

Table 4.3: Summary of evidence network, Invasive Ventilation

Characteristic	Value
Number of Interventions	3
Number of Studies	3
Total Number of Patients in Network	5237
Total Possible Pairwise Comparisons	3
Total Number of Pairwise Comparisons With Direct Data	2
Is the network connected?	TRUE
Number of Two-arm Studies	3
Number of Multi-Arms Studies	0
Total Number of Events in Network	14
Number of Studies With No Zero Events	2
Number of Studies With At Least One Zero Event	1
Number of Studies with All Zero Events	0

Table 4.3: Summary of comparison data, Invasive Ventilation

comparison	n.studies	n.patients	n.outcomes	proportion
cas_imd vs. pbo	2	4180	10	0.0023923
pbo vs. sot	1	1057	4	0.0037843

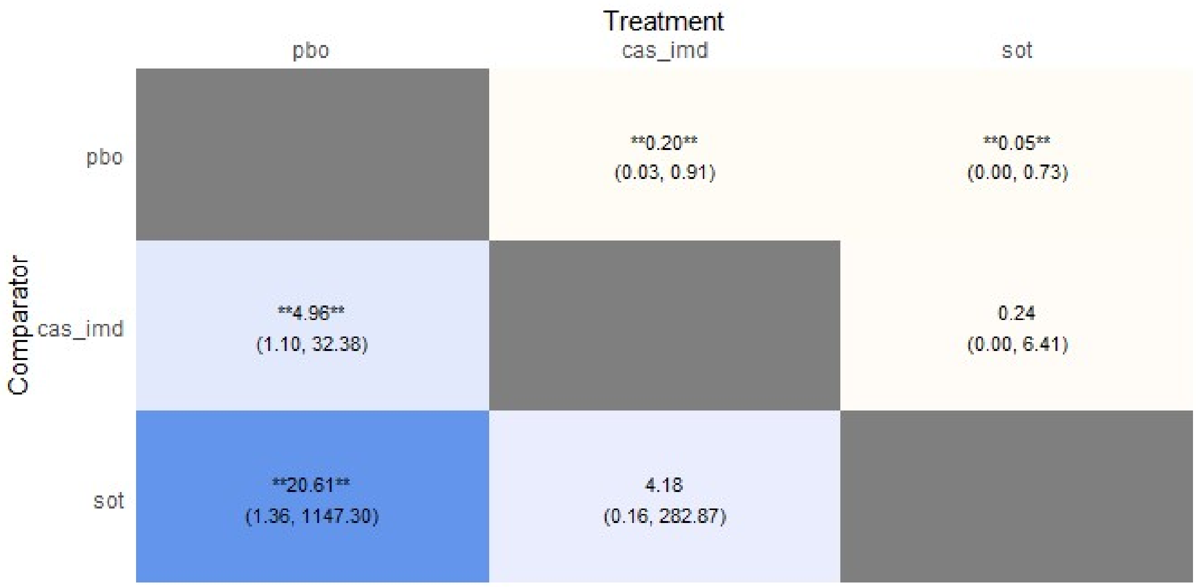

Figure 4.8: Pairwise comparisons: estimates of relative risk (posterior median and 95% CrI) for the outcome of Invasive Ventilation

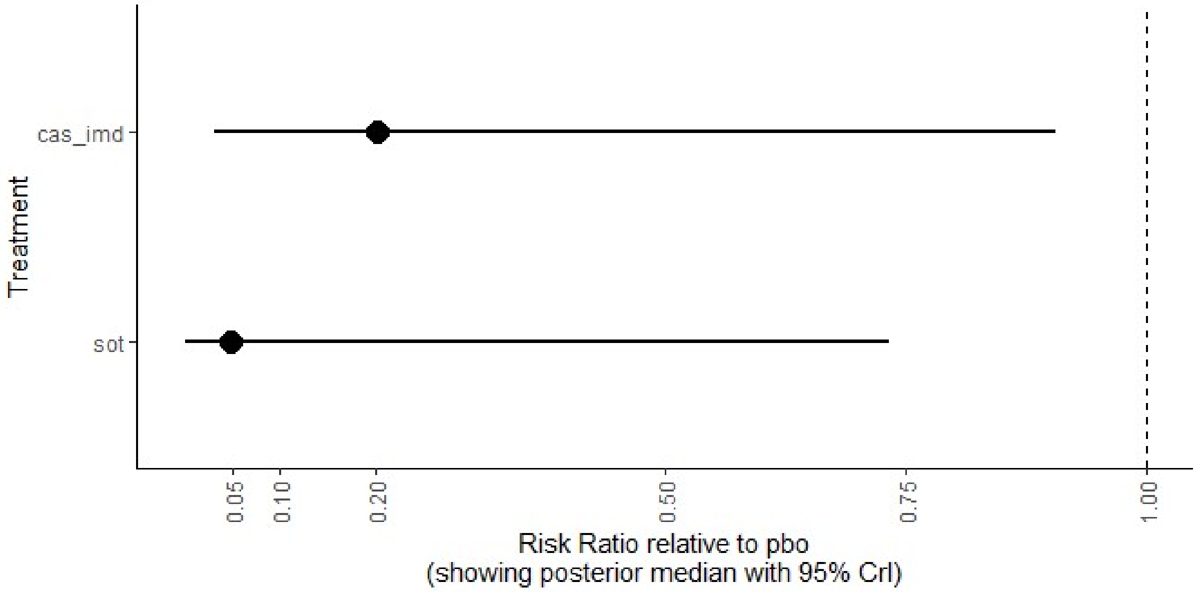

Figure 4.9: Estimes of relative risk for each mAB versus placebo (posterior median and 95% CrI) for the outcome of Invasive Ventilation

4.4 ICU Admission

Summary of Evidence Network

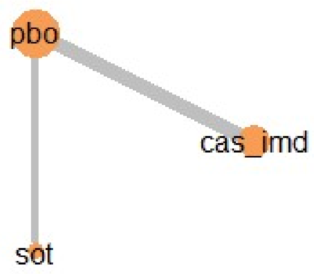

Figure 4.10: Network diagram, ICU Admission

Table 4.4: Summary of evidence network, ICU Admission

Characteristic	Value
Number of Interventions	3
Number of Studies	3
Total Number of Patients in Network	5237
Total Possible Pairwise Comparisons	3
Total Number of Pairwise Comparisons With Direct Data	2
Is the network connected?	TRUE
Number of Two-arm Studies	3
Number of Multi-Arms Studies	0
Total Number of Events in Network	44
Number of Studies With No Zero Events	2
Number of Studies With At Least One Zero Event	1
Number of Studies with All Zero Events	0

Table 4.4: Summary of comparison data, ICU Admission

comparison	n.studies	n.patients	n.outcomes	proportion
cas_imd vs. pbo	2	4180	34	0.0081340

comparison	n.studies	n.patients	n.outcomes	proportion
pbo vs. sot	1	1057	10	0.0094607

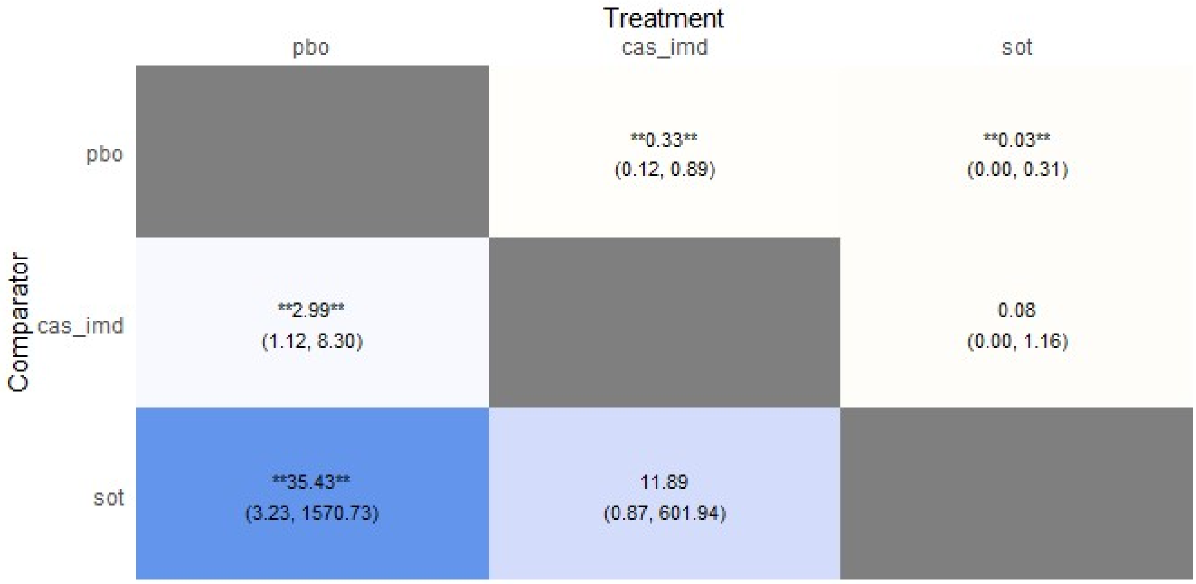

Figure 4.11: Pairwise comparisons: estimates of relative risk (posterior median and 95% CrI) for the outcome of ICU Admission

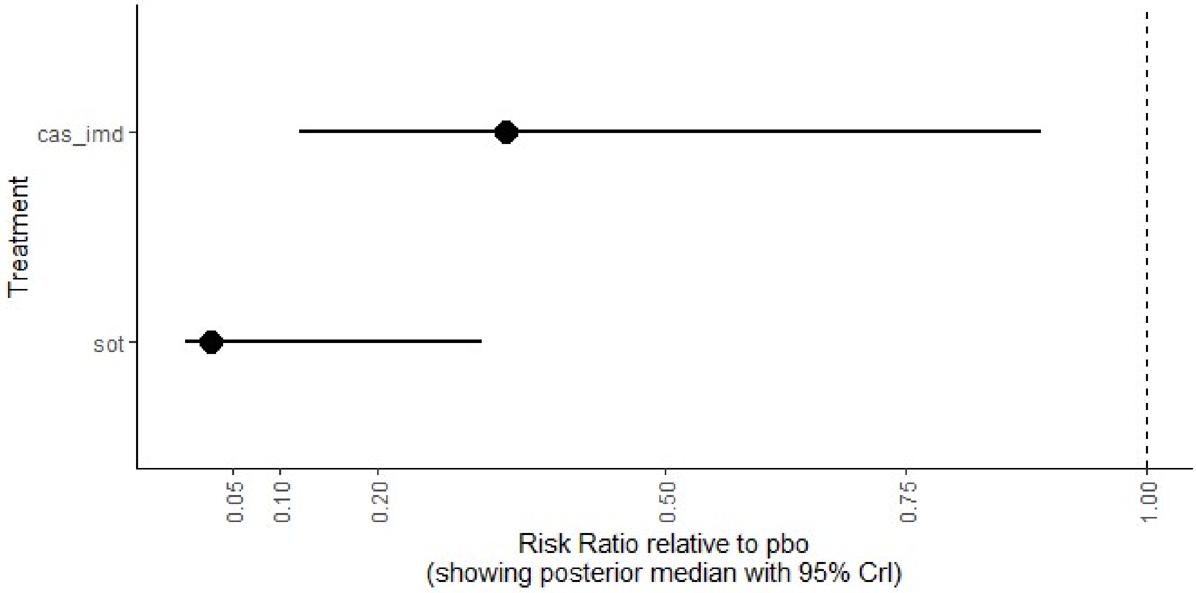

Figure 4.12: Estimes of relative risk for each mAB versus placebo (posterior median and 95% CrI) for the outcome of ICU Admission

4.5 Infusion-related AEs

Summary of Evidence Network

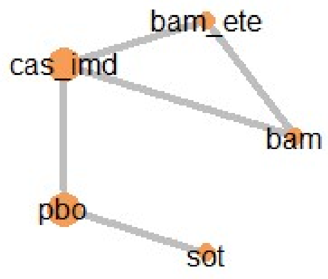

Figure 4.13: Network diagram, Infusion-related AEs

Table 4.5: Summary of evidence network, Infusion-related AEs

Characteristic	Value
Number of Interventions	5
Number of Studies	3
Total Number of Patients in Network	7503
Total Possible Pairwise Comparisons	10
Total Number of Pairwise Comparisons With Direct Data	5
Is the network connected?	TRUE
Number of Two-arm Studies	2
Number of Multi-Arms Studies	1
Total Number of Events in Network	36
Number of Studies With No Zero Events	1
Number of Studies With At Least One Zero Event	2
Number of Studies with All Zero Events	0

Table 4.5: Summary of comparison data, Infusion-related AEs

comparison	n.studies	n.patients	n.outcomes	proportion
bam vs. bam_ete	1	1013	12	0.0118460

comparison	n.studies	n.patients	n.outcomes	proportion
bam vs. cas_imd	1	1050	9	0.0085714
bam_ete vs. cas_imd	1	1807	21	0.0116215
cas_imd vs. pbo	1	4519	3	0.0006639
pbo vs. sot	1	1049	12	0.0114395

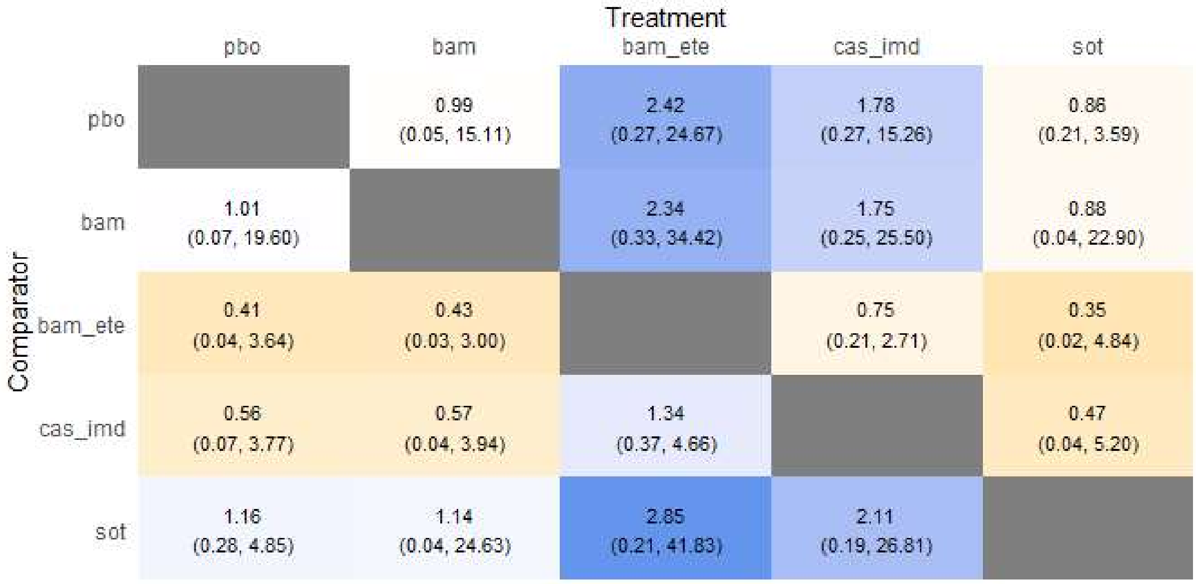

Figure 4.14: Pairwise comparisons: estimates of relative risk (posterior median and 95% CrI) for the outcome of Infusion-related AEs

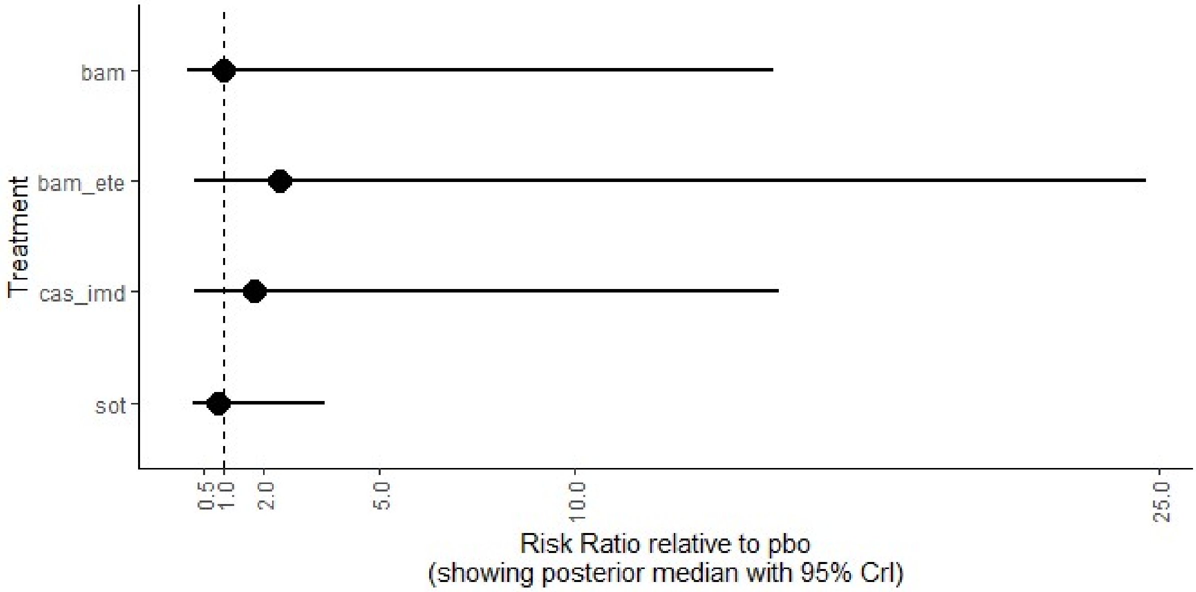

Figure 4.15: Estimes of relative risk for each mAB versus placebo (posterior median and 95% CrI) for the outcome of Infusion-related AEs

4.6 Serious AEs

Summary of Evidence Network

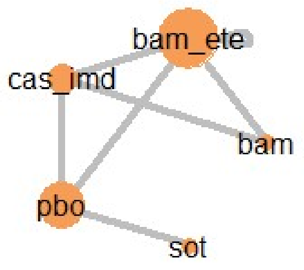

Figure 4.16: Network diagram, Serious AEs

Table 4.6: Summary of evidence network, Serious AEs

Characteristic	Value
Number of Interventions	5
Number of Studies	5
Total Number of Patients in Network	9307
Total Possible Pairwise Comparisons	10
Total Number of Pairwise Comparisons With Direct Data	7
Is the network connected?	TRUE
Number of Two-arm Studies	4
Number of Multi-Arms Studies	1
Total Number of Events in Network	175
Number of Studies With No Zero Events	4
Number of Studies With At Least One Zero Event	1
Number of Studies with All Zero Events	0

Table 4.6: Summary of comparison data, Serious AEs

comparison	n.studies	n.patients	n.outcomes	proportion
bam vs. bam_ete	1	1013	1	0.0009872

comparison	n.studies	n.patients	n.outcomes	proportion
bam vs. cas_imd	1	1050	4	0.0038095
bam_ete vs. bam_ete	4	3076	32	0.0104031
bam_ete vs. cas_imd	1	1807	5	0.0027670
bam_ete vs. pbo	1	1035	12	0.0115942
cas_imd vs. pbo	1	4519	107	0.0236778
pbo vs. sot	1	1049	43	0.0409914

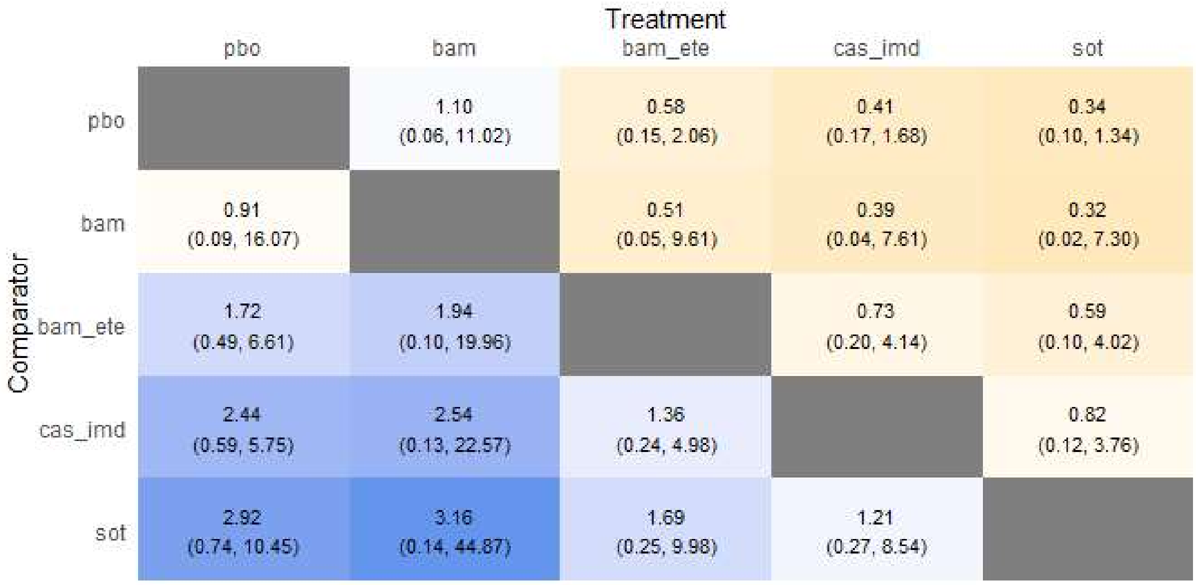

Figure 4.17: Pairwise comparisons: estimates of relative risk (posterior median and 95% CrI) for the outcome of Serious AEs

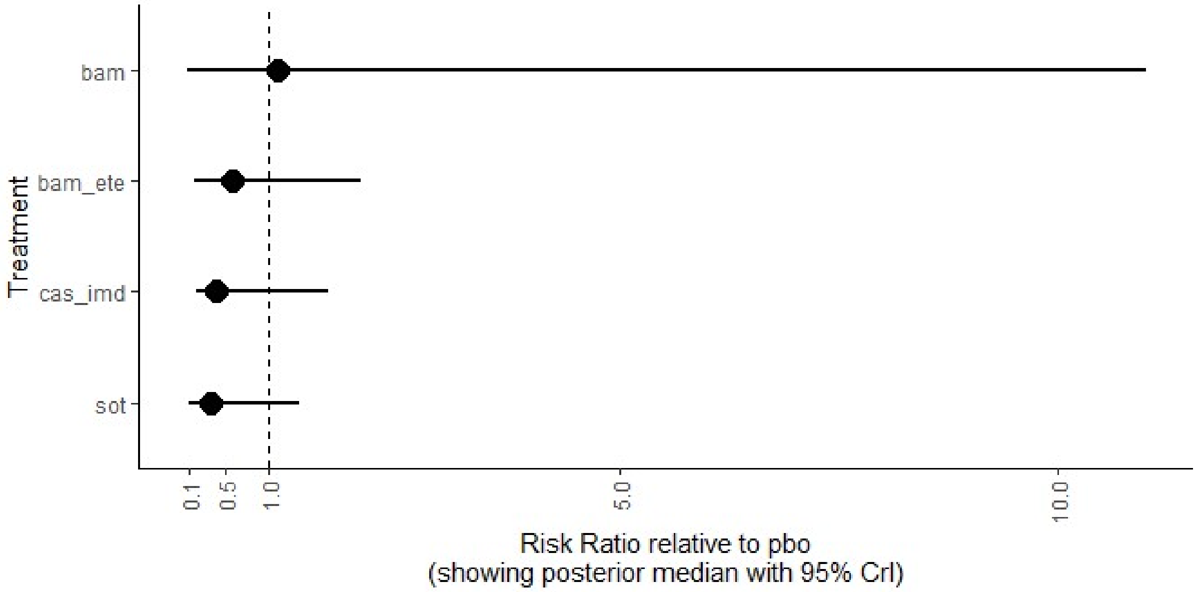

Figure 4.18: Estimes of relative risk for each mAB versus placebo (posterior median and 95% CrI) for the outcome of Serious AEs

4.7 Pooled mAB effects

Table 4.7: Estimated pooled effects for mABs versus placebo, all outcomes

Outcome	RR..mABs.vs.Placebo
Mortality	0.07 (0.01 to 0.25)
Hospitalisation	0.26 (0.18 to 0.36)
Invasive Ventilation	0.13 (0.02 to 0.51)
ICU Admission	0.22 (0.08 to 0.54)
Infusion-related AEs	1.08 (0.36 to 3.51)
Serious AEs	0.39 (0.22 to 0.87)

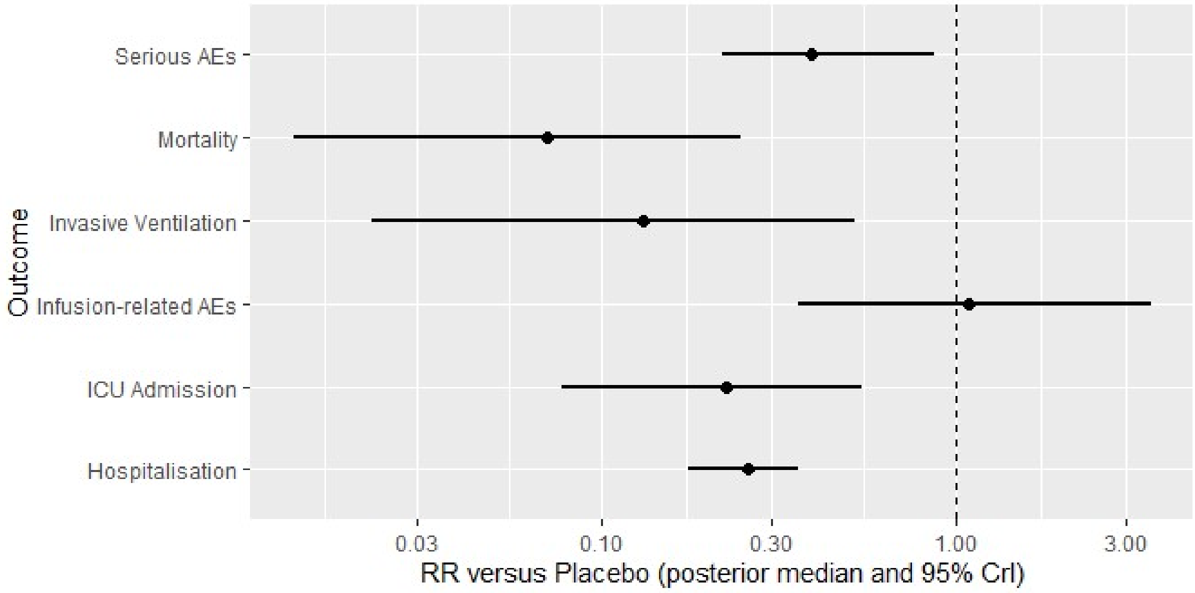

Figure 4.19: Estimated pooled effects for mABs versus placebo, all outcomes

4.8 Inclusion of regdanvimab as intervention

In this section we explore the effect of including results from (Eom et al., 2021), which compared regdanvimab and placebo in a mixed-risk population, in the NMA. This study was excluded during the rapid review as results were not reported separately for the high-risk subpopulation. It has been included as a scenario analysis as it has been licensed in the EU for the population of interest to this review (adults with COVID-19 who are at increased risk of progressing to severe COVID-19). The study contained three arms: regdanvimab 40mg/kg, regdanvimab 80mg/kg, and placebo. For consistency with other comparisons in the network, outcomes for regdanvimab were pooled across 40mg/kg and 80mg/kg arms.

All relevant outcomes were considered for inclusion in this scenario analysis:

- Mortality: excluded as no events in either arm
- Hospitalisation: included
- Invasive ventilation: excluded as no events observed in placebo arm (1 event in treatment arm), therefore it was not possible to estimate a relative-risk for regdanvimab versus placebo for this outcome. While an RR for placebo versus regdanvimab could in theory be estimated this would be highly uncertain due to there being only a sinlge event in the regdanvimab arm.
- ICU admission: excluded as no events in either arm
- Infusion-related AEs: included
- Serious AEs: excluded as no events in either arm

4.8.1 Hospitalisation

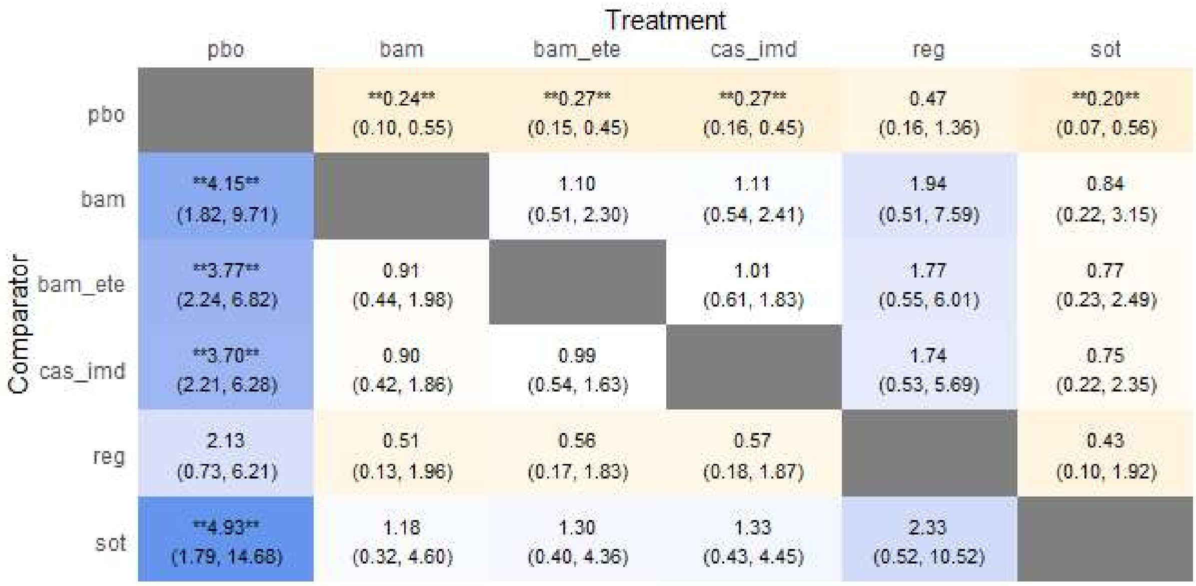

Comparator \ Treatment	pbo	bam	bam_ete	cas_imd	reg	sot
pbo		**0.24** (0.10, 0.55)	**0.27** (0.15, 0.45)	**0.27** (0.16, 0.45)	0.47 (0.16, 1.36)	**0.20** (0.07, 0.56)
bam	**4.15** (1.82, 9.71)		1.10 (0.51, 2.30)	1.11 (0.54, 2.41)	1.94 (0.51, 7.59)	0.84 (0.22, 3.15)
bam_ete	**3.77** (2.24, 6.82)	0.91 (0.44, 1.98)		1.01 (0.61, 1.83)	1.77 (0.55, 6.01)	0.77 (0.23, 2.49)
cas_imd	**3.70** (2.21, 6.28)	0.90 (0.42, 1.86)	0.99 (0.54, 1.63)		1.74 (0.53, 5.69)	0.75 (0.22, 2.35)
reg	2.13 (0.73, 6.21)	0.51 (0.13, 1.96)	0.56 (0.17, 1.83)	0.57 (0.18, 1.87)		0.43 (0.10, 1.92)
sot	**4.93** (1.79, 14.68)	1.18 (0.32, 4.60)	1.30 (0.40, 4.36)	1.33 (0.43, 4.45)	2.33 (0.52, 10.52)	

Figure 4.20: Pairwise comparisons: estimates of relative risk (posterior median and 95% CrI) for the outcome of Hospitalisation, scenario analysis including reganvimab data from (Eom et al., 2021)

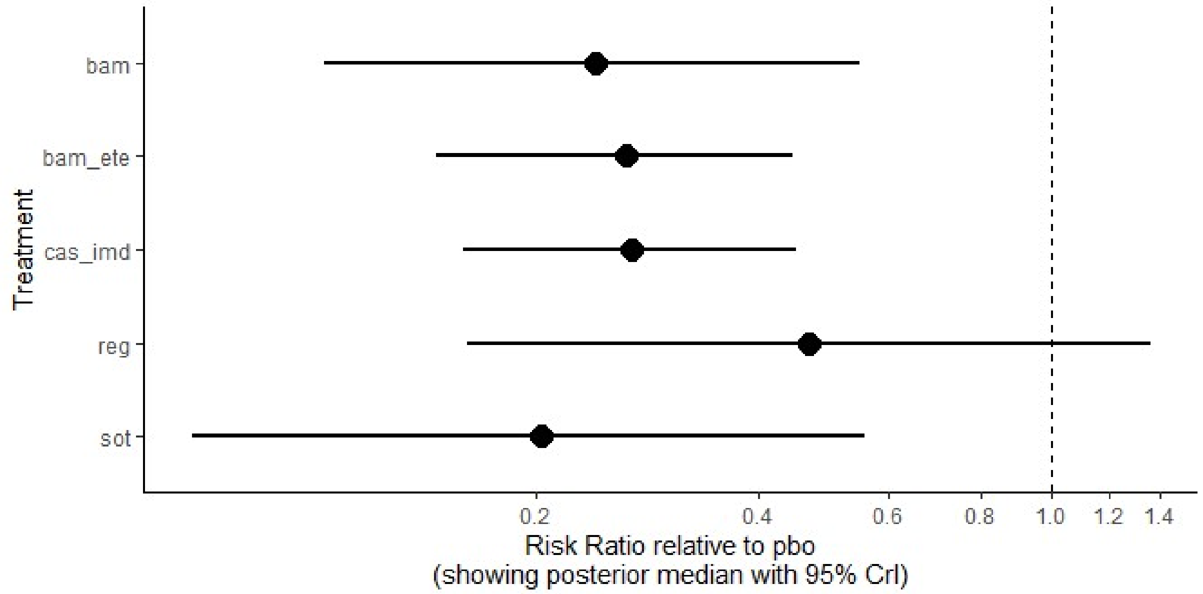

Figure 4.21: Relative risk estimates versus placebo (posterior median and 95% CrI) for the outcome of Hospitalisation, scenario analysis including reganvimab data from (Eom et al., 2021)

4.8.2 Infusion-related AEs

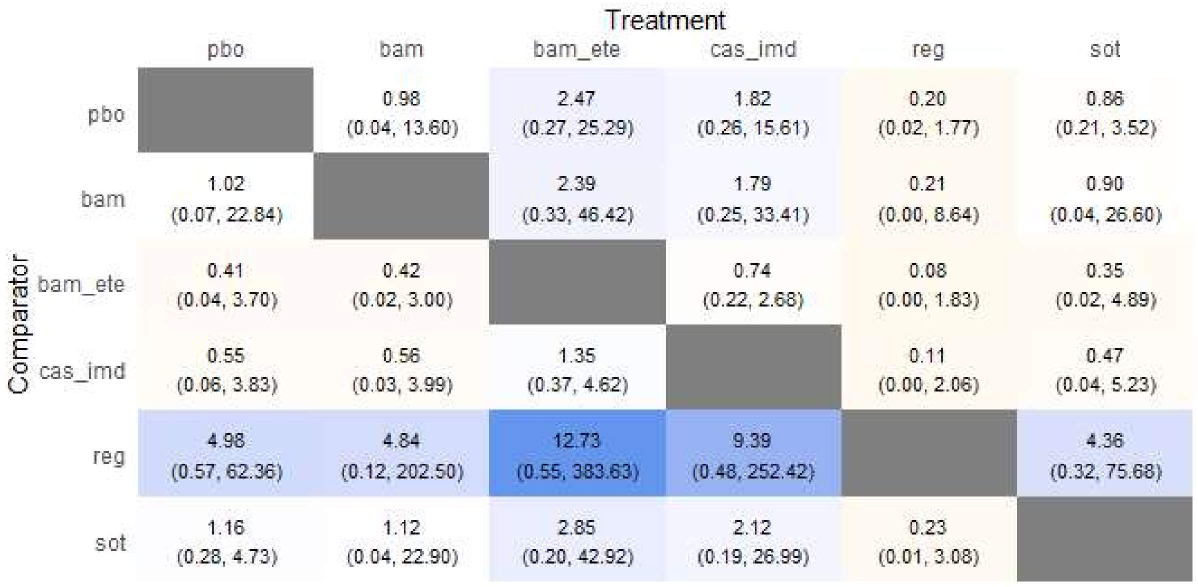

Figure 4.22: Pairwise comparisons: estimates of relative risk (posterior median and 95% CrI) for the outcome of Infusion-related AEs, scenario analysis including reganvimab data from (Eom et al., 2021)

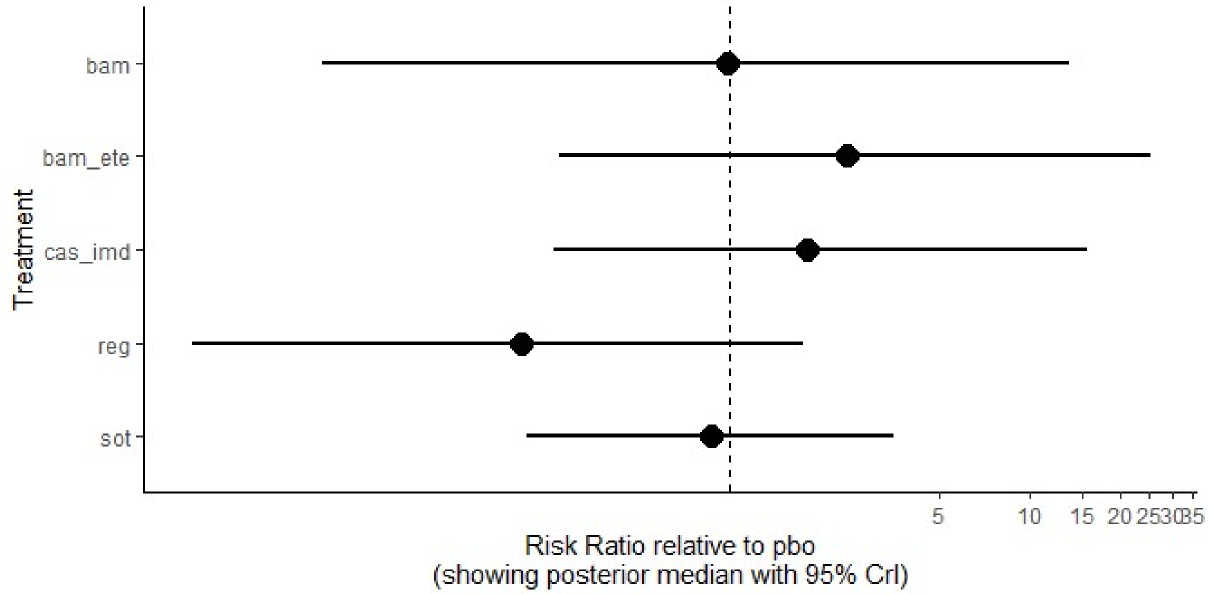

Figure 4.23: Relative risk estimates versus placebo (posterior median and 95% CrI) for the outcome of Infusion-related AEs, scenario analysis including reganvimab data from (Eom et al., 2021)

4.9 Absolute Risk Reductions

For all dichotomous outcomes, baseline event rates under SoC have been estimated by pooling the rates from the SoC arms across trials using a Bayesian random-effects model, analogous to the model used for relative treatment effects but with a random intercept term only (see NICE TSD 5 (Dias et al., 2013b)). In all cases, event rates from (Eom et al., 2021) have been excluded from this calculation as this study included non-high-risk patients, who are likely to exhibit lower baseline event rates for most outcomes compared with the target population.

Absolute risks and risk differences for regdanvimab have therefore been estimated by applying the relative effects for regdanvimab versus placebo, to baseline event rates estimated from the remaining studies. This approaches assumes that the relative effect of regdanvimab versus placebo will generalise to the higher-risk populations of the other studies.

4.9.1 Mortality

Table 4.8: Absolute risks and risk difference per 1000 (posterior median and 95% CrI) for the outcome of Mortality

Treatment	Absolute.risks.per.1.000	Comparison	Risk.difference.per.1.000
pbo	5.57 (0.76, 31.99)	pbo_vs_pbo	0.00 (0.00, 0.00)
bam	1.19 (0.06, 16.86)	bam_vs_pbo	-3.66 (-23.21, 3.35)
bam_ete	0.45 (0.04, 4.24)	bam_ete_vs_pbo	-4.98 (-28.57, -0.66)
cas_imd	0.70 (0.06, 7.19)	cas_imd_vs_pbo	-4.59 (-27.30, -0.52)
sot	0.39 (0.00, 12.94)	sot_vs_pbo	-4.44 (-27.12, 1.85)

Treatment	Absolute.risks.per.1.000	Comparison	Risk.difference.per.1.000
pooled_mABs	0.37 (0.03, 3.26)	pooled_mABs_vs_pbo	-5.09 (-29.77, -0.68)

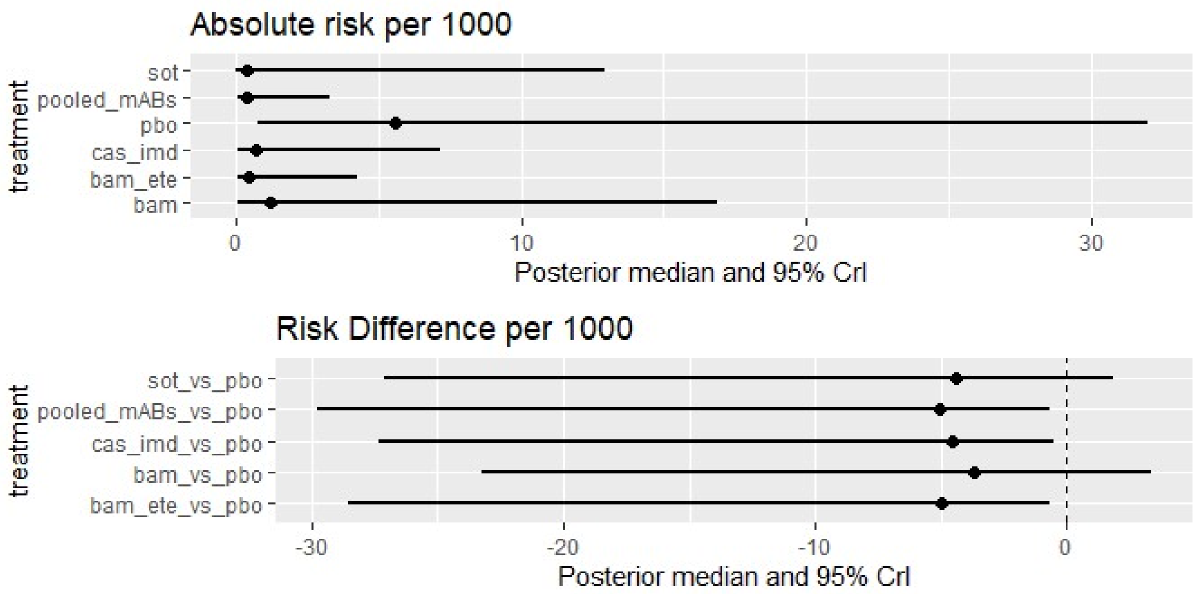

Figure 4.24: Absolute risks per 1000 for each treatment (top) and risk difference per 1000 patients for each treatment versus placebo (bottom) for the outcome of Mortality

4.9.2 Hospitalisation

Table 4.8: Absolute risks and risk difference per 1000 (posterior median and 95% CrI) for the outcome of Hospitalisation

Treatment	Absolute.risks.per.1.000	Comparison	Risk.difference.per.1.000
pbo	49.51 (26.79, 89.50)	pbo_vs_pbo	0.00 (0.00, 0.00)
bam	11.94 (4.37, 31.83)	bam_vs_pbo	-36.87 (-67.96, -16.06)
bam_ete	13.18 (5.65, 28.29)	bam_ete_vs_pbo	-36.17 (-66.43, -17.95)
cas_imd	13.39 (6.11, 28.84)	cas_imd_vs_pbo	-35.77 (-65.10, -18.09)
sot	10.02 (2.98, 31.50)	sot_vs_pbo	-38.65 (-70.99, -16.54)
pooled_mABs	12.59 (6.36, 24.56)	pooled_mABs_vs_pbo	-36.71 (-66.55, -19.63)
*reg	23.27 (6.89, 75.05)	*reg_vs_pbo	-25.43 (-54.63, 17.01)

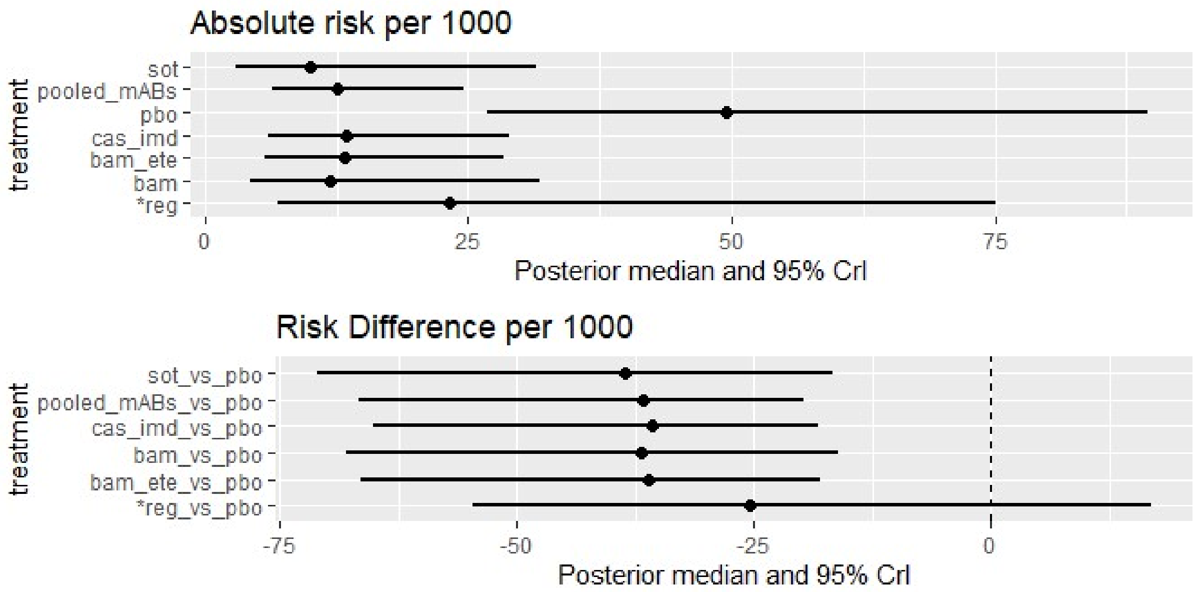

Figure 4.25: Absolute risks per 1000 for each treatment (top) and risk difference per 1000 patients for each treatment versus placebo (bottom) for the outcome of Hospitalisation

4.9.3 Invasive Ventilation

Table 4.8: Absolute risks and risk difference per 1000 (posterior median and 95% CrI) for the outcome of Invasive Ventilation

Treatment	Absolute.risks.per.1.000	Comparison	Risk.difference.per.1.000
pbo	4.37 (1.30, 13.92)	pbo_vs_pbo	0.00 (0.00, 0.00)
cas_imd	0.86 (0.10, 5.85)	cas_imd_vs_pbo	-3.26 (-10.65, -0.17)
sot	0.21 (0.00, 3.93)	sot_vs_pbo	-3.89 (-12.59, -0.60)
pooled_mABs	0.55 (0.07, 3.40)	pooled_mABs_vs_pbo	-3.64 (-11.72, -0.92)

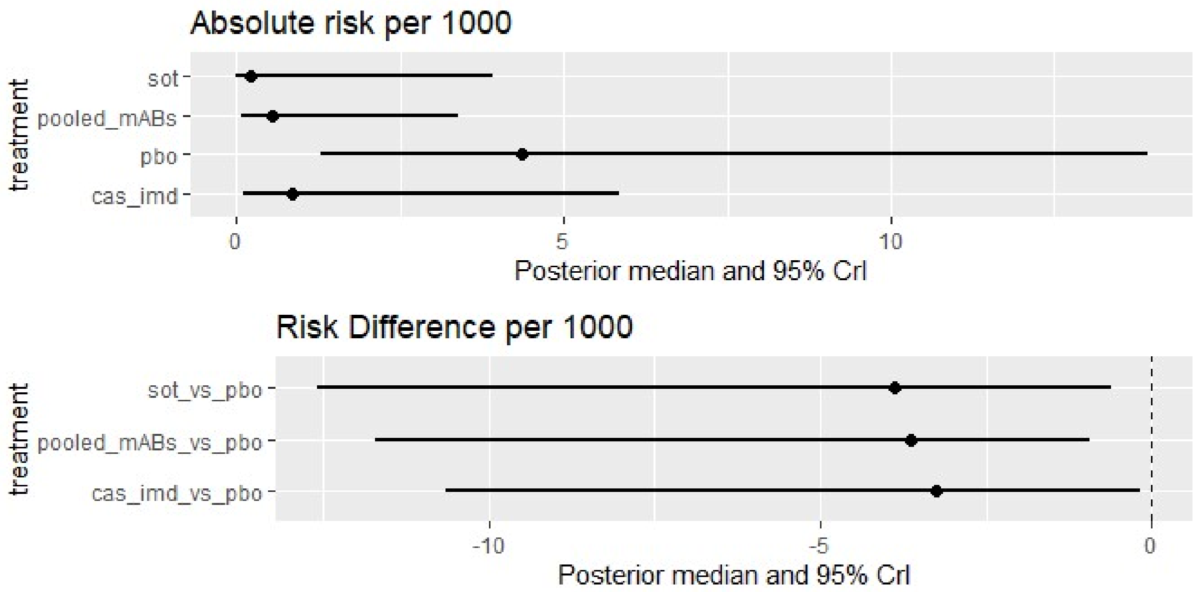

Figure 4.26: Absolute risks per 1000 for each treatment (top) and risk difference per 1000 patients for each treatment versus placebo (bottom) for the outcome of Invasive Ventilation

4.9.4 ICU Admission

Table 4.8: Absolute risks and risk difference per 1000 (posterior median and 95% CrI) for the outcome of ICU Admission

Treatment	Absolute.risks.per.1.000	Comparison	Risk.difference.per.1.000
pbo	13.17 (5.06, 32.52)	pbo_vs_pbo	0.00 (0.00, 0.00)
cas_imd	4.39 (1.09, 16.13)	cas_imd_vs_pbo	-8.37 (-22.31, -0.87)
sot	0.36 (0.01, 4.79)	sot_vs_pbo	-12.43 (-30.90, -4.28)
pooled_mABs	2.91 (0.75, 10.25)	pooled_mABs_vs_pbo	-9.93 (-25.44, -3.24)

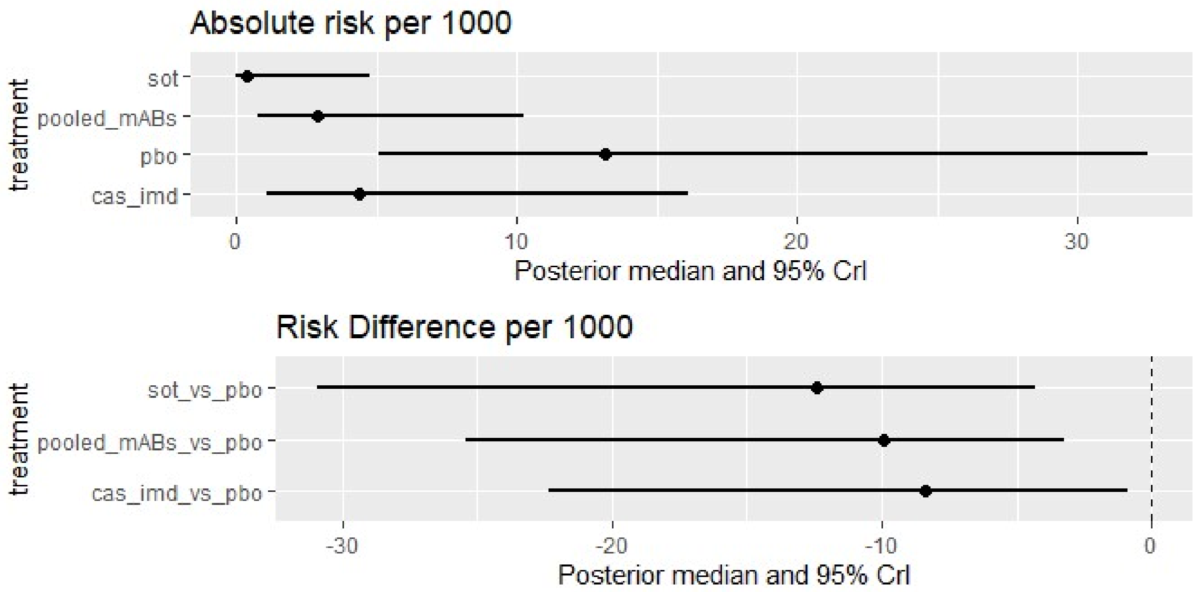

Figure 4.27: Absolute risks per 1000 for each treatment (top) and risk difference per 1000 patients for each treatment versus placebo (bottom) for the outcome of ICU Admission

4.9.5 Infusion-related AEs

Table 4.8: Absolute risks and risk difference per 1000 (posterior median and 95% CrI) for the outcome of Infusion-related AEs

Treatment	Absolute.risks.per.1.000	Comparison	Risk.difference.per.1.000
pbo	2.30 (0.13, 27.78)	pbo_vs_pbo	0.00 (0.00, 0.00)
bam	2.22 (0.04, 84.88)	bam_vs_pbo	-0.01 (-10.51, 67.84)
bam_ete	5.57 (0.16, 157.93)	bam_ete_vs_pbo	2.61 (-3.75, 134.49)
cas_imd	4.12 (0.12, 99.29)	cas_imd_vs_pbo	1.31 (-5.02, 83.63)
sot	1.95 (0.09, 33.88)	sot_vs_pbo	-0.15 (-7.96, 13.83)
pooled_mABs	2.43 (0.12, 37.50)	pooled_mABs_vs_pbo	0.08 (-5.06, 16.26)
*reg	0.45 (0.01, 12.23)	*reg_vs_pbo	-1.44 (-20.93, 2.06)

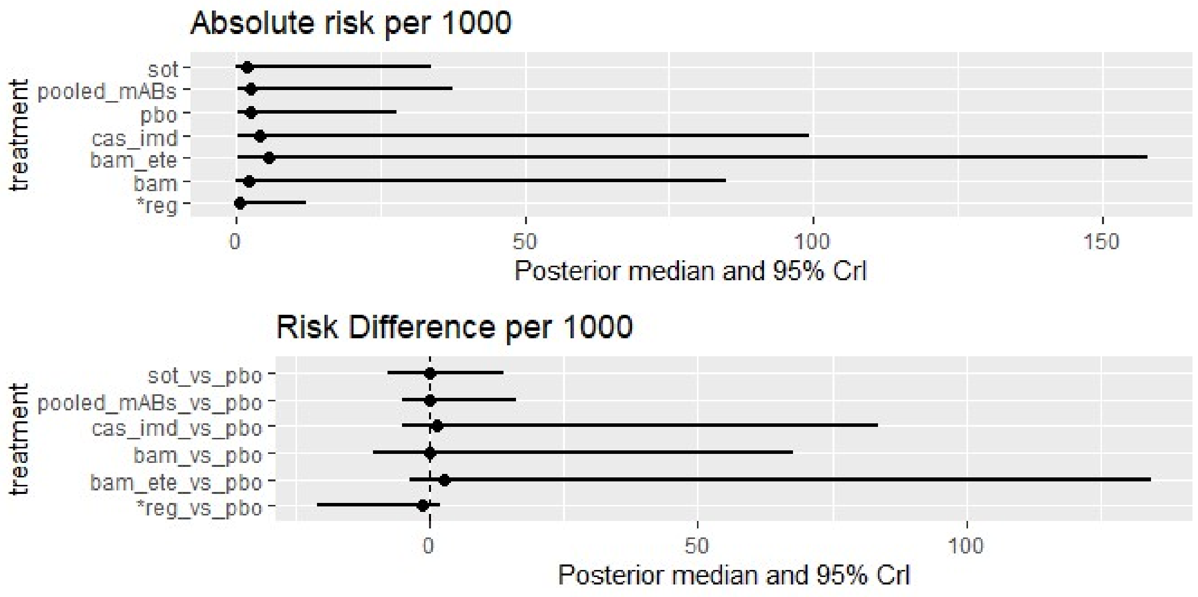

Figure 4.28: Absolute risks per 1000 for each treatment (top) and risk difference per 1000 patients for each treatment versus placebo (bottom) for the outcome of Infusion-related AEs

4.9.6 Serious AEs

Table 4.8: Absolute risks and risk difference per 1000 (posterior median and 95% CrI) for the outcome of Serious AEs

Treatment	Absolute.risks.per.1.000	Comparison	Risk.difference.per.1.000
pbo	31.56 (4.74, 155.22)	pbo_vs_pbo	0.00 (0.00, 0.00)
bam	33.57 (1.19, 550.43)	bam_vs_pbo	2.35 (-66.81, 461.39)
bam_ete	17.87 (2.00, 133.37)	bam_ete_vs_pbo	-10.33 (-77.12, 44.18)
cas_imd	13.45 (1.68, 108.51)	cas_imd_vs_pbo	-15.40 (-87.92, 26.03)
sot	10.73 (1.15, 85.30)	sot_vs_pbo	-18.21 (-100.65, 8.31)
pooled_mABs	12.31 (1.71, 70.08)	pooled_mABs_vs_pbo	-18.07 (-93.31, -1.47)

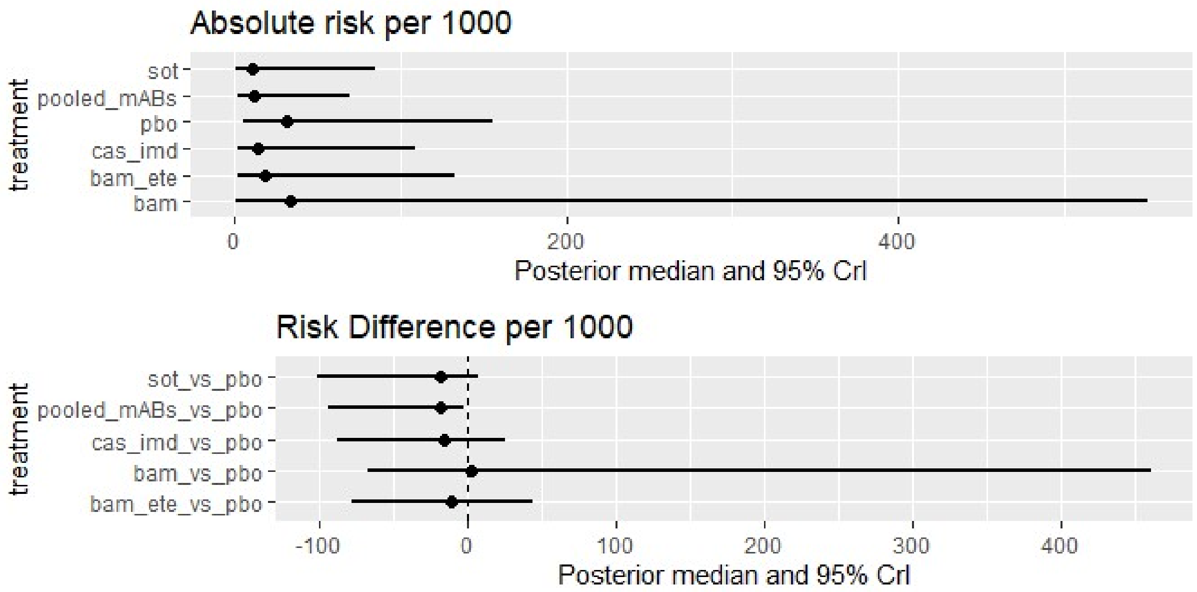

Figure 4.29: Absolute risks per 1000 for each treatment (top) and risk difference per 1000 patients for each treatment versus placebo (bottom) for the outcome of Serious AEs

5 Sensitivity Analysis

5.1 Treatment effects prior

For all outcomes we investigate the following alternative priors for the treatment effect parameters:

1. More informative prior: N(0,1.175^2). This distribution gives a 95% prior probability that no relative risk between any pair of treatments in the network exceeds 10 (note that 0.175=log(10)/1.96).

2. Less informative prior: N(0,4.700^2). This distribution gives a 95% prior probability that no relative risk between any pair of treatments in the network exceeds 10,000 (note that 4.700=log(10000)/1.96).

5.1.1 Mortality

Table 4.9: NMA sensitivity analysis results for the outcome of Mortality, using a prior distribution of N(0,1.2^2) for the treatment effect parameters.

	pbo	bam	bam_ete	cas_imd	sot
pbo	pbo	0.59 (0.13 to 2.71)	0.19 (0.06 to 0.55)	0.29 (0.09 to 0.99)	0.36 (0.05 to 2.14)
bam	1.68 (0.37 to 7.85)	bam	0.31 (0.07 to 1.54)	0.48 (0.11 to 2.49)	0.60 (0.05 to 6.20)

	pbo	bam	bam_ete	cas_imd	sot
bam_ete	5.39 (1.81 to 16.42)	3.22 (0.65 to 15.22)	bam_ete	1.53 (0.47 to 5.88)	1.91 (0.22 to 15.60)
cas_imd	3.49 (1.01 to 10.91)	2.09 (0.40 to 9.25)	0.65 (0.17 to 2.13)	cas_imd	1.23 (0.13 to 10.08)
sot	2.81 (0.47 to 18.61)	1.68 (0.16 to 18.80)	0.52 (0.06 to 4.53)	0.82 (0.10 to 7.58)	sot

Table 4.10: NMA sensitivity analysis results for the outcome of Mortality, using a prior distribution of N(0,2.8^2) for the treatment effect parameters.

	pbo	bam	bam_ete	cas_imd	sot
pbo	pbo	0.22 (0.02 to 1.59)	0.08 (0.02 to 0.31)	0.13 (0.03 to 0.55)	0.07 (0.00 to 1.29)
bam	4.46 (0.63 to 40.91)	bam	0.36 (0.06 to 2.64)	0.55 (0.11 to 4.18)	0.32 (0.00 to 12.18)
bam_ete	11.98 (3.23 to 60.09)	2.75 (0.38 to 16.83)	bam_ete	1.51 (0.43 to 6.84)	0.87 (0.01 to 23.62)
cas_imd	7.81 (1.81 to 39.17)	1.81 (0.24 to 9.49)	0.66 (0.15 to 2.35)	cas_imd	0.56 (0.01 to 14.90)
sot	13.96 (0.78 to 971.39)	3.15 (0.08 to 309.82)	1.15 (0.04 to 100.99)	1.80 (0.07 to 156.69)	sot

Table 4.11: NMA sensitivity analysis results for the outcome of Mortality, using a prior distribution of N(0,4.7^2) for the treatment effect parameters.

	pbo	bam	bam_ete	cas_imd	sot
pbo	pbo	0.16 (0.01 to 1.29)	0.06 (0.01 to 0.26)	0.10 (0.01 to 0.46)	0.02 (0.00 to 0.91)
bam	6.36 (0.77 to 83.55)	bam	0.39 (0.06 to 3.20)	0.59 (0.11 to 5.37)	0.11 (0.00 to 14.15)
bam_ete	15.63 (3.79 to 113.33)	2.59 (0.31 to 16.46)	bam_ete	1.51 (0.42 to 7.56)	0.27 (0.00 to 25.44)
cas_imd	10.21 (2.16 to 70.54)	1.68 (0.19 to 9.36)	0.66 (0.13 to 2.41)	cas_imd	0.17 (0.00 to 15.90)
sot	56.80 (1.09 to 73837.61)	9.33 (0.07 to 14657.26)	3.69 (0.04 to 5378.52)	5.72 (0.06 to 8514.55)	sot

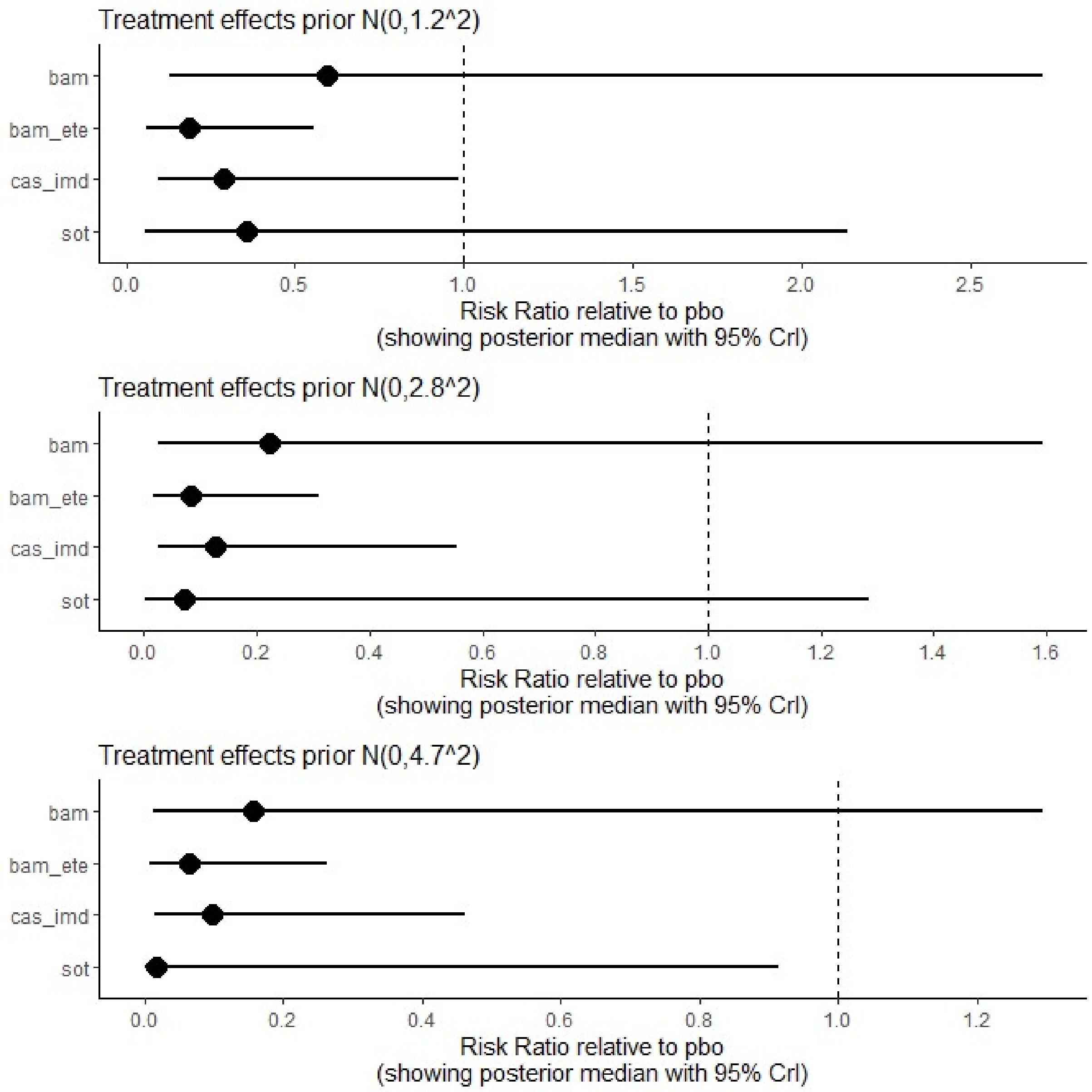

Figure 4.30: Sensitivity analysis using alternative prior distributions for the treatment effect parameter (log relative risk), for the outcome of Mortality. Top: Efficacy prior N(0,1.2^2) (more informative), middle: Efficacy prior N(0,2.8^2) (base case), bottom: Efficacy prior N(0,4.7^2) (less informative)

5.1.2 Hospitalisation

Table 4.12: NMA sensitivity analysis results for the outcome of Hospitalisation, using a prior distribution of N(0,1.2^2) for the treatment effect parameters.

	pbo	bam	bam_ete	cas_imd	sot
pbo	pbo	0.29 (0.14 to 0.73)	0.30 (0.18 to 0.52)	0.30 (0.19 to 0.54)	0.25 (0.10 to 0.68)

	pbo	bam	bam_ete	cas_imd	sot
bam	3.48 (1.38 to 7.10)	bam	1.03 (0.45 to 2.02)	1.04 (0.47 to 2.07)	0.85 (0.25 to 2.79)
bam_ete	3.37 (1.91 to 5.64)	0.97 (0.50 to 2.24)	bam_ete	1.00 (0.60 to 1.82)	0.83 (0.29 to 2.54)
cas_imd	3.34 (1.86 to 5.32)	0.96 (0.48 to 2.12)	1.00 (0.55 to 1.66)	cas_imd	0.82 (0.28 to 2.40)
sot	4.03 (1.48 to 10.39)	1.17 (0.36 to 4.08)	1.20 (0.39 to 3.49)	1.22 (0.42 to 3.54)	sot

Table 4.13: NMA sensitivity analysis results for the outcome of Hospitalisation, using a prior distribution of N(0,2.8^2) for the treatment effect parameters.

	pbo	bam	bam_ete	cas_imd	sot
pbo	pbo	0.24 (0.10 to 0.55)	0.27 (0.15 to 0.45)	0.27 (0.16 to 0.45)	0.20 (0.07 to 0.56)
bam	4.12 (1.83 to 9.67)	bam	1.10 (0.51 to 2.30)	1.11 (0.54 to 2.41)	0.83 (0.21 to 3.13)
bam_ete	3.75 (2.24 to 6.73)	0.91 (0.43 to 1.96)	bam_ete	1.01 (0.62 to 1.83)	0.76 (0.23 to 2.46)
cas_imd	3.69 (2.21 to 6.21)	0.90 (0.42 to 1.85)	0.99 (0.55 to 1.62)	cas_imd	0.75 (0.22 to 2.31)
sot	4.94 (1.80 to 15.04)	1.20 (0.32 to 4.74)	1.31 (0.41 to 4.42)	1.34 (0.43 to 4.53)	sot

Table 4.14: NMA sensitivity analysis results for the outcome of Hospitalisation, using a prior distribution of N(0,4.7^2) for the treatment effect parameters.

	pbo	bam	bam_ete	cas_imd	sot
pbo	pbo	0.23 (0.10 to 0.53)	0.26 (0.14 to 0.44)	0.27 (0.15 to 0.45)	0.19 (0.06 to 0.54)
bam	4.26 (1.90 to 10.21)	bam	1.11 (0.51 to 2.34)	1.13 (0.55 to 2.49)	0.82 (0.21 to 3.16)
bam_ete	3.82 (2.29 to 7.09)	0.90 (0.43 to 1.95)	bam_ete	1.01 (0.61 to 1.85)	0.74 (0.22 to 2.47)
cas_imd	3.75 (2.25 to 6.46)	0.88 (0.40 to 1.83)	0.99 (0.54 to 1.63)	cas_imd	0.72 (0.21 to 2.32)
sot	5.17 (1.86 to 15.97)	1.22 (0.32 to 4.81)	1.35 (0.41 to 4.62)	1.38 (0.43 to 4.73)	sot

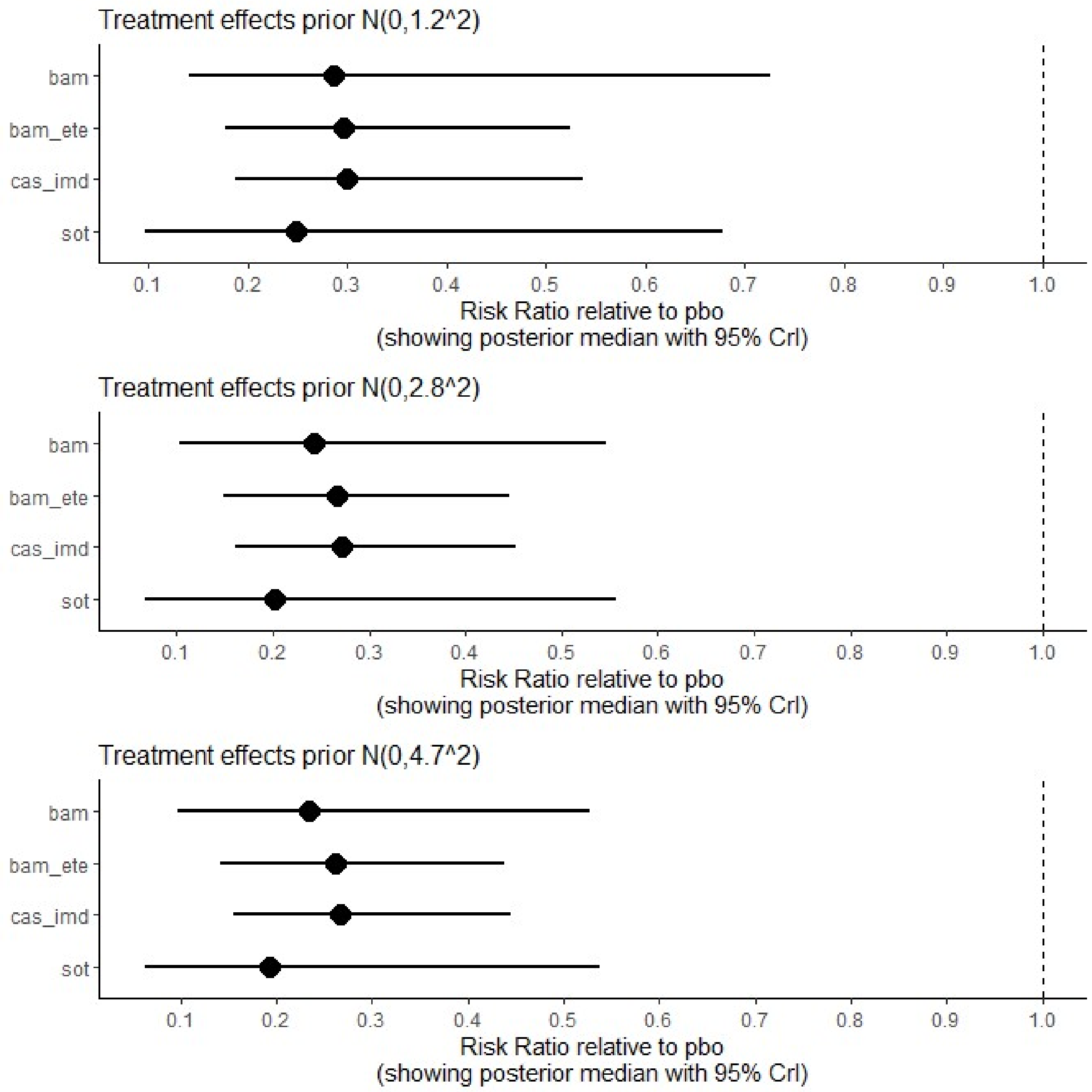

Figure 4.31: Sensitivity analysis using alternative prior distributions for the treatment effect parameter (log relative risk), for the outcome of Hospitalisation. Top: Efficacy prior N(0,1.2^2) (more informative), middle: Efficacy prior N(0,2.8^2) (base case), bottom: Efficacy prior N(0,4.7^2) (less informative)

5.1.3 Invasive Ventilation

Table 4.15: NMA sensitivity analysis results for the outcome of Invasive Ventilation, using a prior distribution of N(0,1.2^2) for the treatment effect parameters.

	pbo	cas_imd	sot
pbo	pbo	0.32 (0.08 to 1.14)	0.26 (0.04 to 1.34)
cas_imd	3.12 (0.88 to 12.41)	cas_imd	0.81 (0.09 to 6.78)

	pbo	cas_imd	sot
sot	3.90 (0.74 to 22.99)	1.24 (0.15 to 10.88)	sot

Table 4.16: NMA sensitivity analysis results for the outcome of Invasive Ventilation, using a prior distribution of N(0,2.8^2) for the treatment effect parameters.

	pbo	cas_imd	sot
pbo	pbo	0.20 (0.03 to 0.89)	0.05 (0.00 to 0.70)
cas_imd	4.95 (1.13 to 31.27)	cas_imd	0.24 (0.00 to 6.34)
sot	20.33 (1.43 to 1015.15)	4.13 (0.16 to 272.44)	sot

Table 4.17: NMA sensitivity analysis results for the outcome of Invasive Ventilation, using a prior distribution of N(0,4.7^2) for the treatment effect parameters.

	pbo	cas_imd	sot
pbo	pbo	0.18 (0.02 to 0.87)	0.01 (0.00 to 0.44)
cas_imd	5.46 (1.15 to 41.58)	cas_imd	0.07 (0.00 to 4.19)
sot	83.10 (2.27 to 59406.52)	15.23 (0.24 to 12080.28)	sot

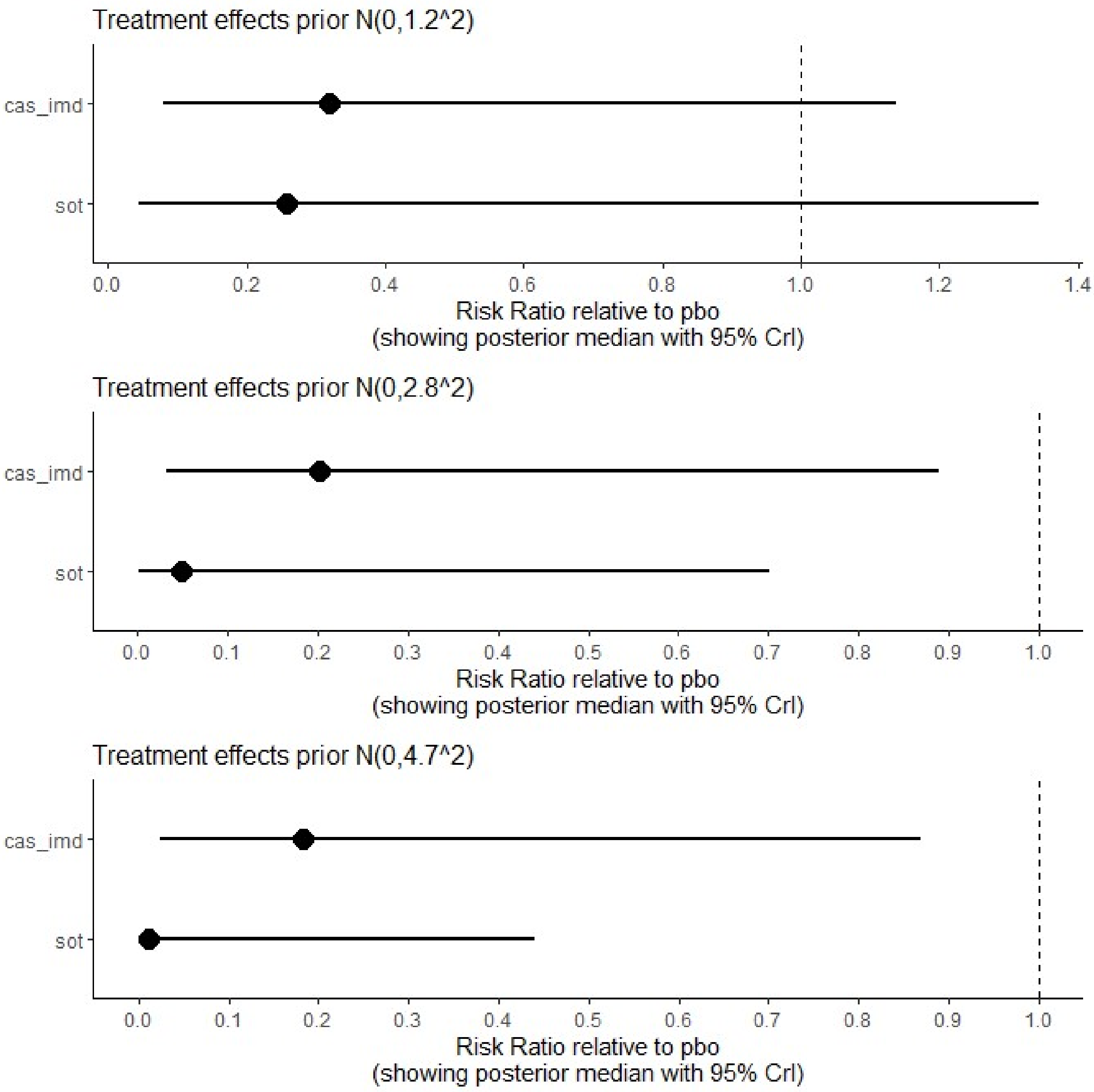

Figure 4.32: Sensitivity analysis using alternative prior distributions for the treatment effect parameter (log relative risk), for the outcome of Invasive Ventilation. Top: Efficacy prior N(0,1.2^2) (more informative), middle: Efficacy prior N(0,2.8^2) (base case), bottom: Efficacy prior N(0,4.7^2) (less informative)

5.1.4 ICU Admission

Table 4.18: NMA sensitivity analysis results for the outcome of ICU Admission, using a prior distribution of N(0,1.2^2) for the treatment effect parameters.

	pbo	cas_imd	sot
pbo	pbo	0.38 (0.15 to 1.02)	0.16 (0.03 to 0.74)
cas_imd	2.63 (0.98 to 6.46)	cas_imd	0.40 (0.06 to 2.42)

	pbo	cas_imd	sot
sot	6.41 (1.35 to 34.70)	2.47 (0.41 to 16.37)	sot

Table 4.19: NMA sensitivity analysis results for the outcome of ICU Admission, using a prior distribution of N(0,2.8^2) for the treatment effect parameters.

	pbo	cas_imd	sot
pbo	pbo	0.33 (0.12 to 0.87)	0.03 (0.00 to 0.30)
cas_imd	2.99 (1.14 to 8.24)	cas_imd	0.08 (0.00 to 1.12)
sot	37.97 (3.37 to 1670.79)	12.78 (0.89 to 628.92)	sot

Table 4.20: NMA sensitivity analysis results for the outcome of ICU Admission, using a prior distribution of N(0,4.7^2) for the treatment effect parameters.

	pbo	cas_imd	sot
pbo	pbo	0.33 (0.12 to 0.87)	0.01 (0.00 to 0.20)
cas_imd	3.04 (1.15 to 8.46)	cas_imd	0.02 (0.00 to 0.73)
sot	152.45 (5.02 to 101024.47)	50.44 (1.37 to 34553.72)	sot

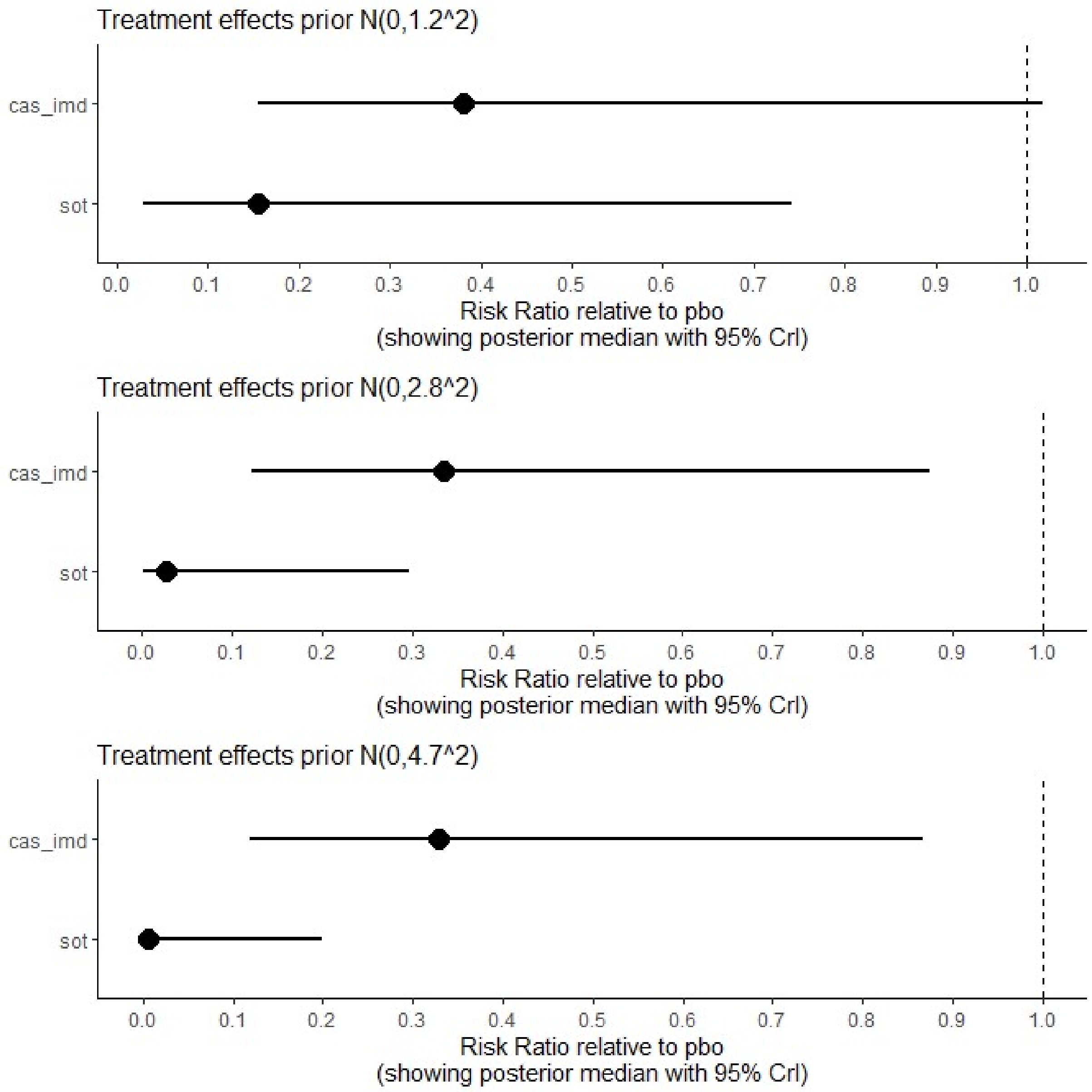

Figure 4.33: Sensitivity analysis using alternative prior distributions for the treatment effect parameter (log relative risk), for the outcome of ICU Admission. Top: Efficacy prior N(0,1.2^2) (more informative), middle: Efficacy prior N(0,2.8^2) (base case), bottom: Efficacy prior N(0,4.7^2) (less informative)

5.1.5 Infusion-related AEs

Table 4.21: NMA sensitivity analysis results for the outcome of Infusion-related AEs, using a prior distribution of N(0,1.2^2) for the treatment effect parameters.

	pbo	bam	bam_ete	cas_imd	sot
pbo	pbo	0.88 (0.14 to 4.42)	1.63 (0.39 to 6.58)	1.28 (0.36 to 4.65)	0.90 (0.27 to 2.96)

	pbo	bam	bam_ete	cas_imd	sot
bam	1.14 (0.23 to 7.12)	bam	1.82 (0.36 to 12.59)	1.44 (0.30 to 9.57)	1.03 (0.14 to 8.94)
bam_ete	0.62 (0.15 to 2.54)	0.55 (0.08 to 2.80)	bam_ete	0.78 (0.26 to 2.46)	0.55 (0.09 to 3.52)
cas_imd	0.78 (0.22 to 2.80)	0.70 (0.10 to 3.38)	1.28 (0.41 to 3.80)	cas_imd	0.70 (0.12 to 4.00)
sot	1.12 (0.34 to 3.69)	0.97 (0.11 to 7.40)	1.83 (0.28 to 11.31)	1.44 (0.25 to 8.23)	sot

Table 4.22: NMA sensitivity analysis results for the outcome of Infusion-related AEs, using a prior distribution of N(0,2.8^2) for the treatment effect parameters.

	pbo	bam	bam_ete	cas_imd	sot
pbo	pbo	1.00 (0.05 to 13.92)	2.49 (0.28 to 24.47)	1.81 (0.26 to 15.06)	0.86 (0.21 to 3.53)
bam	1.00 (0.07 to 20.85)	bam	2.35 (0.33 to 41.04)	1.74 (0.25 to 30.84)	0.86 (0.04 to 25.16)
bam_ete	0.40 (0.04 to 3.62)	0.42 (0.02 to 3.01)	bam_ete	0.74 (0.21 to 2.63)	0.34 (0.02 to 4.83)
cas_imd	0.55 (0.07 to 3.84)	0.58 (0.03 to 4.01)	1.36 (0.38 to 4.68)	cas_imd	0.47 (0.04 to 5.22)
sot	1.16 (0.28 to 4.83)	1.16 (0.04 to 23.38)	2.90 (0.21 to 42.23)	2.13 (0.19 to 26.81)	sot

Table 4.23: NMA sensitivity analysis results for the outcome of Infusion-related AEs, using a prior distribution of N(0,4.7^2) for the treatment effect parameters.

	pbo	bam	bam_ete	cas_imd	sot
pbo	pbo	1.19 (0.03 to 31.77)	3.01 (0.25 to 56.06)	2.16 (0.24 to 33.84)	0.86 (0.20 to 3.71)
bam	0.84 (0.03 to 28.90)	bam	2.36 (0.32 to 51.36)	1.72 (0.23 to 37.83)	0.72 (0.02 to 32.38)
bam_ete	0.33 (0.02 to 4.06)	0.42 (0.02 to 3.11)	bam_ete	0.73 (0.20 to 2.69)	0.28 (0.01 to 5.16)
cas_imd	0.46 (0.03 to 4.12)	0.58 (0.03 to 4.32)	1.37 (0.37 to 4.94)	cas_imd	0.39 (0.02 to 5.59)
sot	1.16 (0.27 to 5.12)	1.39 (0.03 to 50.31)	3.53 (0.19 to 90.40)	2.54 (0.18 to 55.10)	sot

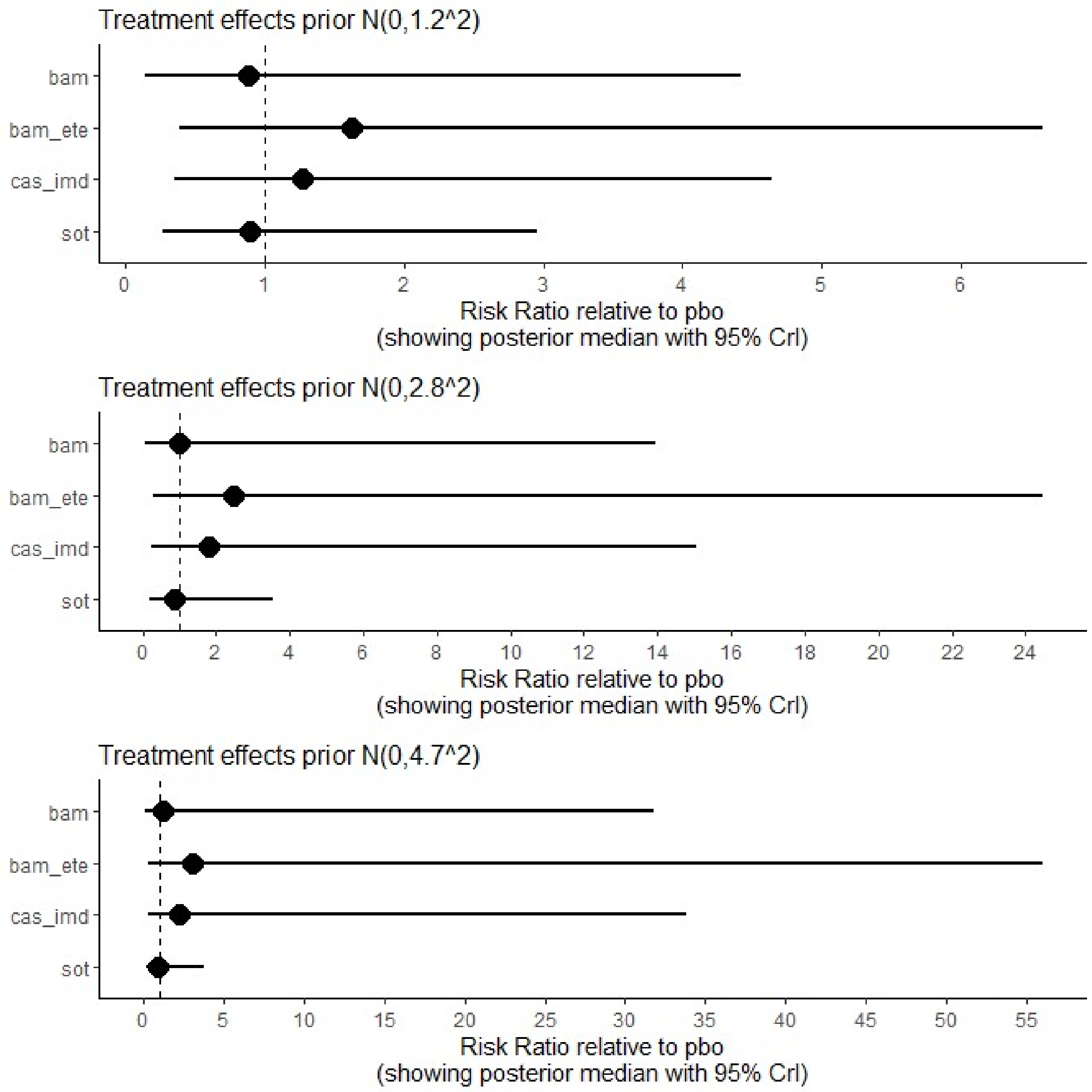

Figure 4.34: Sensitivity analysis using alternative prior distributions for the treatment effect parameter (log relative risk), for the outcome of Infusion-related AEs. Top: Efficacy prior N(0,1.2^2) (more informative), middle: Efficacy prior N(0,2.8^2) (base case), bottom: Efficacy prior N(0,4.7^2) (less informative)

5.1.6 Serious AEs

Table 4.24: NMA sensitivity analysis results for the outcome of Serious AEs, using a prior distribution of N(0,1.2^2) for the treatment effect parameters.

	pbo	bam	bam_ete	cas_imd	sot
pbo	pbo	1.16 (0.19 to 6.11)	0.64 (0.21 to 1.94)	0.45 (0.21 to 1.58)	0.40 (0.15 to 1.42)

	pbo	bam	bam_ete	cas_imd	sot
bam	0.86 (0.16 to 5.40)	bam	0.55 (0.09 to 4.12)	0.41 (0.07 to 3.12)	0.36 (0.05 to 3.15)
bam_ete	1.56 (0.51 to 4.71)	1.82 (0.24 to 11.39)	bam_ete	0.73 (0.21 to 3.35)	0.63 (0.14 to 3.40)
cas_imd	2.23 (0.63 to 4.70)	2.45 (0.32 to 13.62)	1.38 (0.30 to 4.66)	cas_imd	0.89 (0.19 to 3.35)
sot	2.48 (0.71 to 6.86)	2.82 (0.32 to 19.73)	1.58 (0.29 to 6.95)	1.12 (0.30 to 5.40)	sot

Table 4.25: NMA sensitivity analysis results for the outcome of Serious AEs, using a prior distribution of N(0,2.8^2) for the treatment effect parameters.

	pbo	bam	bam_ete	cas_imd	sot
pbo	pbo	1.12 (0.07 to 10.86)	0.58 (0.15 to 2.07)	0.41 (0.17 to 1.69)	0.34 (0.10 to 1.34)
bam	0.89 (0.09 to 13.92)	bam	0.51 (0.05 to 8.22)	0.39 (0.04 to 6.60)	0.31 (0.02 to 6.29)
bam_ete	1.72 (0.48 to 6.64)	1.96 (0.12 to 19.65)	bam_ete	0.74 (0.20 to 4.18)	0.60 (0.10 to 4.09)
cas_imd	2.44 (0.59 to 5.77)	2.58 (0.15 to 22.58)	1.36 (0.24 to 5.03)	cas_imd	0.83 (0.12 to 3.73)
sot	2.90 (0.75 to 10.51)	3.19 (0.16 to 44.39)	1.68 (0.24 to 9.98)	1.20 (0.27 to 8.53)	sot

Table 4.26: NMA sensitivity analysis results for the outcome of Serious AEs, using a prior distribution of N(0,4.7^2) for the treatment effect parameters.

	pbo	bam	bam_ete	cas_imd	sot
pbo	pbo	1.02 (0.03 to 12.31)	0.56 (0.14 to 2.10)	0.40 (0.16 to 1.73)	0.33 (0.08 to 1.33)
bam	0.98 (0.08 to 29.36)	bam	0.55 (0.05 to 16.26)	0.42 (0.04 to 12.92)	0.33 (0.02 to 12.29)
bam_ete	1.77 (0.48 to 7.28)	1.82 (0.06 to 22.17)	bam_ete	0.75 (0.19 to 4.51)	0.59 (0.09 to 4.31)
cas_imd	2.48 (0.58 to 6.08)	2.38 (0.08 to 24.13)	1.34 (0.22 to 5.14)	cas_imd	0.82 (0.10 to 3.85)
sot	2.99 (0.75 to 11.84)	3.00 (0.08 to 53.02)	1.69 (0.23 to 11.05)	1.22 (0.26 to 9.85)	sot

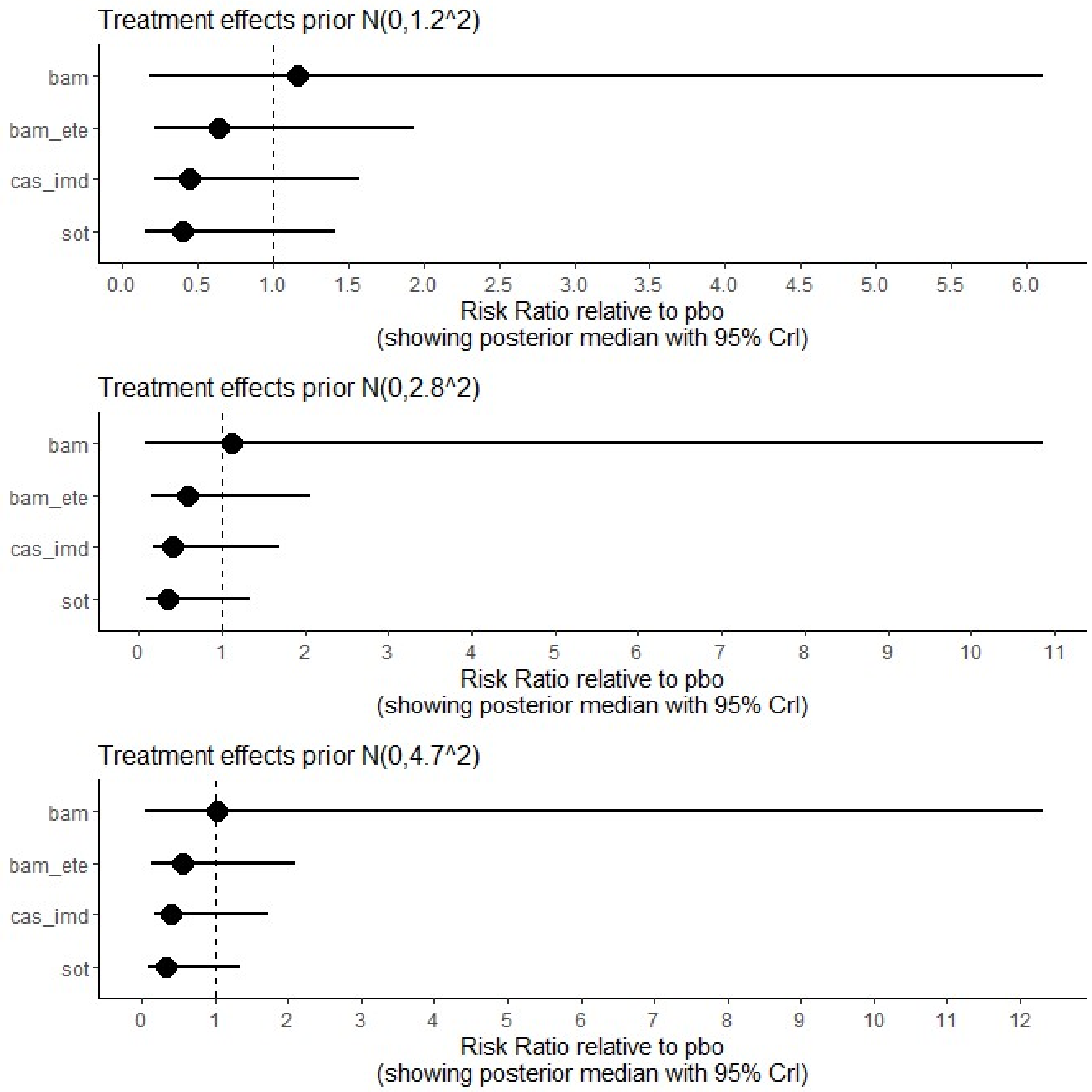

Figure 4.35: Sensitivity analysis using alternative prior distributions for the treatment effect parameter (log relative risk), for the outcome of Serious AEs. Top: Efficacy prior N(0,1.2^2) (more informative), middle: Efficacy prior N(0,2.8^2) (base case), bottom: Efficacy prior N(0,4.7^2) (less informative)

```
       used  (Mb) gc trigger   (Mb)  max used   (Mb)
```

Ncells 2753765 147.1 7652530 408.7 7652530 408.7 Vcells 52963674 404.1 768366254 5862.2 572533688 4368.1

5.2 Fixed versus random effects

5.2.1 Mortality

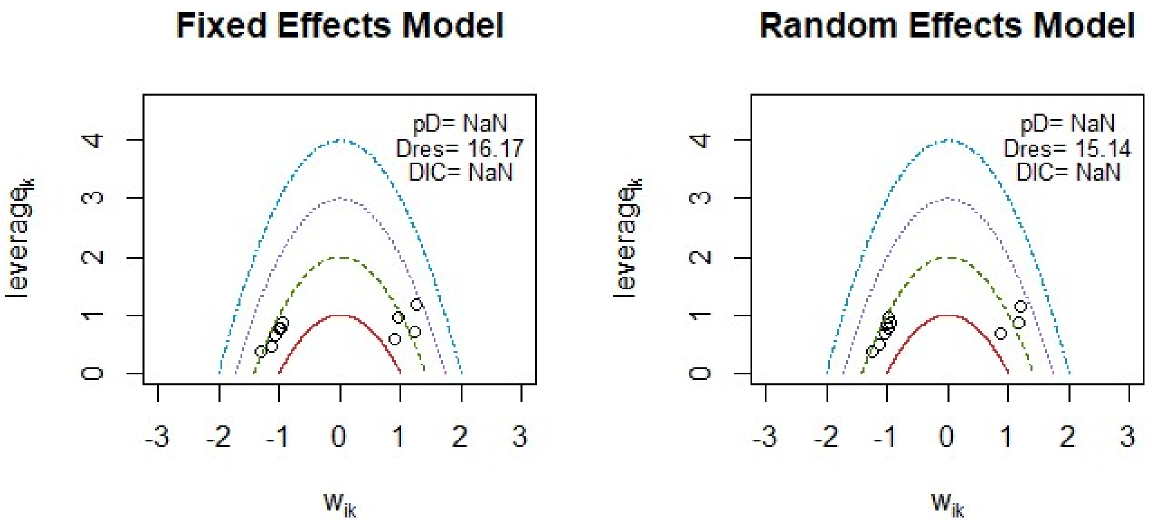

Figure 4.36: Comparison of the fit of fixed and random effects models for the outcome of Mortality

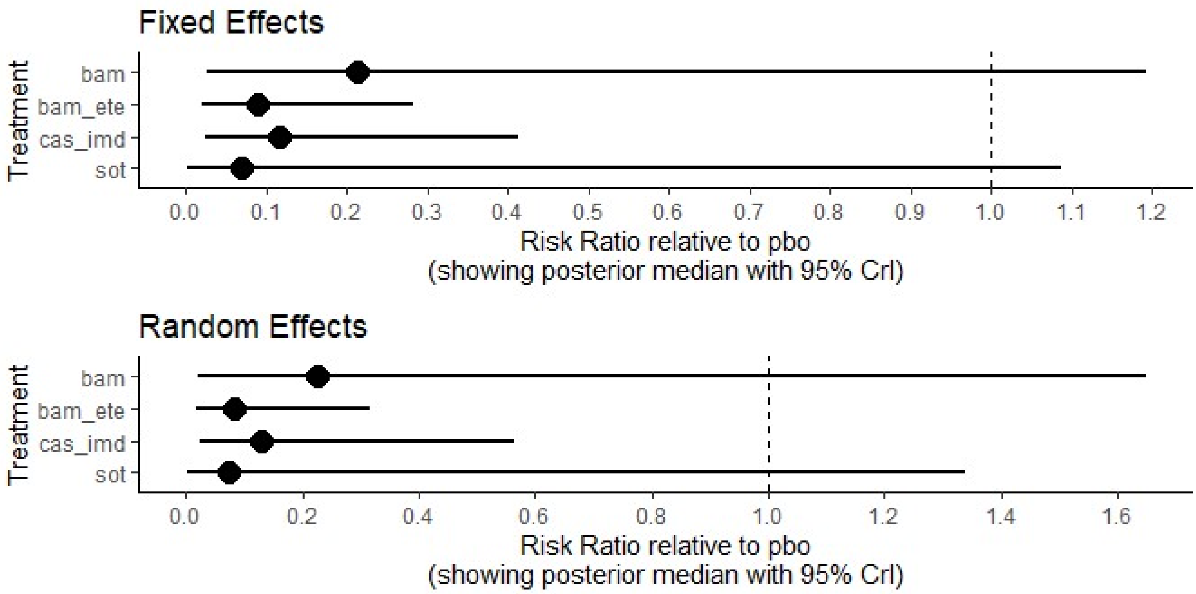

Figure 4.37: Comparison of estimated treatment effects (relative risks) versus placebo for the outcome of Mortality

5.2.2 Hospitalisation

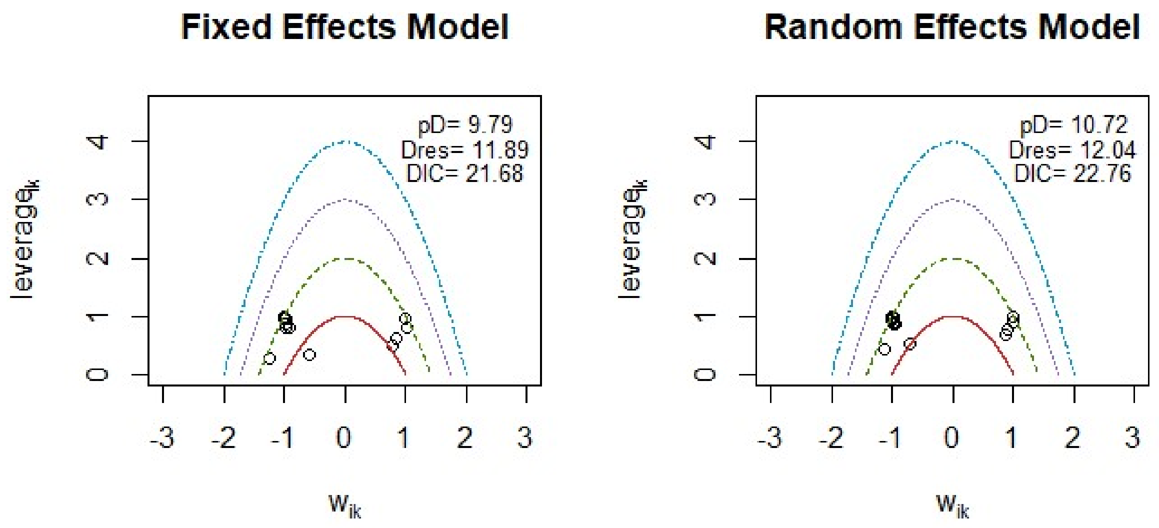

Figure 4.38: Comparison of the fit of fixed and random effects models for the outcome of Hospitalisation

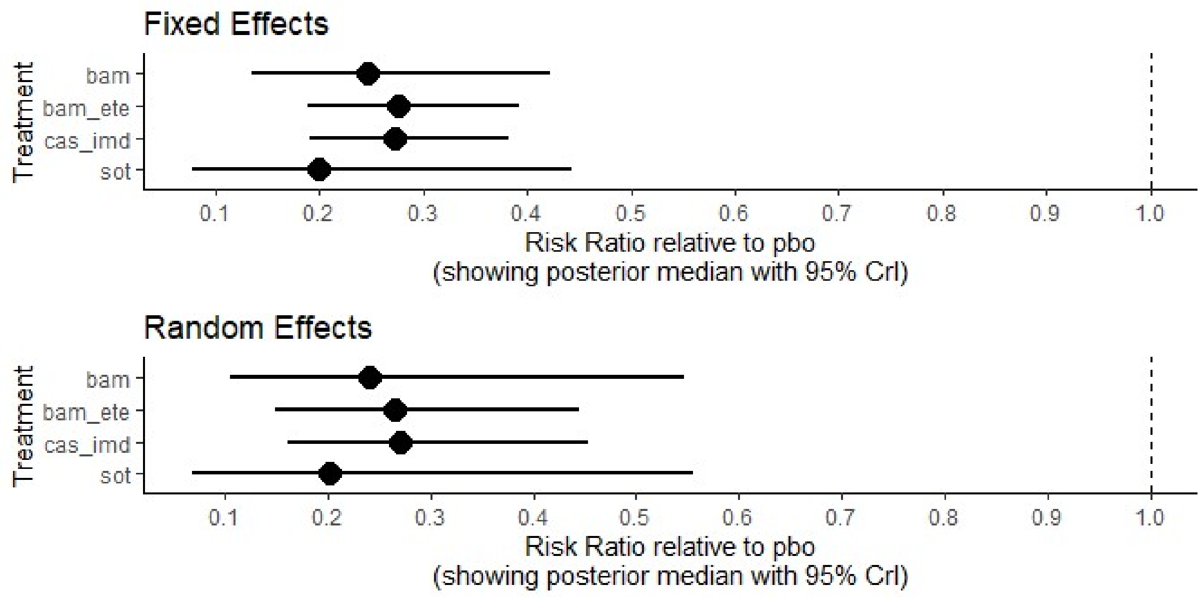

Figure 4.39: Comparison of estimated treatment effects (relative risks) versus placebo for the outcome of Hospitalisation

5.2.3 Invasive Ventilation

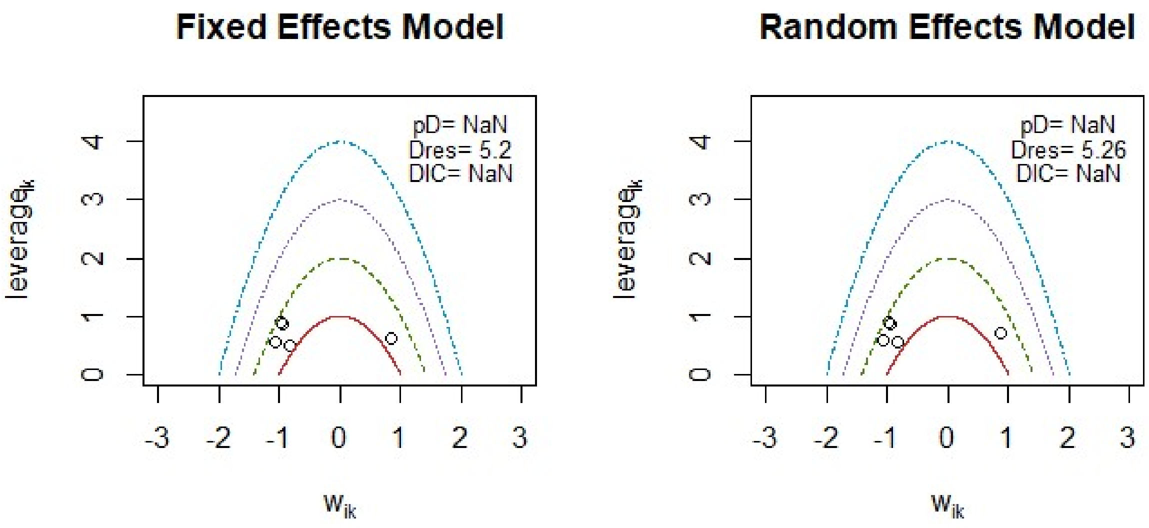

Figure 4.40: Comparison of the fit of fixed and random effects models for the outcome of Invasive Ventilation

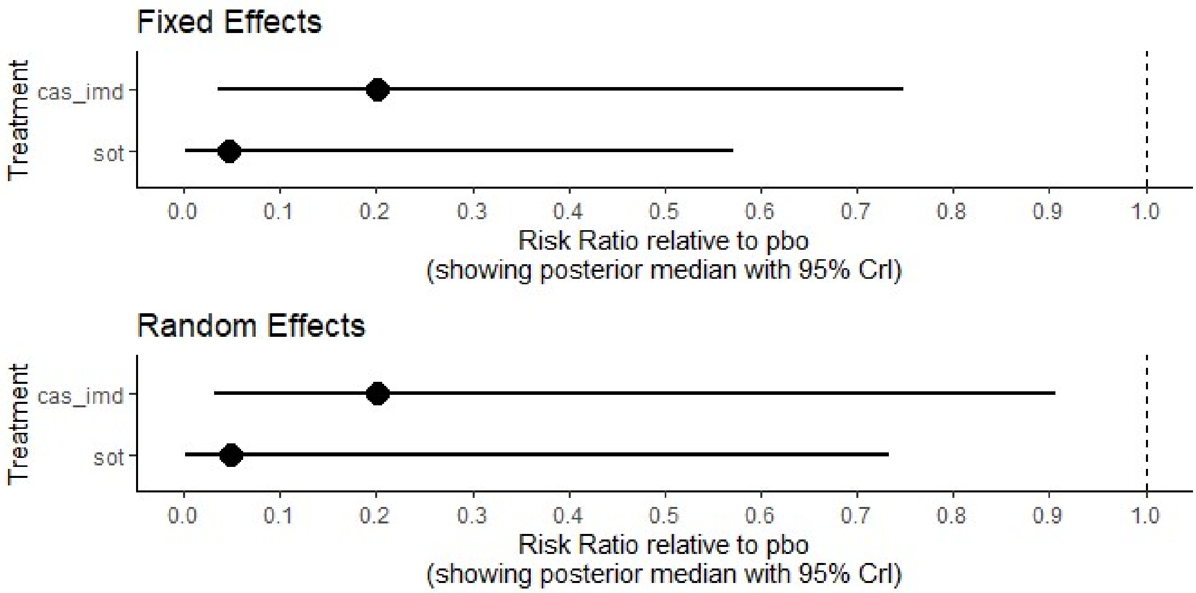

Figure 4.41: Comparison of estimated treatment effects (relative risks) versus placebo for the outcome of Invasive Ventilation

5.2.4 ICU Admission

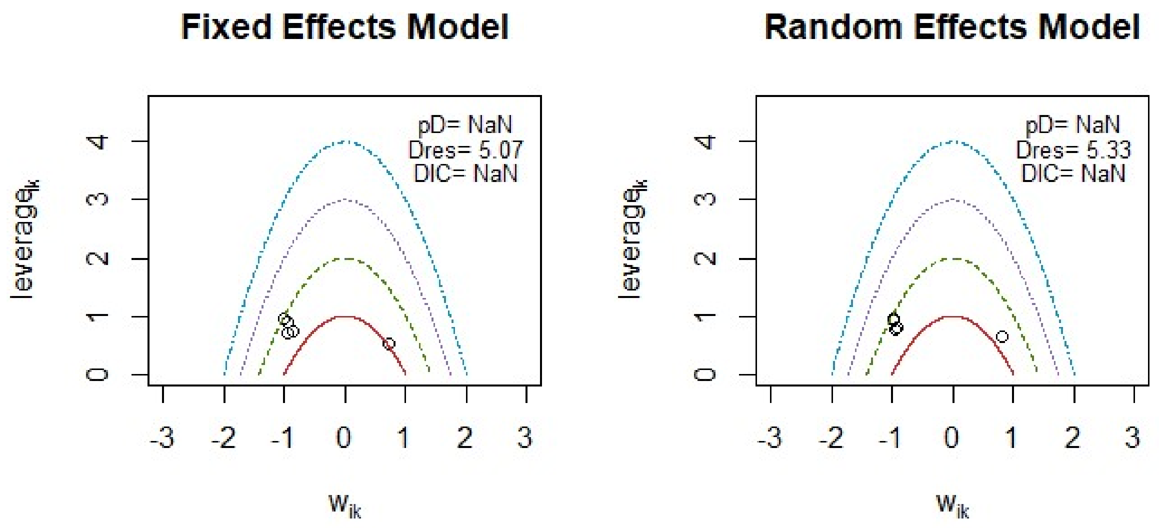

Figure 4.42: Comparison of the fit of fixed and random effects models for the outcome of ICU Admission

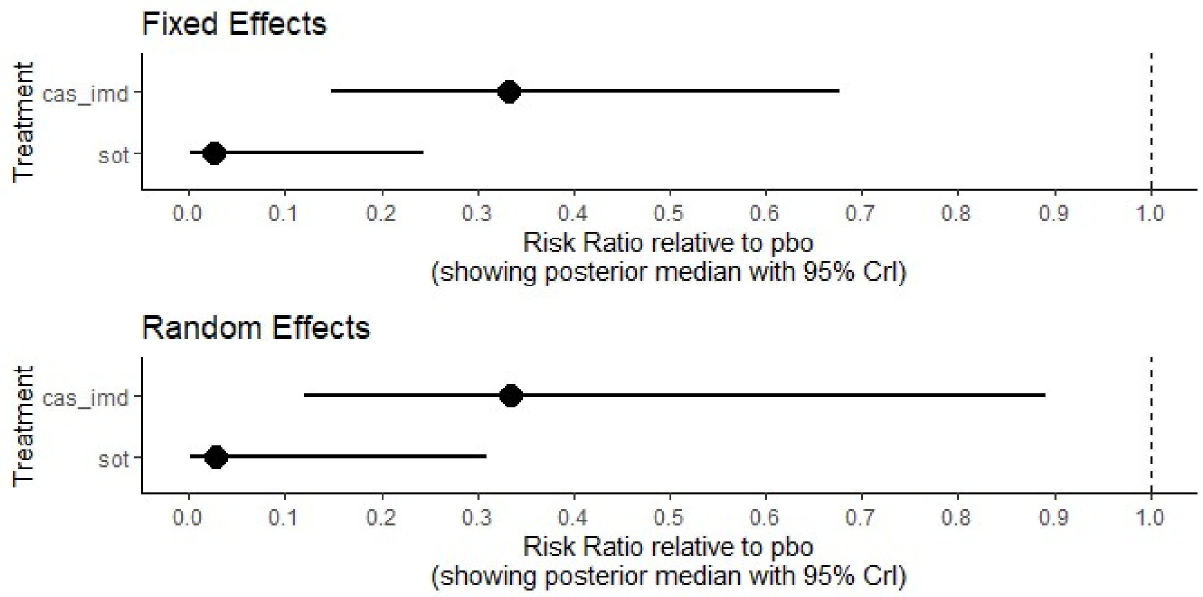

Figure 4.43: Comparison of estimated treatment effects (relative risks) versus placebo for the outcome of ICU Admission

5.2.5 Infusion-related AEs

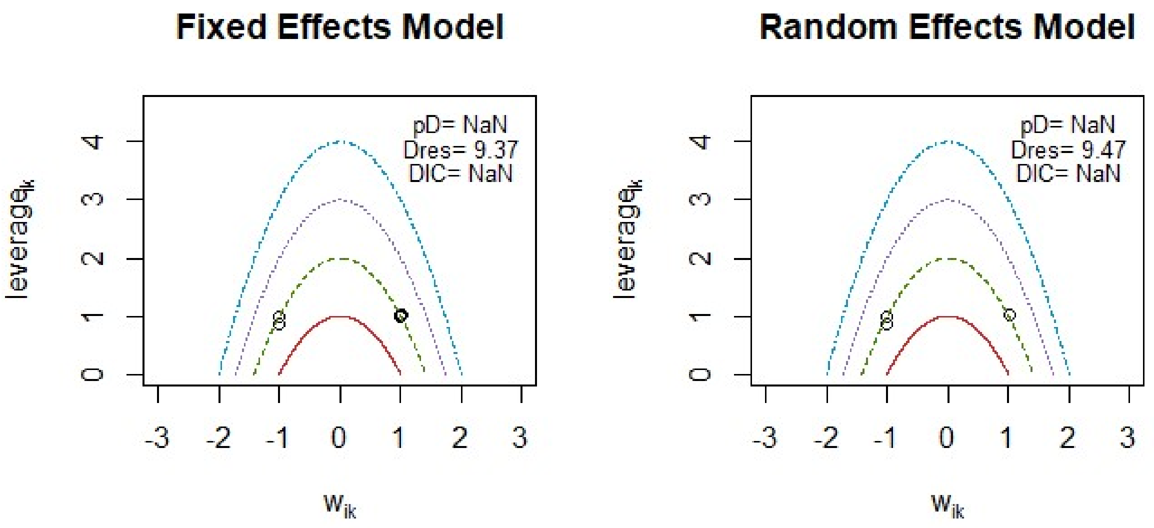

Figure 4.44: Comparison of the fit of fixed and random effects models for the outcome of Infusion-related AEs

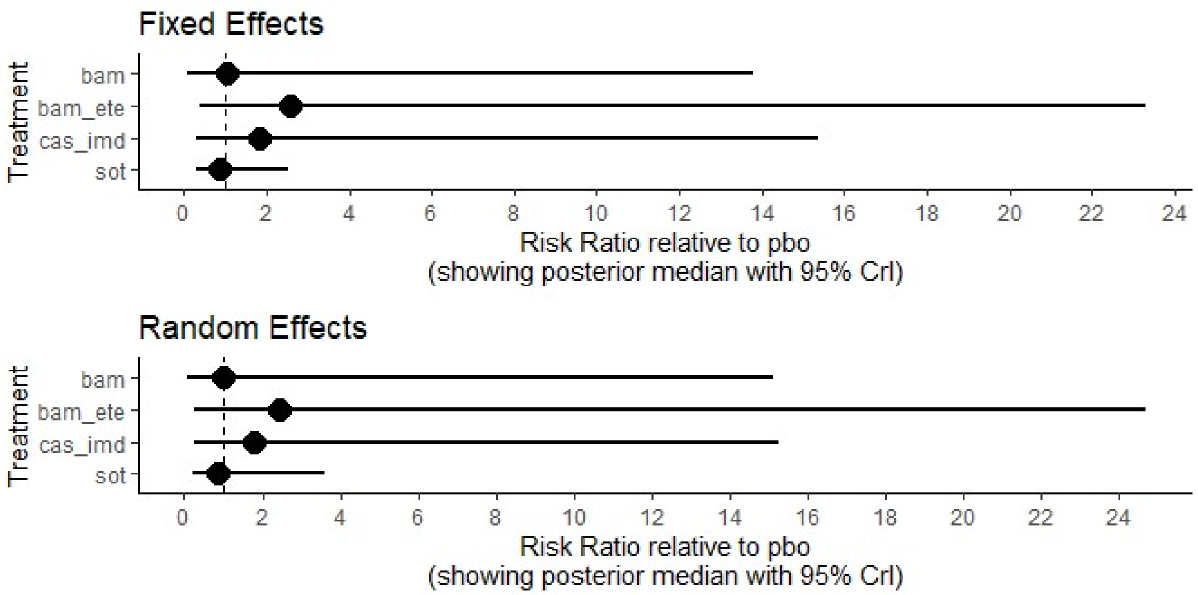

Figure 4.45: Comparison of estimated treatment effects (relative risks) versus placebo for the outcome of Infusion-related AEs

5.2.6 Serious AEs

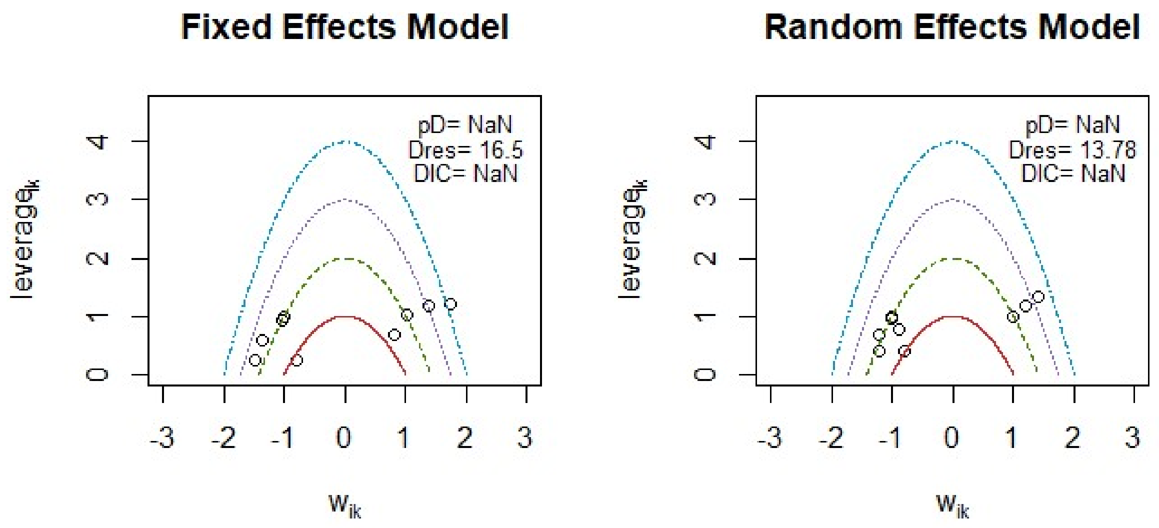

Figure 4.46: Comparison of the fit of fixed and random effects models for the outcome of Serious AEs

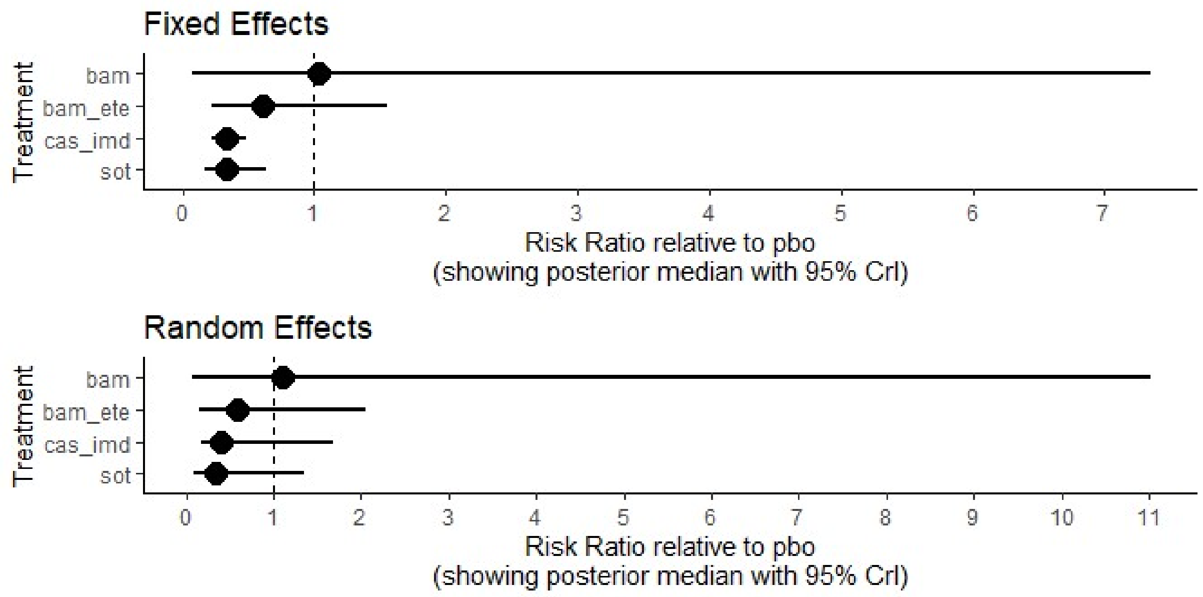

Figure 4.47: Comparison of estimated treatment effects (relative risks) versus placebo for the outcome of Serious AEs

5.3 Heterogeneity Prior

For all outcomes we examined the sensitivity of the results to the choice of prior distribution for the heterogeneity parameter (between trial standard deviation). The following distributions were examined:

1. Base case: half-normal(0.5)
2. Uniform(0,5)
3. Informative priors from Turner et al. (2012) for the between trial *variance*: log-normal(-4.06,1.45^2) for mortality and log-normal(-3.02, 1.85^2) for other outcomes
4. Alternative weakly-informative prior: Half-Cauchy(0.5) Röver et al. (2021)

5.3.1 Mortality

Table 4.27: NMA sensitivity analysis results for the outcome of Mortality using the prior distribution HN(0.5) (base case) for the heterogeneity parameter

	pbo	bam	bam_ete	cas_imd	sot
pbo	pbo	0.22 (0.02 to 1.63)	0.08 (0.02 to 0.32)	0.13 (0.02 to 0.57)	0.07 (0.00 to 1.27)
bam	4.51 (0.61 to 42.04)	bam	0.37 (0.06 to 2.67)	0.56 (0.11 to 4.24)	0.31 (0.00 to 12.13)
bam_ete	11.87 (3.15 to 61.90)	2.73 (0.37 to 16.85)	bam_ete	1.51 (0.43 to 7.03)	0.84 (0.01 to 23.80)
cas_imd	7.73 (1.74 to 41.24)	1.79 (0.24 to 9.48)	0.66 (0.14 to 2.32)	cas_imd	0.55 (0.01 to 15.18)
sot	14.13 (0.79 to 890.81)	3.24 (0.08 to 321.30)	1.18 (0.04 to 95.47)	1.83 (0.07 to 155.52)	sot

Table 4.28: NMA sensitivity analysis results for the outcome of Mortality using the prior distribution Unif(0,5) for the heterogeneity parameter

	pbo	bam	bam_ete	cas_imd	sot
pbo	pbo	0.32 (0.01 to 16.04)	0.08 (0.00 to 1.29)	0.19 (0.02 to 4.30)	0.10 (0.00 to 7.61)
bam	3.12 (0.06 to 78.58)	bam	0.25 (0.00 to 7.98)	0.60 (0.01 to 27.17)	0.30 (0.00 to 61.29)
bam_ete	12.89 (0.77 to 228.23)	4.08 (0.13 to 305.55)	bam_ete	2.39 (0.13 to 114.77)	1.32 (0.01 to 247.11)
cas_imd	5.18 (0.23 to 54.33)	1.66 (0.04 to 72.25)	0.42 (0.01 to 7.58)	cas_imd	0.50 (0.00 to 60.55)
sot	9.54 (0.13 to 804.06)	3.29 (0.02 to 911.74)	0.76 (0.00 to 121.18)	2.00 (0.02 to 344.21)	sot

Table 4.29: NMA sensitivity analysis results for the outcome of Mortality using the prior distribution from Turner et al. for the heterogeneity parameter

	pbo	bam	bam_ete	cas_imd	sot
pbo	pbo	0.22 (0.02 to 1.27)	0.09 (0.02 to 0.28)	0.12 (0.02 to 0.44)	0.07 (0.00 to 1.11)
bam	4.57 (0.79 to 40.33)	bam	0.40 (0.09 to 2.50)	0.53 (0.13 to 3.57)	0.29 (0.00 to 11.30)
bam_ete	11.06 (3.52 to 49.67)	2.51 (0.40 to 11.43)	bam_ete	1.34 (0.46 to 4.26)	0.74 (0.01 to 17.93)
cas_imd	8.13 (2.25 to 41.30)	1.88 (0.28 to 7.95)	0.75 (0.23 to 2.18)	cas_imd	0.54 (0.01 to 14.82)
sot	14.69 (0.90 to 1174.07)	3.41 (0.09 to 301.53)	1.36 (0.06 to 108.70)	1.84 (0.07 to 155.82)	sot

Table 4.30: NMA sensitivity analysis results for the outcome of Mortality using the prior distribution Half-Cauchy(0.5) for the heterogeneity parameter

	pbo	bam	bam_ete	cas_imd	sot
pbo	pbo	0.24 (0.02 to 3.32)	0.08 (0.01 to 0.43)	0.14 (0.02 to 1.09)	0.08 (0.00 to 2.11)
bam	4.24 (0.30 to 50.13)	bam	0.35 (0.02 to 3.34)	0.57 (0.06 to 7.26)	0.31 (0.00 to 20.03)
bam_ete	12.01 (2.31 to 90.09)	2.86 (0.30 to 44.29)	bam_ete	1.62 (0.35 to 19.19)	0.92 (0.01 to 47.58)
cas_imd	7.13 (0.92 to 42.81)	1.74 (0.14 to 15.76)	0.62 (0.05 to 2.85)	cas_imd	0.53 (0.01 to 21.76)
sot	13.18 (0.47 to 863.46)	3.22 (0.05 to 405.23)	1.09 (0.02 to 99.07)	1.90 (0.05 to 193.42)	sot

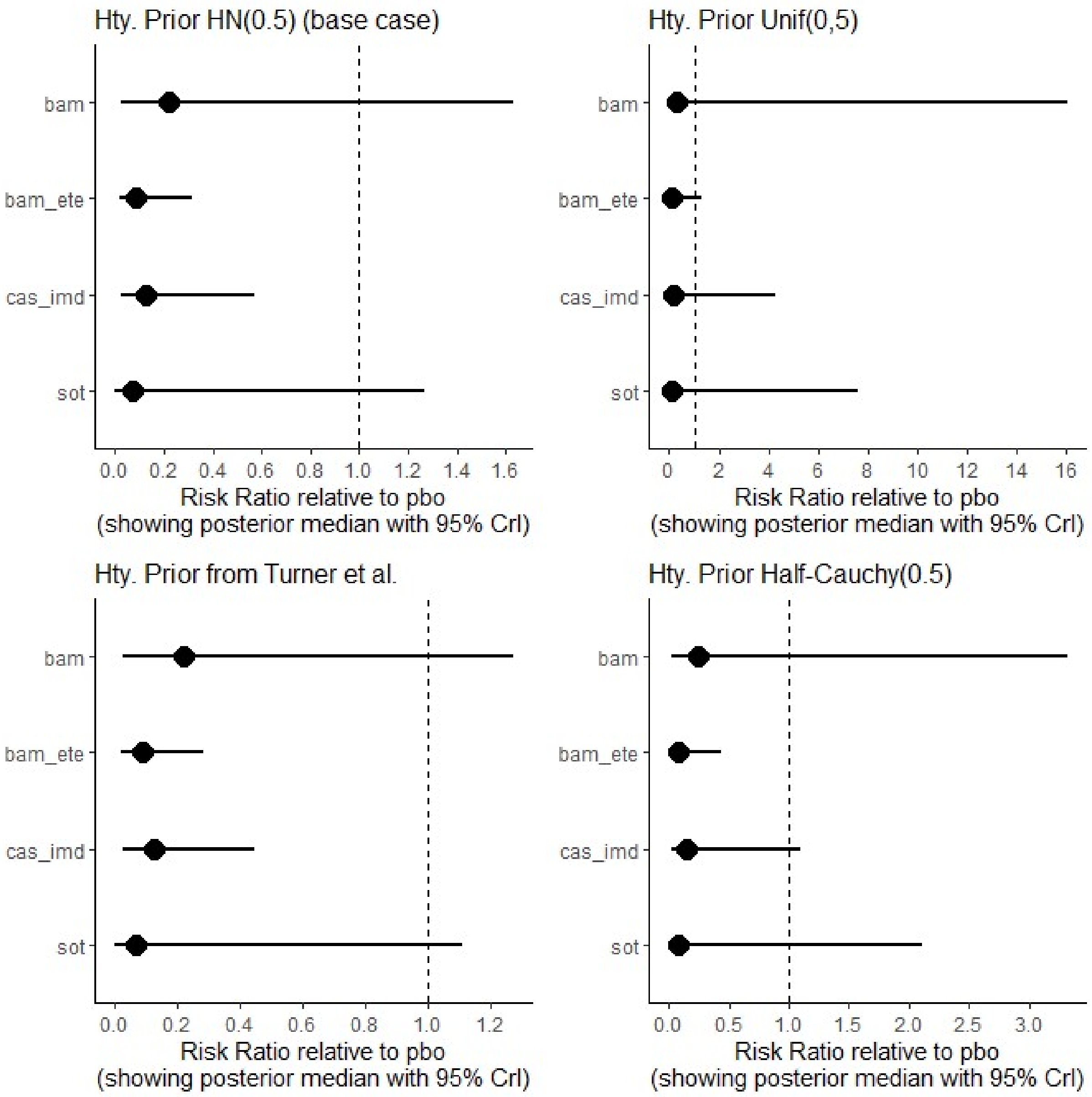

Figure 4.48: Estimated treatment effects for each mAB versus placebo obtained from varying the prior distribution on the heterogeneity parameter sigma, for the outcome of Mortality

5.3.2 Hospitalisation

Table 4.31: NMA sensitivity analysis results for the outcome of Hospitalisation using the prior distribution HN(0.5) (base case) for the heterogeneity parameter

	pbo	bam	bam_ete	cas_imd	sot
pbo	pbo	0.24 (0.10 to 0.56)	0.27 (0.15 to 0.45)	0.27 (0.16 to 0.45)	0.20 (0.07 to 0.55)
bam	4.13 (1.80 to 9.55)	bam	1.10 (0.50 to 2.27)	1.11 (0.53 to 2.37)	0.83 (0.21 to 3.09)

	pbo	bam	bam_ete	cas_imd	sot
bam_ete	3.75 (2.24 to 6.76)	0.91 (0.44 to 1.99)	bam_ete	1.01 (0.61 to 1.83)	0.76 (0.23 to 2.47)
cas_imd	3.69 (2.20 to 6.25)	0.90 (0.42 to 1.87)	0.99 (0.55 to 1.63)	cas_imd	0.74 (0.22 to 2.32)
sot	4.97 (1.81 to 14.99)	1.21 (0.32 to 4.71)	1.32 (0.40 to 4.41)	1.34 (0.43 to 4.50)	sot

Table 4.32: NMA sensitivity analysis results for the outcome of Hospitalisation using the prior distribution Unif(0,5) for the heterogeneity parameter

	pbo	bam	bam_ete	cas_imd	sot
pbo	pbo	0.25 (0.08 to 0.92)	0.27 (0.12 to 0.58)	0.27 (0.13 to 0.62)	0.21 (0.06 to 0.84)
bam	4.08 (1.09 to 12.52)	bam	1.08 (0.31 to 3.04)	1.11 (0.34 to 3.30)	0.84 (0.14 to 4.93)
bam_ete	3.76 (1.72 to 8.34)	0.92 (0.33 to 3.24)	bam_ete	1.02 (0.46 to 2.62)	0.77 (0.18 to 3.95)
cas_imd	3.67 (1.61 to 7.48)	0.90 (0.30 to 2.94)	0.98 (0.38 to 2.18)	cas_imd	0.75 (0.17 to 3.57)
sot	4.87 (1.18 to 17.59)	1.20 (0.20 to 7.06)	1.30 (0.25 to 5.60)	1.33 (0.28 to 5.94)	sot

Table 4.33: NMA sensitivity analysis results for the outcome of Hospitalisation using the prior distribution from Turner et al. for the heterogeneity parameter

	pbo	bam	bam_ete	cas_imd	sot
pbo	pbo	0.24 (0.11 to 0.50)	0.27 (0.16 to 0.43)	0.27 (0.17 to 0.43)	0.20 (0.07 to 0.51)
bam	4.14 (2.00 to 8.99)	bam	1.11 (0.57 to 2.15)	1.12 (0.59 to 2.22)	0.83 (0.24 to 2.80)
bam_ete	3.73 (2.33 to 6.31)	0.90 (0.47 to 1.75)	bam_ete	1.01 (0.66 to 1.64)	0.75 (0.24 to 2.20)
cas_imd	3.69 (2.33 to 5.91)	0.89 (0.45 to 1.69)	0.99 (0.61 to 1.51)	cas_imd	0.74 (0.24 to 2.11)
sot	4.99 (1.96 to 14.12)	1.21 (0.36 to 4.25)	1.34 (0.45 to 4.13)	1.35 (0.47 to 4.18)	sot

Table 4.34: NMA sensitivity analysis results for the outcome of Hospitalisation using the prior distribution Half-Cauchy(0.5) for the heterogeneity parameter

	pbo	bam	bam_ete	cas_imd	sot
pbo	pbo	0.24 (0.10 to 0.57)	0.27 (0.15 to 0.45)	0.27 (0.16 to 0.46)	0.20 (0.07 to 0.57)
bam	4.11 (1.75 to 9.71)	bam	1.09 (0.49 to 2.29)	1.11 (0.52 to 2.40)	0.83 (0.20 to 3.15)
bam_ete	3.74 (2.21 to 6.82)	0.91 (0.44 to 2.05)	bam_ete	1.01 (0.61 to 1.86)	0.76 (0.22 to 2.52)
cas_imd	3.69 (2.17 to 6.29)	0.90 (0.42 to 1.92)	0.99 (0.54 to 1.64)	cas_imd	0.75 (0.22 to 2.37)
sot	4.91 (1.77 to 15.09)	1.20 (0.32 to 4.92)	1.31 (0.40 to 4.48)	1.33 (0.42 to 4.60)	sot

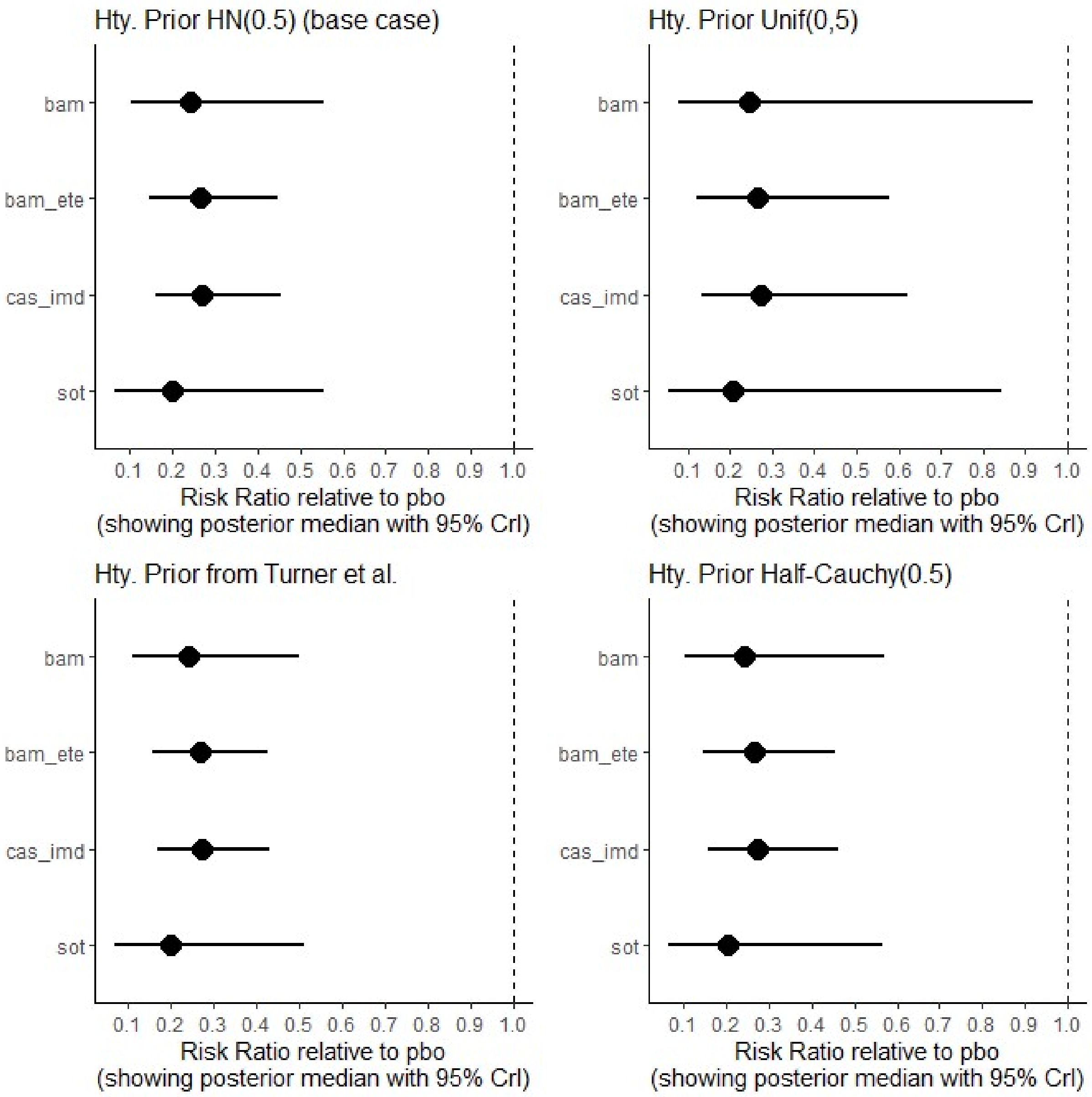

Figure 4.49: Estimated treatment effects for each mAB versus placebo obtained from varying the prior distribution on the heterogeneity parameter sigma, for the outcome of Hospitalisation

5.3.3 Invasive Ventilation

Table 4.35: NMA sensitivity analysis results for the outcome of Invasive Ventilation using the prior distribution HN(0.5) (base case) for the heterogeneity parameter

	pbo	cas_imd	sot
pbo	pbo	0.20 (0.03 to 0.90)	0.05 (0.00 to 0.71)
cas_imd	4.96 (1.11 to 31.58)	cas_imd	0.25 (0.00 to 6.29)
sot	19.97 (1.42 to 1038.79)	4.08 (0.16 to 268.81)	sot

Table 4.36: NMA sensitivity analysis results for the outcome of Invasive Ventilation using the prior distribution Unif(0,5) for the heterogeneity parameter

	pbo	cas_imd	sot
pbo	pbo	0.24 (0.01 to 6.71)	0.08 (0.00 to 6.18)
cas_imd	4.18 (0.15 to 68.02)	cas_imd	0.30 (0.00 to 52.17)
sot	12.89 (0.16 to 961.18)	3.29 (0.02 to 551.16)	sot

Table 4.37: NMA sensitivity analysis results for the outcome of Invasive Ventilation using the prior distribution from Turner et al. for the heterogeneity parameter

	pbo	cas_imd	sot
pbo	pbo	0.20 (0.03 to 0.86)	0.04 (0.00 to 0.66)
cas_imd	4.92 (1.17 to 29.93)	cas_imd	0.22 (0.00 to 5.54)
sot	22.90 (1.53 to 1327.10)	4.62 (0.18 to 329.12)	sot

Table 4.38: NMA sensitivity analysis results for the outcome of Invasive Ventilation using the prior distribution Half-Cauchy(0.5) for the heterogeneity parameter

	pbo	cas_imd	sot
pbo	pbo	0.20 (0.03 to 1.37)	0.05 (0.00 to 1.11)
cas_imd	4.89 (0.73 to 35.43)	cas_imd	0.25 (0.00 to 9.88)
sot	19.05 (0.90 to 979.67)	3.96 (0.10 to 298.83)	sot

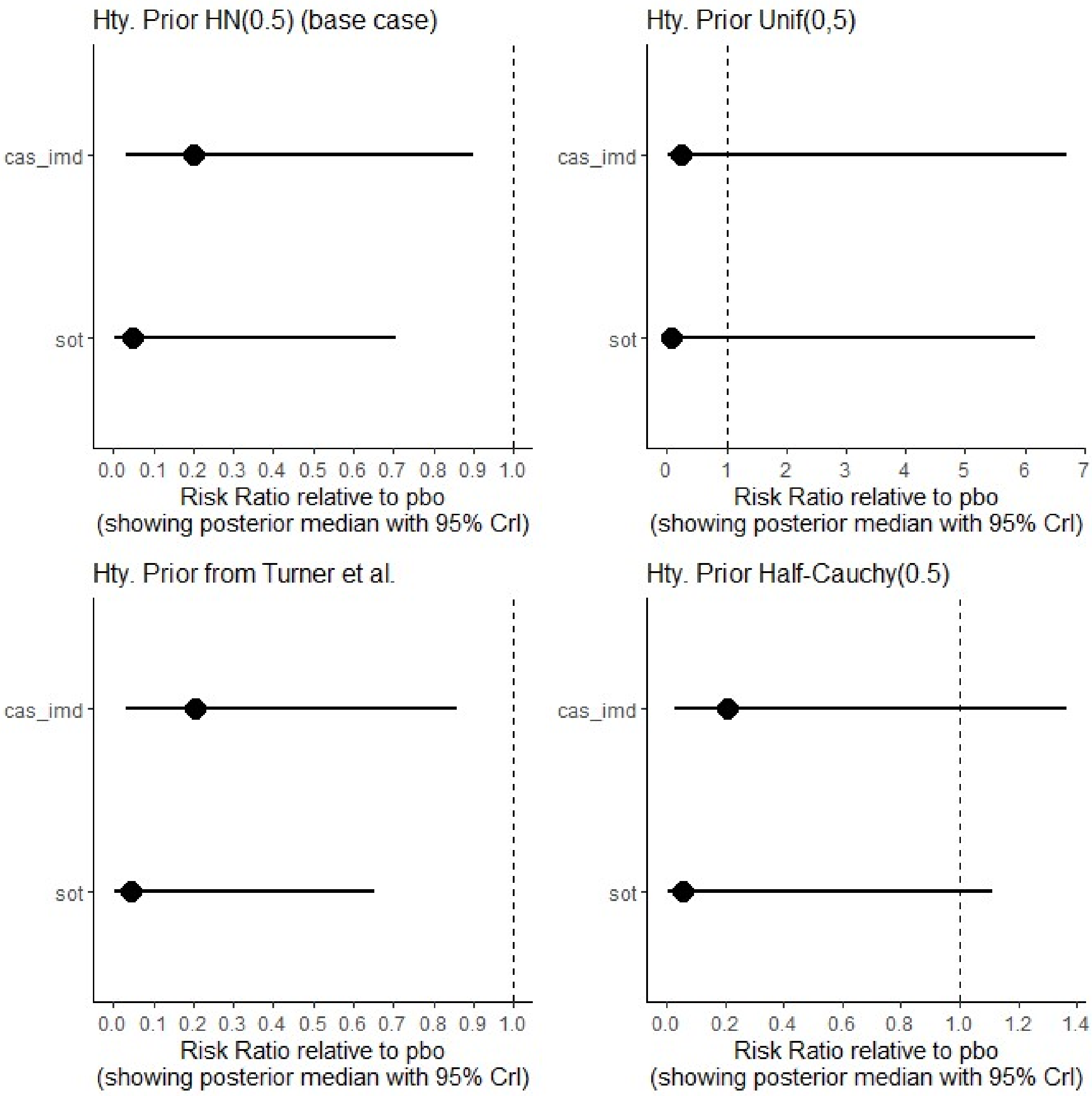

Figure 4.50: Estimated treatment effects for each mAB versus placebo obtained from varying the prior distribution on the heterogeneity parameter sigma, for the outcome of Invasive Ventilation

5.3.4 ICU Admission

Table 4.39: NMA sensitivity analysis results for the outcome of ICU Admission using the prior distribution HN(0.5) (base case) for the heterogeneity parameter

	pbo	cas_imd	sot
pbo	pbo	0.33 (0.12 to 0.88)	0.03 (0.00 to 0.29)
cas_imd	2.99 (1.14 to 8.24)	cas_imd	0.08 (0.00 to 1.08)

	pbo	cas_imd	sot
sot	36.92 (3.44 to 1930.94)	12.43 (0.92 to 691.76)	sot

Table 4.40: NMA sensitivity analysis results for the outcome of ICU Admission using the prior distribution Unif(0,5) for the heterogeneity parameter

	pbo	cas_imd	sot
pbo	pbo	0.36 (0.03 to 7.11)	0.04 (0.00 to 3.61)
cas_imd	2.75 (0.14 to 29.44)	cas_imd	0.11 (0.00 to 17.89)
sot	23.66 (0.28 to 1321.68)	9.40 (0.06 to 912.95)	sot

Table 4.41: NMA sensitivity analysis results for the outcome of ICU Admission using the prior distribution from Turner et al. for the heterogeneity parameter

	pbo	cas_imd	sot
pbo	pbo	0.33 (0.13 to 0.79)	0.03 (0.00 to 0.27)
cas_imd	3.01 (1.27 to 7.56)	cas_imd	0.08 (0.00 to 0.99)
sot	38.76 (3.67 to 1643.79)	12.92 (1.01 to 598.15)	sot

Table 4.42: NMA sensitivity analysis results for the outcome of ICU Admission using the prior distribution Half-Cauchy(0.5) for the heterogeneity parameter

	pbo	cas_imd	sot
pbo	pbo	0.34 (0.10 to 1.26)	0.03 (0.00 to 0.44)
cas_imd	2.95 (0.79 to 9.75)	cas_imd	0.08 (0.00 to 1.66)
sot	35.46 (2.29 to 1510.59)	12.28 (0.60 to 613.12)	sot

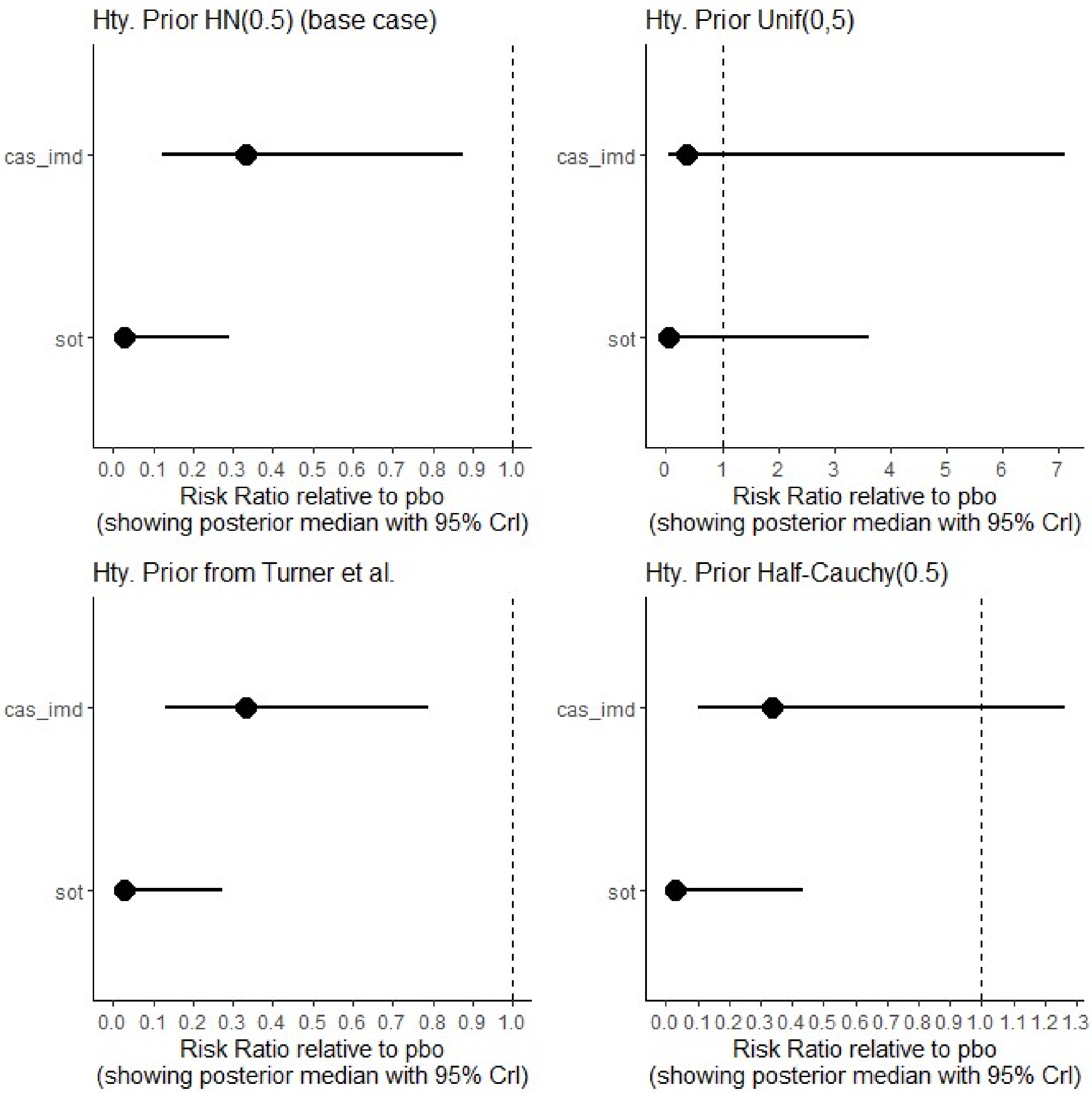

Figure 4.51: Estimated treatment effects for each mAB versus placebo obtained from varying the prior distribution on the heterogeneity parameter sigma, for the outcome of ICU Admission

5.3.5 Infusion-related AEs

Table 4.43: NMA sensitivity analysis results for the outcome of Infusion-related AEs using the prior distribution HN(0.5) (base case) for the heterogeneity parameter

	pbo	bam	bam_ete	cas_imd	sot
pbo	pbo	1.06 (0.05 to 14.74)	2.49 (0.27 to 26.21)	1.82 (0.26 to 16.62)	0.86 (0.21 to 3.54)
bam	0.95 (0.07 to 19.21)	bam	2.24 (0.32 to 39.83)	1.67 (0.25 to 28.08)	0.82 (0.04 to 22.78)

	pbo	bam	bam_ete	cas_imd	sot
bam_ete	0.40 (0.04 to 3.65)	0.45 (0.03 to 3.09)	bam_ete	0.75 (0.21 to 2.68)	0.34 (0.02 to 4.80)
cas_imd	0.55 (0.06 to 3.79)	0.60 (0.04 to 4.06)	1.34 (0.37 to 4.67)	cas_imd	0.46 (0.03 to 5.23)
sot	1.16 (0.28 to 4.81)	1.22 (0.04 to 24.81)	2.92 (0.21 to 44.31)	2.15 (0.19 to 28.63)	sot

Table 4.44: NMA sensitivity analysis results for the outcome of Infusion-related AEs using the prior distribution Unif(0,5) for the heterogeneity parameter

	pbo	bam	bam_ete	cas_imd	sot
pbo	pbo	0.92 (0.02 to 27.44)	2.09 (0.06 to 47.30)	1.67 (0.10 to 26.63)	0.88 (0.04 to 18.97)
bam	1.09 (0.04 to 43.84)	bam	2.20 (0.05 to 101.66)	1.77 (0.05 to 78.65)	0.95 (0.01 to 117.23)
bam_ete	0.48 (0.02 to 15.99)	0.45 (0.01 to 21.04)	bam_ete	0.79 (0.03 to 26.08)	0.41 (0.01 to 49.42)
cas_imd	0.60 (0.04 to 10.50)	0.56 (0.01 to 18.48)	1.27 (0.04 to 29.04)	cas_imd	0.51 (0.01 to 37.16)
sot	1.14 (0.05 to 23.09)	1.05 (0.01 to 89.78)	2.43 (0.02 to 162.64)	1.94 (0.03 to 104.35)	sot

Table 4.45: NMA sensitivity analysis results for the outcome of Infusion-related AEs using the prior distribution from Turner et al. for the heterogeneity parameter

	pbo	bam	bam_ete	cas_imd	sot
pbo	pbo	1.03 (0.05 to 14.65)	2.58 (0.31 to 24.71)	1.88 (0.28 to 15.42)	0.86 (0.23 to 3.25)
bam	0.97 (0.07 to 19.93)	bam	2.38 (0.35 to 43.23)	1.76 (0.27 to 30.06)	0.86 (0.04 to 22.28)
bam_ete	0.39 (0.04 to 3.20)	0.42 (0.02 to 2.83)	bam_ete	0.74 (0.24 to 2.36)	0.34 (0.02 to 4.08)
cas_imd	0.53 (0.06 to 3.54)	0.57 (0.03 to 3.76)	1.36 (0.42 to 4.18)	cas_imd	0.46 (0.04 to 4.54)
sot	1.16 (0.31 to 4.39)	1.16 (0.04 to 23.44)	2.98 (0.24 to 41.48)	2.19 (0.22 to 26.60)	sot

Table 4.46: NMA sensitivity analysis results for the outcome of Infusion-related AEs using the prior distribution Half-Cauchy(0.5) for the heterogeneity parameter

	pbo	bam	bam_ete	cas_imd	sot
pbo	pbo	0.98 (0.04 to 16.76)	2.45 (0.21 to 28.19)	1.82 (0.23 to 17.08)	0.86 (0.15 to 4.90)
bam	1.02 (0.06 to 22.54)	bam	2.37 (0.24 to 44.67)	1.79 (0.19 to 32.82)	0.88 (0.03 to 31.04)
bam_ete	0.41 (0.04 to 4.69)	0.42 (0.02 to 4.18)	bam_ete	0.75 (0.15 to 4.36)	0.35 (0.02 to 7.27)
cas_imd	0.55 (0.06 to 4.34)	0.56 (0.03 to 5.18)	1.34 (0.23 to 6.54)	cas_imd	0.46 (0.03 to 7.10)
sot	1.16 (0.20 to 6.56)	1.14 (0.03 to 31.36)	2.88 (0.14 to 54.82)	2.16 (0.14 to 34.46)	sot

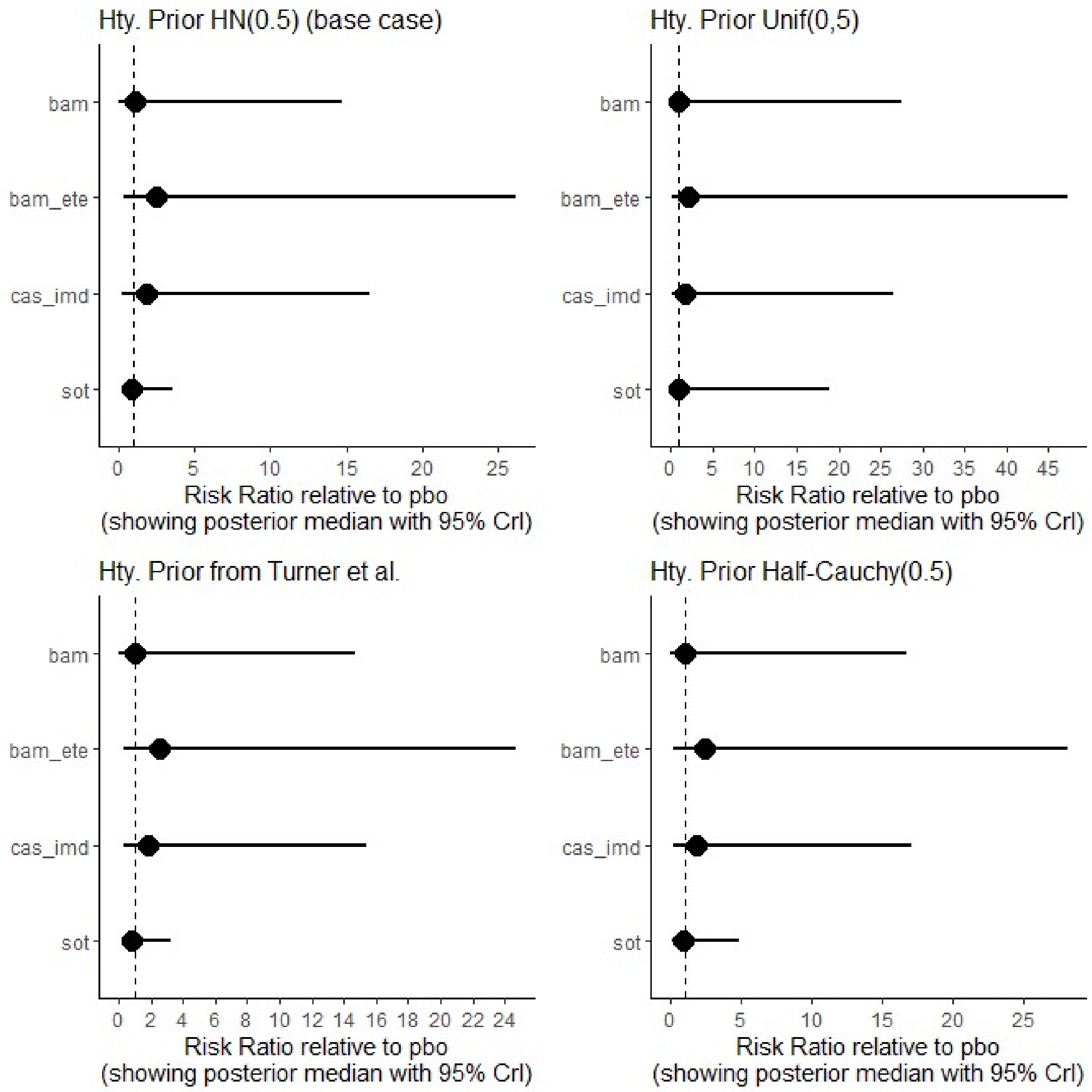

Figure 4.52: Estimated treatment effects for each mAB versus placebo obtained from varying the prior distribution on the heterogeneity parameter sigma, for the outcome of Infusion-related AEs

5.3.6 Serious AEs

Table 4.47: NMA sensitivity analysis results for the outcome of Serious AEs using the prior distribution HN(0.5) (base case) for the heterogeneity parameter

	pbo	bam	bam_ete	cas_imd	sot
pbo	pbo	1.10 (0.06 to 11.20)	0.58 (0.15 to 2.06)	0.41 (0.17 to 1.70)	0.34 (0.09 to 1.35)

	pbo	bam	bam_ete	cas_imd	sot
bam	0.91 (0.09 to 15.79)	bam	0.52 (0.05 to 9.25)	0.40 (0.04 to 7.11)	0.32 (0.02 to 6.99)
bam_ete	1.74 (0.49 to 6.68)	1.91 (0.11 to 20.40)	bam_ete	0.74 (0.19 to 4.25)	0.60 (0.10 to 4.06)
cas_imd	2.43 (0.59 to 5.78)	2.50 (0.14 to 23.09)	1.35 (0.24 to 5.14)	cas_imd	0.83 (0.12 to 3.74)
sot	2.91 (0.74 to 10.58)	3.14 (0.14 to 45.41)	1.67 (0.25 to 10.07)	1.21 (0.27 to 8.69)	sot

Table 4.48: NMA sensitivity analysis results for the outcome of Serious AEs using the prior distribution Unif(0,5) for the heterogeneity parameter

	pbo	bam	bam_ete	cas_imd	sot
pbo	pbo	1.26 (0.04 to 31.77)	0.56 (0.05 to 6.02)	0.56 (0.08 to 8.17)	0.38 (0.03 to 7.97)
bam	0.79 (0.03 to 23.96)	bam	0.45 (0.01 to 16.80)	0.47 (0.02 to 19.94)	0.32 (0.01 to 27.62)
bam_ete	1.77 (0.17 to 19.21)	2.24 (0.06 to 74.02)	bam_ete	1.02 (0.08 to 24.81)	0.68 (0.02 to 34.42)
cas_imd	1.78 (0.12 to 12.70)	2.11 (0.05 to 47.15)	0.98 (0.04 to 11.88)	cas_imd	0.70 (0.02 to 22.64)
sot	2.61 (0.13 to 32.15)	3.14 (0.04 to 187.89)	1.46 (0.03 to 43.60)	1.43 (0.04 to 58.10)	sot

Table 4.49: NMA sensitivity analysis results for the outcome of Serious AEs using the prior distribution from Turner et al. for the heterogeneity parameter

	pbo	bam	bam_ete	cas_imd	sot
pbo	pbo	1.10 (0.07 to 10.17)	0.58 (0.16 to 1.91)	0.38 (0.19 to 1.50)	0.34 (0.11 to 1.17)
bam	0.91 (0.10 to 14.47)	bam	0.52 (0.06 to 8.64)	0.37 (0.04 to 6.19)	0.31 (0.03 to 6.29)
bam_ete	1.72 (0.52 to 6.10)	1.94 (0.12 to 18.14)	bam_ete	0.68 (0.20 to 3.61)	0.59 (0.11 to 3.50)
cas_imd	2.63 (0.67 to 5.40)	2.71 (0.16 to 23.33)	1.47 (0.28 to 4.92)	cas_imd	0.88 (0.14 to 3.26)
sot	2.93 (0.85 to 9.39)	3.19 (0.16 to 39.54)	1.70 (0.29 to 8.79)	1.13 (0.31 to 7.13)	sot

Table 4.50: NMA sensitivity analysis results for the outcome of Serious AEs using the prior distribution Half-Cauchy(0.5) for the heterogeneity parameter

	pbo	bam	bam_ete	cas_imd	sot
pbo	pbo	1.16 (0.06 to 15.02)	0.57 (0.11 to 2.70)	0.43 (0.15 to 2.94)	0.35 (0.07 to 2.35)
bam	0.86 (0.07 to 16.26)	bam	0.49 (0.04 to 9.88)	0.40 (0.04 to 8.99)	0.31 (0.01 to 9.38)
bam_ete	1.74 (0.37 to 8.99)	2.03 (0.10 to 28.31)	bam_ete	0.78 (0.17 to 7.47)	0.61 (0.07 to 7.87)
cas_imd	2.35 (0.34 to 6.89)	2.49 (0.11 to 25.71)	1.29 (0.13 to 5.84)	cas_imd	0.80 (0.06 to 6.07)
sot	2.88 (0.43 to 14.76)	3.24 (0.11 to 66.81)	1.65 (0.13 to 15.15)	1.25 (0.16 to 16.43)	sot

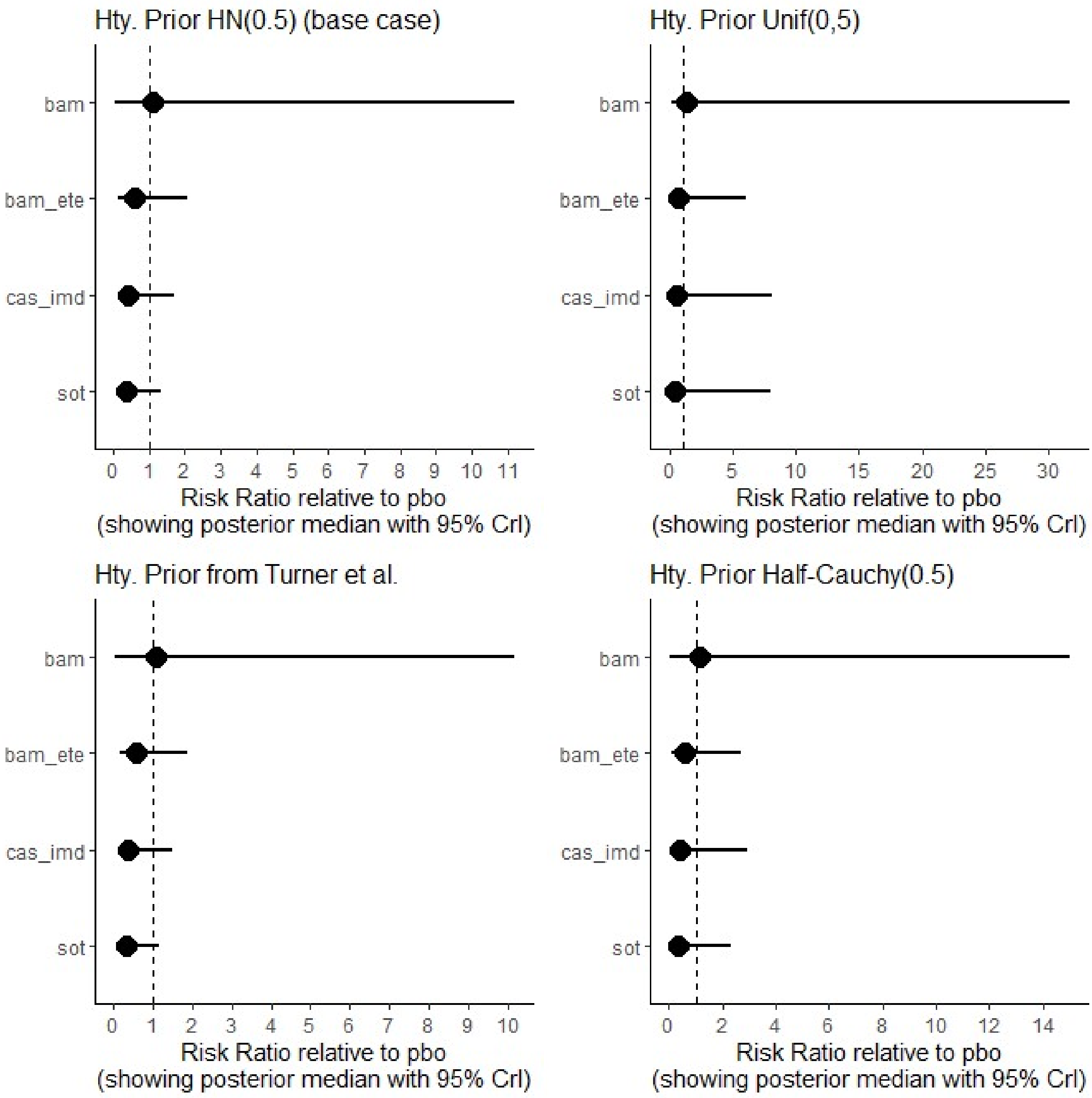

Figure 4.53: Estimated treatment effects for each mAB versus placebo obtained from varying the prior distribution on the heterogeneity parameter sigma, for the outcome of Serious AEs

5.4 Appendix: Investigation of consistency

The only closed loops in the evidence networks arise from the direct comparison between bamlanivimab/etesevimab and casirivimab/imdevimab carried out in (McCreary et al., 2021). The outcomes affected are mortality, hospitalisation, infusion-related AEs and serious AEs. Consistency was assessed by comparing the direct estimate of relative effects of ballanivimab/etesevimab versus casirivimab/imdevimab from (McCreary et al., 2021), and the indirect estimate of the same effect obtained re-running an NMA of all studies evaluating

bamlanivimab/etesevimab and casirivimab/imdevimab with (McCreary et al., 2021) excluded (note that the latter does not contribute to the indirect estimate as there is no placebo arm).

Table 4.51: Direct and Indirect Estimates, casirivimab/imdevimab versus bamlanivimab/etesevimab

Outcome	Direct	Indirect
Mortality	0.72 (0.24, 2.06)	21.42 (1.25, 958.02)
Hospitalisation	0.97 (0.78, 1.20)	1.17 (0.58, 2.43)
Serious AEs	2.09 (0.38, 16.62)	0.26 (0.08, 0.84)

For all three outcomes, 95% credible intervals for the direct and indirect relative treatment effect estimates overlap, inidicating that there is no statistically significant inconsistency in the networks. The results show a numerical difference between the direct and indirect effect estimates for the mortality outcome, which could potentially indicate a violation of the assumption of consistency which was not detected due to low statistical power. Upon examining the raw event counts, there were no deaths in the bamlanivimab/etesevimab arms of Dougan et al. (2021a), compared with one death in each casirivimab/imdevimab arm of (Weinreich et al., 2021). When combined with the weakly informative prior, this results in a very high point estimate of the RR of mortality for casirivimab/imdevimab versus bamlanivimab/etesevimab, although this estimate is highly uncertain due to extremely small event numbers. By contrast, mortality rates observed in (McCreary et al., 2021) were similar for both treatments, giving a relative risk that is close to 1. Due to the low event numbers it is not possible to determine whether this discrepancy has arisen by chance, or indicates a genuine violation of the consistency assumption. This situation is similar for the SAE outcome.

The results for hospitalisation do not indicate any violation of the consistency assumption.

5.5 Appendix: Model diagnostics

```
##
## ### Mortality
```

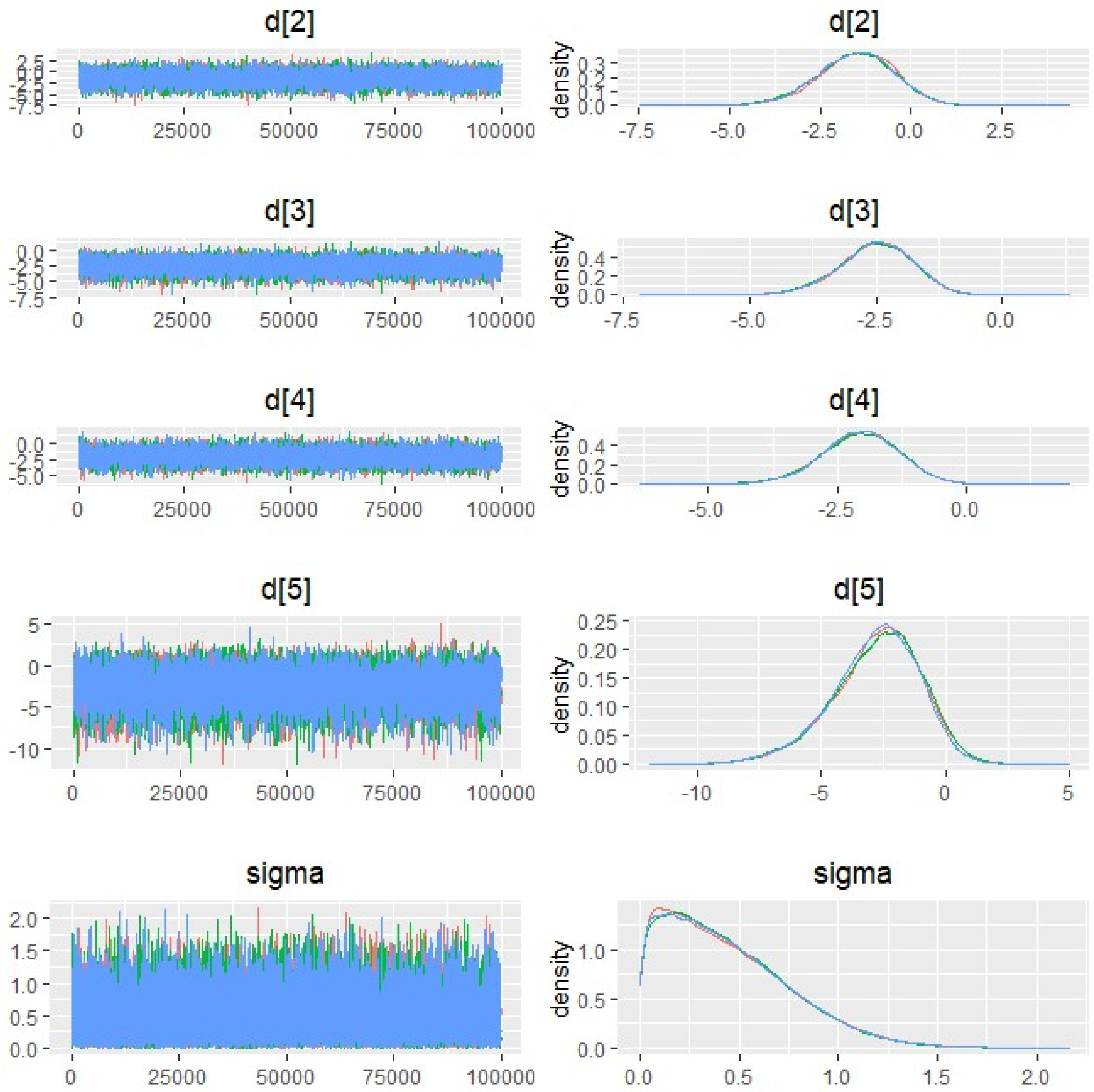


```
## $gelman.rubin
## $psrf
##       Point est. Upper C.I.
## d[2]    1.000540   1.001812
## d[3]    1.000333   1.001190
## d[4]    1.000270   1.000427
## d[5]    1.000742   1.001911
## sigma   1.000175   1.000627
##
## $mpsrf
## [1] 1.000964
##
## attr(,"class")
```

```
## [1] "gelman.rubin.results"
##
## $geweke
## $stats
##           Chain 1    Chain 2    Chain 3
## d[2]   0.76744920 -0.3772772 0.06006038
## d[3]  -1.08528266  0.6057266 0.29232265
## d[4]   0.06156129 -0.4630726 0.26234962
## d[5]   0.81839348  0.8409934 0.79983817
## sigma  1.69986347 -0.5039221 0.19475546
##
## $frac1
## [1] 0.1
##
## $frac2
## [1] 0.5
##
## attr(,"class")
## [1] "geweke.results"
##
##
##
##
##
##
## ### Hospitalisation
```

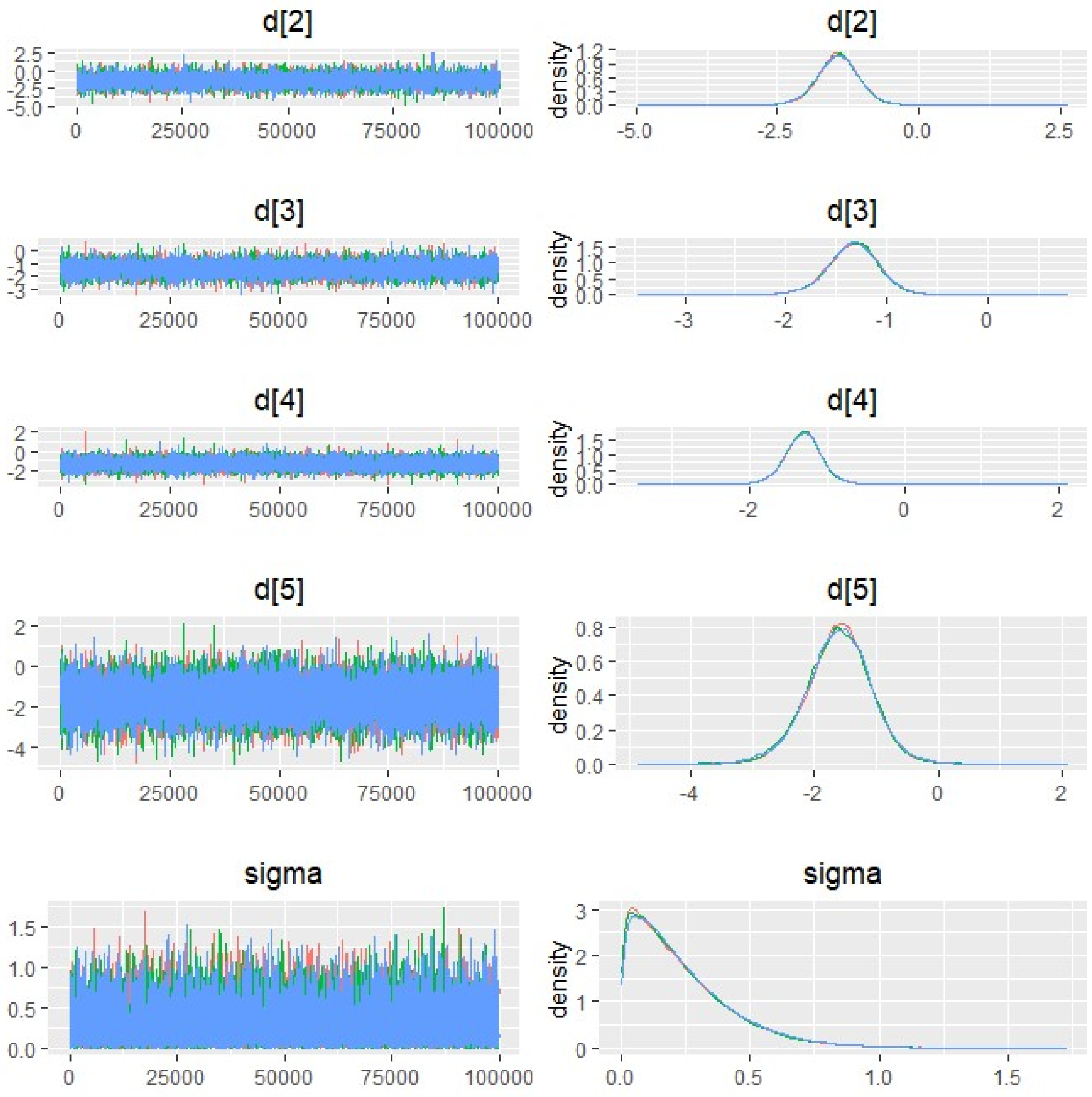


```
## $gelman.rubin
## $psrf
##        Point est. Upper C.I.
## d[2]    1.000375   1.000878
## d[3]    1.000162   1.000455
## d[4]    1.000201   1.000533
## d[5]    1.000224   1.000618
## sigma   1.000475   1.001760
##
## $mpsrf
## [1] 1.001056
##
## attr(,"class")
```

```
## [1] "gelman.rubin.results"
##
## $geweke
## $stats
##          Chain 1    Chain 2    Chain 3
## d[2]   0.7896875  1.1819564  1.3795317
## d[3]   0.7353951  0.5454311 -0.7832502
## d[4]   0.9172696  1.0722359 -0.7812157
## d[5]  -0.5033228 -0.1167988  2.6646940
## sigma -0.5039929  0.3088583  0.5573365
##
## $frac1
## [1] 0.1
##
## $frac2
## [1] 0.5
##
## attr(,"class")
## [1] "geweke.results"
##
##
##
##
##
##
## ### Invasive Ventilation
```

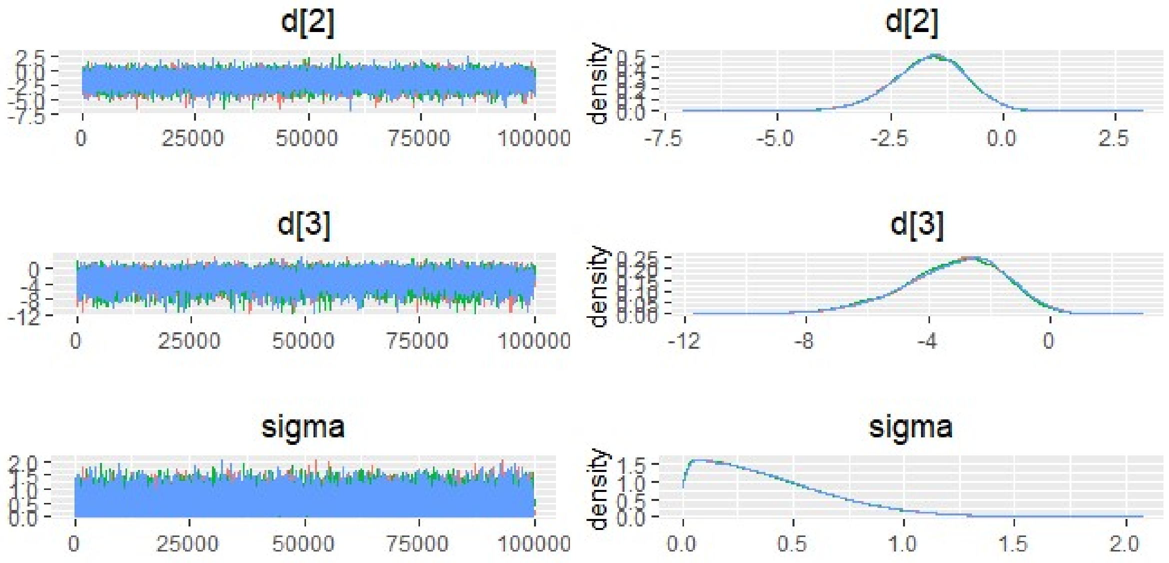


```
## $gelman.rubin
## $psrf
##       Point est. Upper C.I.
## d[2]    1.000188   1.000657
```

```
## d[3]     1.000569    1.001964
## sigma    1.000081    1.000269
##
## $mpsrf
## [1] 1.000617
##
## attr(,"class")
## [1] "gelman.rubin.results"
##
## $geweke
## $stats
##            Chain 1    Chain 2    Chain 3
## d[2]  -0.5798184  0.3931644  1.3349431
## d[3]   0.5637790 -0.4126955 -1.2926784
## sigma  0.6745141 -1.4093452 -0.5052508
##
## $frac1
## [1] 0.1
##
## $frac2
## [1] 0.5
##
## attr(,"class")
## [1] "geweke.results"
##
##
##
##
##
##
## ### ICU Admission
```

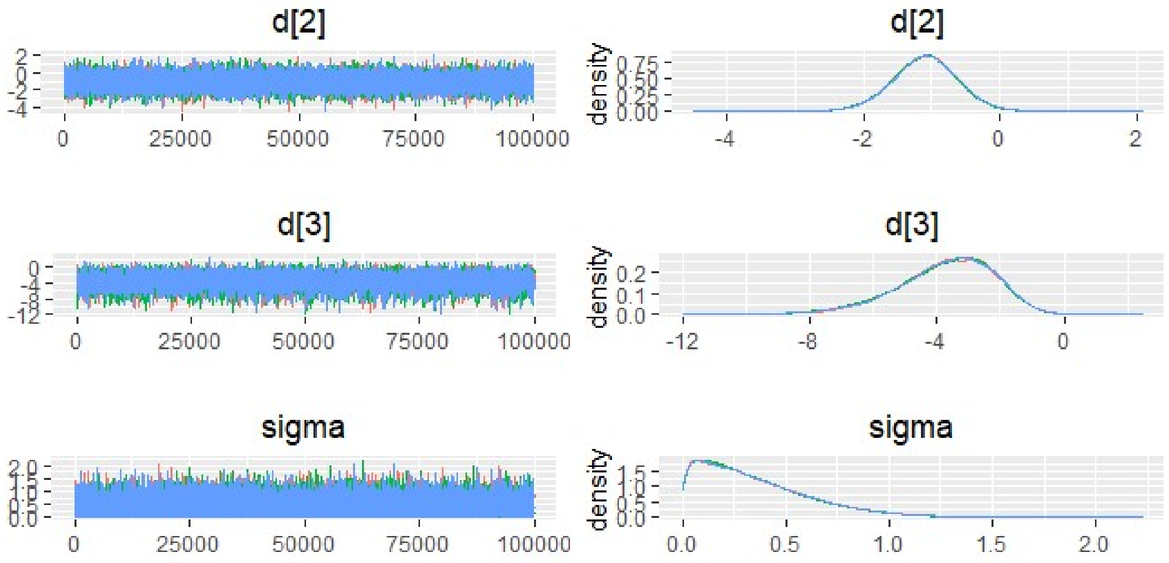

```
## $gelman.rubin
## $psrf
##       Point est. Upper C.I.
## d[2]    1.000360   1.001222
## d[3]    1.000737   1.002072
## sigma   1.000081   1.000303
##
## $mpsrf
## [1] 1.000575
##
## attr(,"class")
## [1] "gelman.rubin.results"
##
## $geweke
## $stats
##         Chain 1     Chain 2    Chain 3
## d[2]   2.833801 -1.55548164 -1.0389145
## d[3]  -1.081475  0.77202066  0.6404850
## sigma  2.211937  0.03912771 -0.9238902
##
## $frac1
## [1] 0.1
##
## $frac2
## [1] 0.5
##
## attr(,"class")
## [1] "geweke.results"
##
##
##
##
##
##
## ### Infusion-related AEs
```

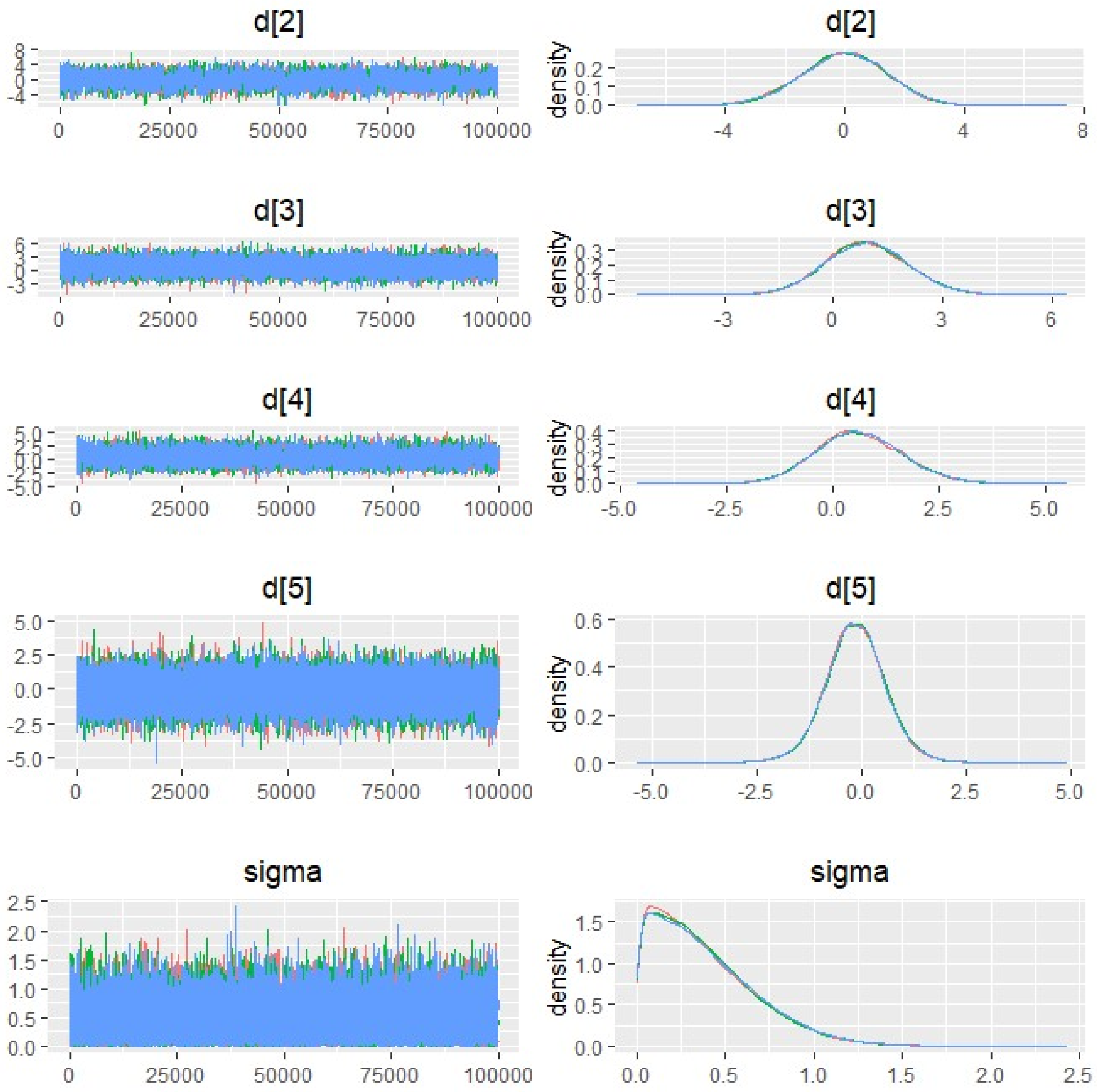


```
## $gelman.rubin
## $psrf
##       Point est. Upper C.I.
## d[2]    1.000711   1.002006
## d[3]    1.000102   1.000263
## d[4]    1.000127   1.000396
## d[5]    1.000350   1.001296
## sigma   1.000243   1.000499
##
## $mpsrf
## [1] 1.00099
##
## attr(,"class")
```

```
## [1] "gelman.rubin.results"
##
## $geweke
## $stats
##           Chain 1    Chain 2     Chain 3
## d[2]   0.26330992  0.1862991  1.38429119
## d[3]   0.28380474  0.8943063 -0.17529491
## d[4]  -0.07028057  0.6708911  0.05830988
## d[5]   0.11504257 -0.5503383  0.93321909
## sigma -0.23224453 -0.3151971 -0.35377794
##
## $frac1
## [1] 0.1
##
## $frac2
## [1] 0.5
##
## attr(,"class")
## [1] "geweke.results"
##
##
##
##
##
##
## ### Serious AEs
```

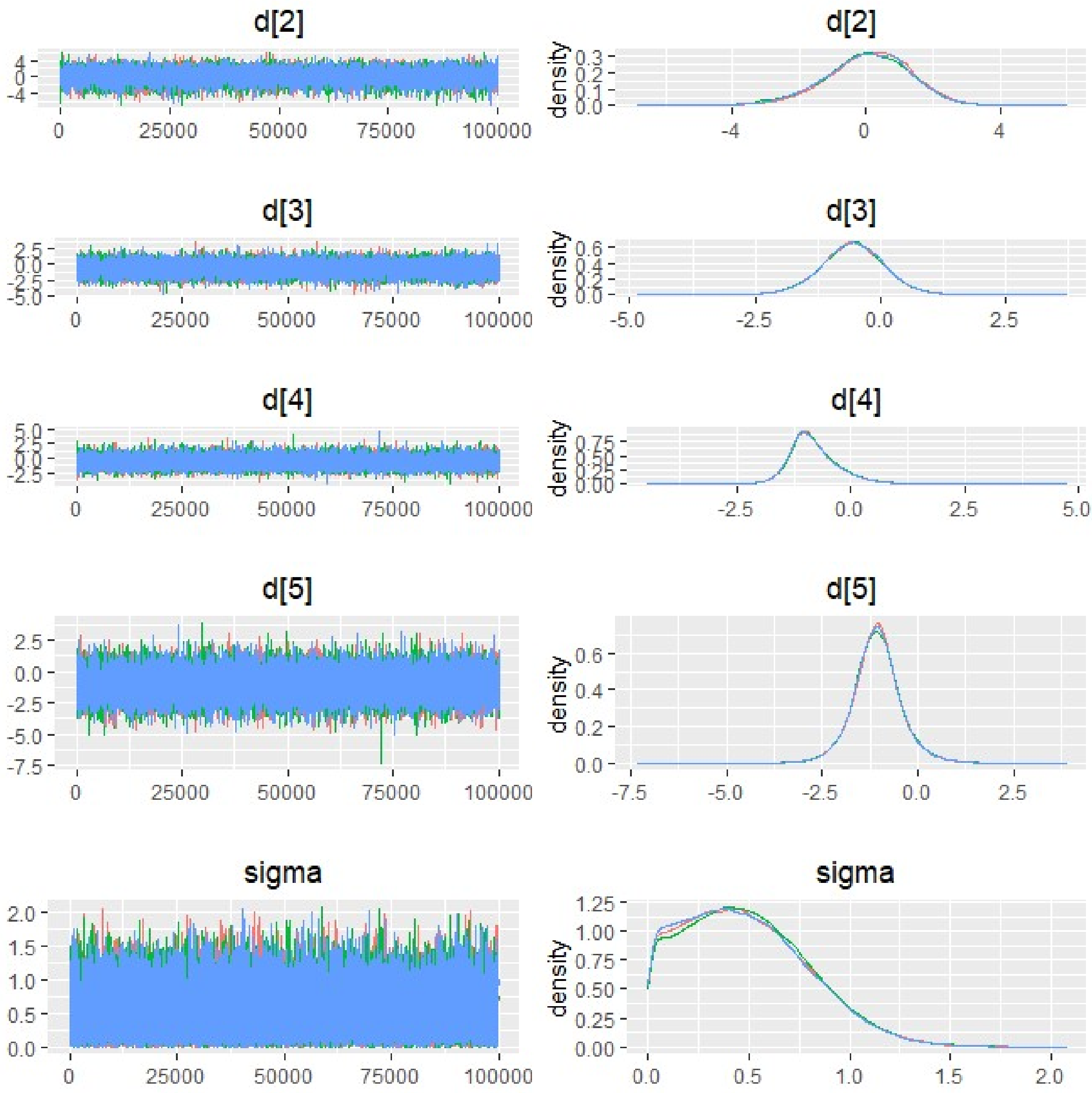


```
## $gelman.rubin
## $psrf
##        Point est. Upper C.I.
## d[2]    1.003796   1.012137
## d[3]    1.000086   1.000301
## d[4]    1.000047   1.000167
## d[5]    1.000041   1.000154
## sigma   1.000027   1.000090
##
## $mpsrf
## [1] 1.003435
##
## attr(,"class")
```

```
## [1] "gelman.rubin.results"
##
## $geweke
## $stats
##          Chain 1    Chain 2   Chain 3
## d[2]   0.5711485  0.5916506  1.451306
## d[3]   1.5138539 -1.9163990  1.912189
## d[4]  -0.3942414  0.8652241 -1.956407
## d[5]  -1.7094835 -0.7397251 -1.056998
## sigma -0.1503539  0.9546424 -2.359580
##
## $frac1
## [1] 0.1
##
## $frac2
## [1] 0.5
##
## attr(,"class")
## [1] "geweke.results"
```

References

Béliveau A, Boyne DJ, Slater J, Brenner D, Arora P. BUGSnet: An r package to facilitate the conduct and reporting of bayesian network meta-analyses. BMC Medical Research Methodology 2019;19. https://doi.org/10.1186/s12874-019-0829-2.

Dias S, Sutton AJ, Ades AE, Welton NJ. Evidence synthesis for decision making 2. Medical Decision Making 2013a;33:607–17. https://doi.org/10.1177/0272989x12458724.

Dias S, Welton NJ, Sutton AJ, Ades AE. Evidence synthesis for decision making 5. Medical Decision Making 2013b;33:657–70. https://doi.org/10.1177/0272989x13485155.

Dougan M, Azizad M, Mocherla B, Gottlieb RL, Chen P, Hebert C, et al. A randomized, placebo-controlled clinical trial of bamlanivimab and etesevimab together in high-risk ambulatory patients with COVID-19 and validation of the prognostic value of persistently high viral load. Clinical Infectious Diseases 2021a. https://doi.org/10.1093/cid/ciab912.

Dougan M, Nirula A, Azizad M, Mocherla B, Gottlieb RL, Chen P, et al. Bamlanivimab plus etesevimab in mild or moderate covid-19. New England Journal of Medicine 2021b;385:1382–92. https://doi.org/10.1056/nejmoa2102685.

Eom JS, Ison M, Streinu-Cercel A, Săndulescu O, Preotescu L-L, Kim Y-S, et al. Efficacy and safety of CT-P59 plus standard of care: A phase 2/3 randomized, double-blind, placebo-controlled trial in outpatients with mild-to-moderate SARS-CoV-2 infection 2021. https://doi.org/10.21203/rs.3.rs-296518/v1.

Günhan BK, Röver C, Friede T. Random-effects meta-analysis of few studies involving rare events. Research Synthesis Methods 2020;11:74–90. https://doi.org/10.1002/jrsm.1370.

Gupta A, Gonzalez-Rojas Y, Juarez E, Casal MC, Moya J, Falci DR, et al. Effect of the neutralizing SARS-CoV-2 antibody sotrovimab in preventing progression of COVID-19: A randomized clinical trial. medRxiv 2021. https://doi.org/10.1101/2021.11.03.21265533.

McCreary EK, Bariola JR, Minnier T, Wadas RJ, Shovel JA, Albin D, et al. A learning health system randomized trial of monoclonal antibodies for covid-19 2021. https://doi.org/10.1101/2021.09.03.21262551.

Röver C, Bender R, Dias S, Schmid CH, Schmidli H, Sturtz S, et al. On weakly informative prior distributions for the heterogeneity parameter in bayesian random-effects meta-analysis. Research Synthesis Methods 2021;12:448–74. https://doi.org/10.1002/jrsm.1475.

Turner RM, Davey J, Clarke MJ, Thompson SG, Higgins JP. Predicting the extent of heterogeneity in meta-analysis, using empirical data from the cochrane database of systematic reviews. International Journal of Epidemiology 2012;41:818–27. https://doi.org/10.1093/ije/dys041.

Warn DE, Thompson SG, Spiegelhalter DJ. Bayesian random effects meta-analysis of trials with binary outcomes: Methods for the absolute risk difference and relative risk scales. Statistics in Medicine 2002;21:1601–23. https://doi.org/10.1002/sim.1189.

Weinreich DM, Sivapalasingam S, Norton T, Ali S, Gao H, Bhore R, et al. REGEN-COV antibody combination and outcomes in outpatients with covid-19. New England Journal of Medicine 2021;385:e81. https://doi.org/10.1056/nejmoa2108163.
